# Urinary Exosomes from Bladder Cancer Patients Show a Residual Cancer Phenotype despite Complete Pathological Downstaging

**DOI:** 10.1038/s41598-020-62753-x

**Published:** 2020-04-06

**Authors:** Stefanie Hiltbrunner, Michael Mints, Maria Eldh, Robert Rosenblatt, Benny Holmström, Farhood Alamdari, Markus Johansson, Rosanne E. Veerman, Ola Winqvist, Amir Sherif, Susanne Gabrielsson

**Affiliations:** 1 Division of Immunology and Allergy, Department of Medicine Solna, Karolinska Institute, Stockholm, Sweden; 2 Department of Urology, Södersjukhuset, Stockholm, Sweden; 3 Department of Urology, Akademiska University Hospital, Uppsala, Sweden; 4 Department of Urology, Västmanland Hospital, Västerås, Sweden; 5 Department of Urology, Sundsvall Hospital, Sundsvall, Umeå University, Umeå, Sweden; 6 Department of Clinical Immunology and Transfusion Medicine, Karolinska University Hospital, Solna, Sweden; 7 Department of Surgical and Perioperative Sciences, Urology and Andrology, Umeå University, Umeå, Sweden

**Keywords:** Bladder cancer, Tumour immunology

## Abstract

Invasive urinary bladder cancer shows high recurrence rates after cystectomy even with apparent complete downstaging at cystectomy. Exosomes are nano-sized vesicles important in cell-cell communication, which have been hypothesized to contribute to cancer dissemination and recurrence. The aim of this study was to investigate if pro-carcinogenic exosomes could be detected in urine from histologically downstaged bladder cancer patients. 13 Patients were included in this study. Paired ureter and urine samples from nine patients underwent mass spectrometry, while samples from the remaining patients were used for exosome characterization. At cystectomy, exosomes were isolated from bladder and ureter urine, whereafter quantitative proteome profiling was performed. Urinary exosomes clustered based on whether they came from the bladder, with tumour contact, or the ureters, without tumour contact, even though all came from completely downstaged patients. Proteins overexpressed in exosomes derived from bladder urine contained several oncogenes and were mainly associated with tumour metabolism pathways. Although patients were histologically tumour-free at cystectomy, the bladder urine contained exosomes with a carcinogenic metabolic profile. This suggests a continuous release of exosomes from the bladder, which may promote recurrence at distant sites through metabolic rewiring, even after apparent complete downstaging. These exosomes, coming from either undetected cancer cells or partly transformed cells, are likely to increase the risk of metastasis and encourages cystectomy even in completely downstaged patients.

## Introduction

Invasive urinary bladder cancer (UBC) metastasises rapidly, mainly through the draining regional lymphatic system^1^, and there is evidence of establishment of pre-metastatic niches in the lymph nodes long before actual metastasis occurs^2^. Early micro-metastatic dissemination in UBC is mainly evidenced by the fact that patients with organ-confined disease (pT2N0) suffer recurrence rates of up to 40% after radical cystectomy (RC)^3^. Also, it has been shown that complete down-staging (pT0) through primary transurethral resection (TUR-B) plus neoadjuvant cisplatin-based combination chemotherapy (NAC) and RC as the radical surgical treatment, substantially improves survival, presumably as an effect on disseminated tumour cells. In the same study, completely downstaged patients, who did not receive chemotherapy, showed significantly poorer overall survival, suggesting the presence of undetected cancer cells or tumour-promoting factors^4^.

Exosomes are nano-sized vesicles, derived from the late endosomal compartment, acting as messengers between cells through the transfer of biomolecules^5^. Exosomes are produced by most cell types and found in bodily fluids, including urine^6^. Tumour-derived exosomes play an important role in carcinogenesis, tissue remodelling and metastasis^7–9^. They can induce apoptosis of immune cells^10^ and stimulate regulatory T-cells^11^, leading to immune evasion. Moreover, urine-derived exosomes from high-grade UBC patients have been shown to promote cell migration^12^. Thus, exosomes produced from remaining cancer cells or transformed tissue may promote recurrence, and provide an attractive source of diagnostic and therapeutic markers in UBC.

Given the high recurrence rate in seemingly cancer-free patients, we aimed to trace cancer-related exosomes in downstaged urinary bladder cancer patients. This was achieved through proteomic analysis of urinary exosomes derived from the bladder and from the ureter of UBC patients at RC. By integrating proteomics and pathway analyses of UBC exosomes, we provide evidence of carcinogenic exosomes that are released into the bladder in patients with no macroscopic tumour left after primary TUR-B followed by NAC and with evaluation of the completely excised urinary bladder specimen post-RC. These findings challenge the concept of complete histopathological downstaging in urinary bladder cancer after combined tumour ablation as a hallmark of a non-cancerous urinary bladder.

## Methods

### Patients

Thirteen patients with invasive UBC, scheduled for radical cystectomy (RC), were prospectively recruited for the study in 2014–2015 at five Swedish departments of urology (Norrland University, Akademiska, Sundsvall, Västmanland and Gävle Hospitals). The patient data are found in Table 1. For the nine patients where paired samples were obtained, mass spectrometry was performed. Out of these nine patients, six were male and three were female. Following TUR-B, eight were staged cT2N0M0G3 and one high risk cT1N0M0G3. Six patients (four male and two female) received NAC preceding RC, and of these five had complete pathological down-staging (CD) i.e. pT0N0. Two patients had remaining tumour in the bladder. Three of the patients had concomitant prostate cancer. At RC, tumour site tissue went for histopathological analysis and staging. All patients were recurrence-free as of 11/04/2019.

**Table 1:** Patient Characteristics.

Patient	Preoperative clinical stage	Staging post-cystectomy	Gender	Age	NAC/noNAC	Number of Cycles	Response	Additional Information
1^†^	cT2N0M0,G3	pT2N0M0**	male	76	noNAC	0	/	
2^†§¤^	cT2N0M0,G3*	pT0N0M0	female	69	NAC	1	CR	
3^†^	cT2N0M0,G3	pT0N0M0	male	39	NAC	4	CR	
4^†^	cT2N0M0,G3	pT0N0M0	male	66	NAC	3	CR	Prostatic cancer Gleason score (3 + 4 = 7)
5^†^	cT2N0M0,G3	pT0N0M0	female	79	NAC	3	CR	
6^†^	cT2N0M0,G3	pT0N0M0	female	77	noNAC	0	/	
7^†#§^	cT2N0M0,G3	pT2bN0M0**	male	65	NAC	4	SD	
8^†^	cT2N0M0,G3	pT0N0M0	male	76	NAC	3	CR	Prostatic cancer Gleason score (3 + 3 = 6)
9^†^	cT1N0M0,G3	pT0N0M0	male	57	noNAC	0	/	Prostatic cancer Gleason score (3 + 3 = 6)
10^#§^	cT2N0M0,G3	pT0N0M0	male	73	NAC	3	CR	
11^§^	cT2N0M0,G2	pT0N0M0	female	67	NAC	3	CR	
12^§^	cT2N0M0,G3	pT0N0M0	male	66	NAC	3	CR	
13^§^	cT2N0M0,G3	pT0N0M0	male	73	NAC	3	CR	

CR = Complete Response; SD = Stable Disease; *In addition to the solid tumour, the patient also had concomitant CIS (Cancer *in Situ*); **Remaining tumors in the bladder and therefore excluded from mass spectrometry analysis; ^†^Mass spectrometry; ^#^Electron microscopy; ^§^Nanoparticle Tracking Analysis; ^¤^Flow cytometry.

Urine was obtained from the bladder prior to surgery, and directly from the ureters after transection, by introduction of Ch.8 baby feeding catheters to the renal pelvises. All samples were shipped and processed on the day of cystectomy. All methods were carried out in accordance with relevant guidelines and regulations and all experimental protocols were approved by the Regional Ethical Review Board in Stockholm (original no.: 2007/71-31), and all patients were above 18 and gave written and oral informed consent.

### Exosome isolation

Urine samples were spun at 3000 g for 30 min and filtered through a 0.22 µm filter. Exosomes were isolated by ultracentrifugation at 100 000 g for 2 h, washed with PBS, resuspended in PBS and stored at −80 °C. Protein concentration was measured by DC protein assay (Bio-Rad).

### Flow cytometry

30 µl sulfate-aldehyde latex beads (4 μm, 1.3 × 10^9^ beads/ml, Invitrogen) were incubated with 30 µg anti-CD63 antibody (H5C6, BD Pharmingen) for 30 min at RT and rotated overnight at RT. Beads were blocked with 100 mM glycine for 30 min and washed with 0.5% BSA/PBS. Exosomes were bound to anti-CD63 coated beads with 1.25 µg exosomes per µl beads and phenotyped as described^13^. Antibodies used (dilution 1:100): isotype control mouse IgG1 FITC (MOPC-21, Biolegend) and anti-human CD9 FITC (M-L13, BD Pharmingen), CD63 FITC (H5C6, Biolegend), CD81 FITC (5A6, Biolegend). Beads were analysed on a FACS Calibur (BD Bioscience) by FlowJo software (TreeStar Inc.).

### Nanoparticle tracking analysis

Exosome size was determined with the Nanosight LM10HSB system. Vesicles were measured at circa 45 particles/frame and 2 × 10^8^ to 8 × 10^8^ particles/ml. Three independent samples were run 5 times each for 60 seconds with a camera level of 9 and screen gain of 3 with a syringe pump speed of 50.

### Electron microscopy

3 µL from each sample was added to a grid with a glow-discharged carbon-coated supporting film for 3 minutes. The grid was rinsed by adding 5 µL distilled water. Water was soaked off by a filter paper and the grid stained with 5 µL 1% uranyl acetate in water for 7 seconds. Samples were examined in a Hitachi HT 7700 electron microscope at 80 kV and digital images were taken by a Veleta camera (Olympus,).

### Mass spectrometry

18 urinary exosome samples (9 ureter and 9 bladder) underwent mass spectrometry. Proteins were extracted in a urea-containing buffer using a sonication bath. Total protein concentration was measured using Bradford assay (Bio-Rad). Proteins were reduced, alkylated and digested with trypsin. Finally, samples were purified on Pierce C18 Spin Columns (ThermoScientific), dried and resolved in 0.1% FA to a concentration of 0.3 μg/μL. Peptides were separated in reversed-phase on a C18-column, using a 90 min gradient and electrosprayed onto a Q-Exactive Plus Orbitrap mass spectrometer (ThermoFinnigan). Tandem mass spectrometry was performed applying HCD collision-induced dissociation.

For identification, database searches were performed using the Mascot algorithm embedded in Proteome Discoverer 1.4 (ThermoScientific) against proteins from Homo Sapiens extracted from UniProtKB (January, 2016). A decoy search database, including common contaminants and a reverse database, was used to estimate the identification false discovery rate. The search criteria for identification were set to at least two matching peptides of 95% confidence per protein. For quantification, a label-free intensity analysis was performed for each sample.

### Statistics and network analysis

The clustering of all samples, including the bladder urine from the two patients with residual disease, is found in Supplementary Fig. 1. As this study focused on bladder urine in completely downstaged patients, the bladder urine exosomes from the patients with residual disease were removed from downstream analysis. The ureter urine samples from these patients were kept as controls in order to increase statistical power. Upon PCA, these samples did not differ from ureter exosomes taken from tumour-free patients (Supplementary Fig. 2). Protein expression values were log-transformed, and an outlier sample, identified through PCA, was removed (Supplementary Fig. 3). PCA was performed using the FactomineR package in R^14^ with exosome origin, gender, concomitant prostate cancer and NAC treatment as qualitative supplementary variables and age and number of NAC cycles as quantitative supplementary variables. T-tests were adjusted for multiple testing with Benjamini-Hochberg correction. Pathway analysis was performed through network set enrichment analysis (NSEA), using Enrichnet^15^. STRING^16^ provided the interaction network and functional pathways were taken from KEGG^17^.

A protein interaction network was constructed in Cytoscape v 3.6.1^18^ using all identified proteins as nodes, and the calculated spearman correlation coefficients between each protein’s expression values as edges. Clustering was done with the clusterMaker Cytoscape plugin, using the gLay community cluster algorithm^19^. Pathway analysis of clusters in the cytoscape network was performed through network set enrichment analysis (NSEA) using the JEPETTO plugin^20^. In these clusters, only proteins with a log-fold expression change of >0.2 between bladder and ureter were subjected to network analysis.

## Results

**Figure 1:**
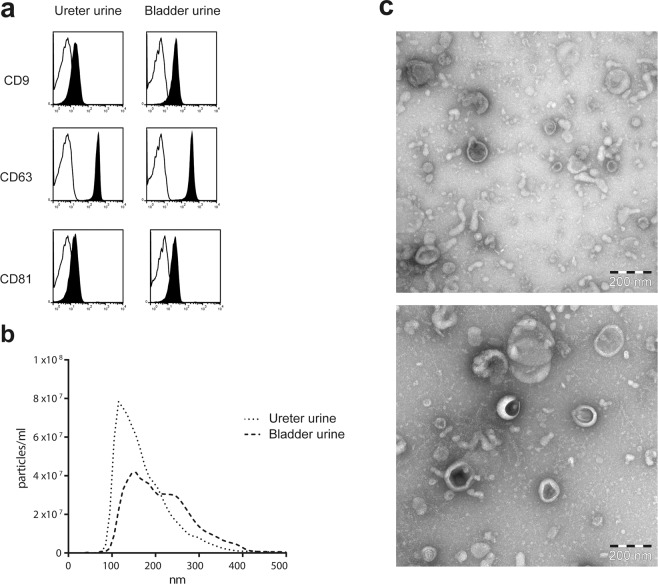
Phenotypic analysis of urine- derived extracellular vesicles. (**a**) Exosomes were bound to anti-human CD63 latex beads, stained for CD9, CD63 and CD81 and analysed by flow cytometry. Data are shown as representative histograms (black represents marker, line the corresponding isotype control). (**b**) Size distribution of urine -derived exosomes measured by nanoparticle tracking analysis, all exosomes analysed showed a mode size typical for exosomes, bladder urine 155 nm, ureter urine 115 nm. (**c**) EM picture of exosomes from bladder urine (top) and ureter urine (bottom), bar equals 200 nm.

Firstly, we investigated whether the isolated extracellular vesicles (EV) displayed an exosome-like phenotype. Flow cytometry of EVs bound to anti-CD63 coated latex beads showed expression of tetraspanins CD9, CD63 and CD81 in all samples (Fig. 1a). Nanoparticle tracking analysis and electron microscopy showed size distribution and morphology typical for exosomes (Fig. 1b,c). Bladder urine and ureter urine yielded 0.8 µg exosomal protein/mL urine and 0.67 µg exosomal protein/mL urine (median) respectively, with no significant difference between sample type. In addition, western blot analysis showed that EVs were negative for the endoplasmatic reticulum marker calnexin (data not shown). Furthermore, proteomic analysis showed exosomal markers such as Rab proteins, annexins and heat shock proteins (Supplementary Table 1). These data demonstrate that the urinary EVs have an exosomal phenotype without contamination of ER-derived cellular debris, thus they are hereafter referred to as exosomes.

**Figure 2:**
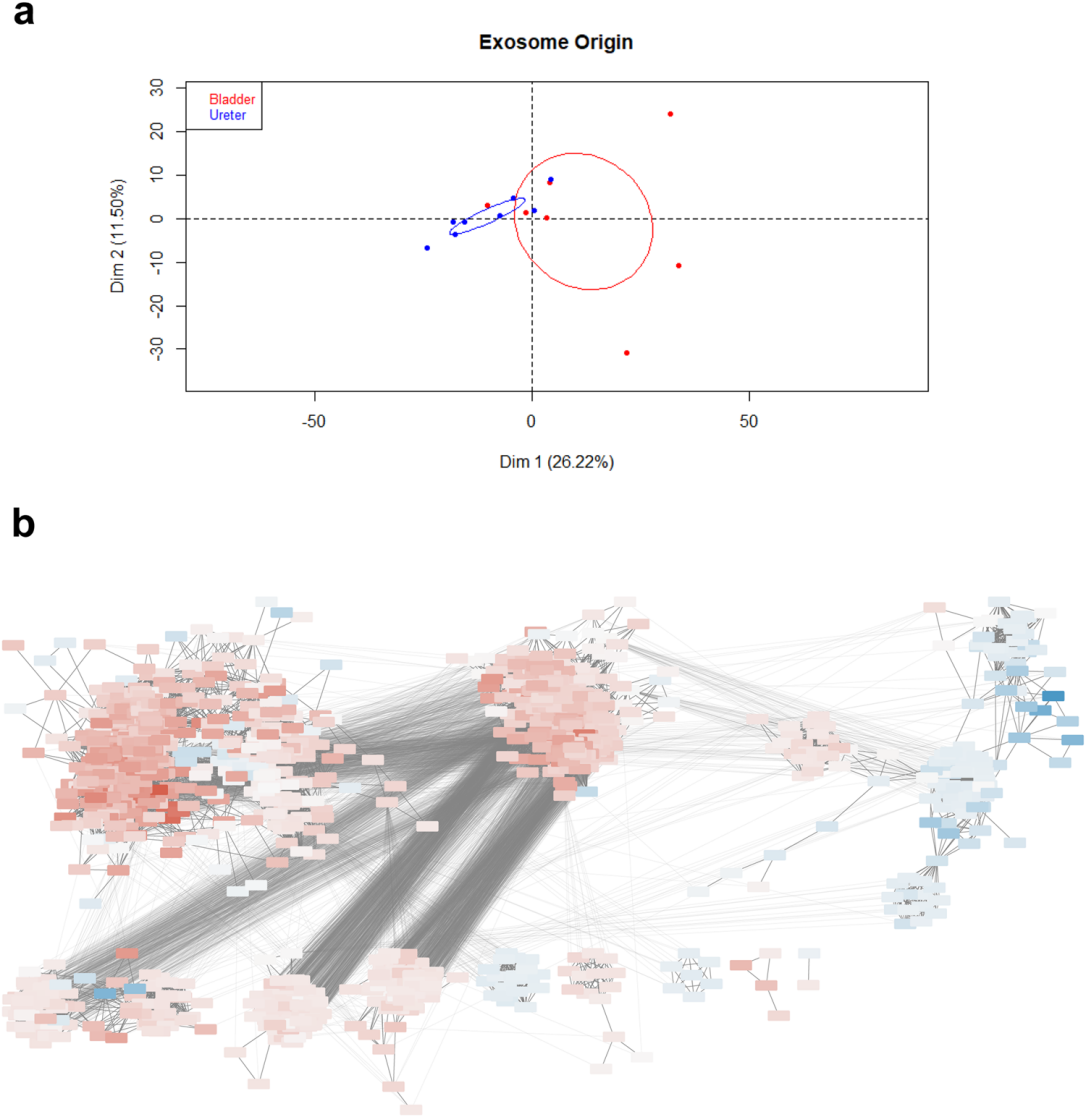
Separation of samples according to exosome origin. (**a**) PCA on urine-derived samples only shows significant separation between bladder/ureter urine in the 1st dimension. Ellipses represent 95% CI. (**b**) Clusters from protein correlation network. Red denotes higher expression in bladder and blue higher in ureter urine. The more intense the colour, the larger the expression difference. The three largest clusters are numbered.

From the nine patients that were analyzed by mass spectrometry, excluding the removed outlier and the bladder urine from patients with residual disease, in total 1094 proteins were identified in urinary exosomes – 403 unique to bladder urine and 120 to ureter urine. PCA showed a clear separation between urinary exosome samples from bladders and ureters (Fig. 2a). Separation in the 1st dimension was based on whether exosomes came from the bladder or ureters (p = 0.008), while the 2nd dimension correlated with concomitant prostate cancer (p = 0.04). Thus, proteins that correlated significantly with 1st dimension separation could only be explained by the physical origin of the exosomes and none of the other clinical characteristics (Table 2, Supplementary Fig. 1). We also performed differential expression testing for all proteins across the different clinical parameters and found very few differences. No proteins showed significant differential expression in patients with prostate cancer. In men; envoplakin, periplakin and uroplakin 1A were all lower than in women. Patients receiving NAC had significantly higher levels of FTR and HRG, and lower levels of ITGA3 (Supplementary Table 2).

**Table 2:** Categories showing significant separation on PCA.

Dim.1 category	R²	P-value
Bladder/ureter	0.4246892	0.00848231
**Dim.2 category**	**R²**	**P-value**
Concomitant prostate cancer	0.2836727	0.04094804

487 proteins were significant descriptors of bladder urine in that they correlated significantly with bladder origin in the PCA (Supplementary Table 1). These proteins were subjected to network set enrichment analysis (NSEA). We found that bladder urine showed several enriched pathways, mainly representing cancer metabolism, such as glycolysis and gluconeogenesis, but also inflammatory pathways (Table 3).

**Table 3:** Network set enrichment analysis.

Pathways enriched in proteins correlating with bladder urine on PCA	XD-score	Fisher	Dataset	Pathway	Overlap
hsa04964:Proximal tubule bicarbonate reclamation	2,86	7,70E-05	460	21	8
hsa04966:Collecting duct acid secretion	2,67	5,08E-05	460	25	9
hsa00620:Pyruvate metabolism	1,90	5,21E-05	460	40	11
hsa00030:Pentose phosphate pathway	1,85	2,21E-03	460	26	7
hsa00010:Glycolysis / Gluconeogenesis	1,75	2,02E-06	460	62	16
hsa00480:Glutathione metabolism	1,68	1,09E-04	460	44	11
hsa00360:Phenylalanine metabolism	1,68	4,80E-02	460	16	4
hsa00051:Fructose and mannose metabolism	1,61	1,95E-03	460	33	8
hsa04614:Renin-angiotensin system	1,55	5,60E-02	460	17	4
hsa05110:Vibrio cholerae infection	1,33	5,49E-04	460	52	11
hsa05130:Pathogenic Escherichia coli infection	1,16	2,14E-03	460	52	10
hsa00630:Glyoxylate and dicarboxylate metabolism	1,12	2,08E-01	460	16	3
hsa00770:Pantothenate and CoA biosynthesis	1,12	2,08E-01	460	16	3
hsa00740:Riboflavin metabolism	1,12	2,08E-01	460	16	3
hsa05120:Epithelial cell signaling in Helicobacter pylori infection	1,09	8,35E-04	460	65	12
**Pathways enriched in cluster 1**	**XD-score**	**Fisher q-value**	**Gene set size**	**Pathway size**	**Overlap size**
hsa04614:Renin-angiotensin system	2,75	4,82E-04	366	17	6
hsa04610:Complement and coagulation cascades	2,57	3,27E-14	366	69	23
hsa04964:Proximal tubule bicarbonate reclamation	1,71	1,23E-02	366	21	5
hsa05020:Prion diseases	1,37	4,26E-03	366	35	7
hsa00010:Glycolysis / Gluconeogenesis	1,31	5,86E-05	366	62	12
hsa05322:Systemic lupus erythematosus	1,25	1,17E-06	366	91	17
hsa04670:Leukocyte transendothelial migration	1,24	4,36E-08	366	113	21
hsa04520:Adherens junction	1,19	5,72E-05	366	72	13
hsa05146:Amoebiasis	1,16	1,17E-06	366	102	18
hsa05100:Bacterial invasion of epithelial cells	1,03	6,88E-04	366	68	11
**Pathways enriched in cluster 2**	**XD-score**	**Fisher q-value**	**Gene set size**	**Pathway size**	**Overlap size**
hsa04966:Collecting duct acid secretion	2,31	3,42E-05	191	25	7
hsa00480:Glutathione metabolism	1,22	1,00E-03	191	44	7
hsa05110:Vibrio cholerae infection	1,18	4,30E-04	191	52	8
hsa00030:Pentose phosphate pathway	1,18	3,37E-02	191	26	4
hsa00051:Fructose and mannose metabolism	1,16	1,18E-02	191	33	5
hsa00620:Pyruvate metabolism	1,14	3,78E-03	191	40	6
hsa00040:Pentose and glucuronate interconversions	1,13	3,51E-02	191	27	4
**Pathways enriched in cluster 3**	**XD-score**	**Fisher q-value**	**Gene set size**	**Pathway size**	**Overlap size**
hsa03050:Proteasome	2,18	5,04E-11	99	43	11
hsa05412:Arrhythmogenic right ventricular cardiomyopathy (ARVC)	0,99	4,36E-06	99	73	9

In the protein correlation network (Fig. 2b), three main clusters were formed – two heavily interconnected clusters with proteins mainly overexpressed in bladder as opposed to ureters, and one separate cluster – formed from three smaller ones – with proteins overexpressed in ureters. Upon NSEA, cluster 1 turned out to represent mainly inflammatory pathways, while cluster 2 was enriched for metabolic pathways and cluster 3, which contained proteins overexpressed in ureters, was enriched for proteasome proteins but no pathways involved in tumour signalling.

All proteins contained in these clusters are found in Supplementary Table 2, and the enriched pathways are found in Table 3.

The top 50 overexpressed proteins in bladder and ureter urine, respectively, also underwent NSEA. While no pathways were significantly enriched, due to the low number of proteins, the top three pathways enriched in bladder urine were metabolic. Interestingly, complement activation was enriched in both the ureter and bladder exosomes (Supplementary Table 4).

Differential expression of individual proteins from urinary exosomes from the bladders and ureters was studied to identify potential biomarkers for remaining malignant potential despite complete downstaging. SLC4A1 was underexpressed in exosomes from bladder urine compared to the ureter urine, while 40 proteins were significantly overexpressed, including known oncogenes such as TPP1, TMPRSS2 (transmembrane protease serine 2), FOLR1 (folate receptor 1), RALB and RAB35 (Supplementary Table 5). As validation, the FOLR1 protein could also be detected in exosomes by western blot in three out of three patients, while it could not be detected in whole protein extracts from the same patients’ urine (data not shown).

## Discussion

In order to design novel treatments to combat the high recurrence rates in UBC, a comprehensive understanding of the metastatic mechanisms is needed. Exosomes, being able to carry molecular information from cancer cells to distant tissues, and remodelling these tissues to create a pre-metastatic niche, are prime study objects to understand metastasis. This study is the first to compare the proteomic profiles of urine-derived exosomes from the bladder and the ureter of the same patients in order to study whether they could explain tumour recurrence despite complete histopathological downstaging.

During cystectomy, urine was collected from the ureters and compared with urine that had passed through the bladder. Interestingly, we identified a unique proteomic profile in bladder-derived exosomes, which was enriched for pathways involved in metabolic remodelling. This altered exosomal protein profile, in the absence of macroscopic tumour, could have four explanations: (i) exosomes are released from remaining undetected tumour cells, (ii) the exosomes are released from non-malignant tissue that has been altered through signalling from the tumour, (iii) the exosomes are altered by the scarring process after TUR-B, or (iv) that a normal bladder urothelium releases these exosomes. Due to the malignant profile of these exosomes, we find the last two alternatives less likely. Therefore, we suggest that these exosomes are derived from transformed cells in the bladder. Furthermore, these exosomes may be potentiating local tumour dissemination through rewiring metabolic networks in healthy tissue, where a pre-metastatic niche is established, thus favouring establishment of metastases.

Specifically, exosomes from bladder urine were shown to overexpress proteins involved in glycolysis and gluconeogenesis, both relevant to cancer metabolism. Cancer cells are known to undergo a glycolytic shift, leading to the production of macromolecules required for the higher nutrient demand^21^. This contributes to an acidic extracellular environment, promoting metastasis, drug resistance and immune suppression^22^.

Moreover, our finding that the pentose phosphate pathway was enriched in exosomes derived from the bladder urine is of interest, seeing as shuttling of carbon into the pentose phosphate pathway has been shown important for the ability of cancer cells to withstand oxidative stress and provide building blocks for sustained replication^23^. Up-regulation of glutathione metabolism, also enriched in bladder urine exosomes, is another mechanism for cancer cells to combat oxidative stress, and glutathione transferase activity has previously been associated with bladder cancer progression^24^. Further supporting our findings, metabolomic studies have identified glycolysis, glutathione and phenylalanine as among main pathways dysregulated in bladder cancer^25^. Phenylalanine as well as lactate, a marker of active glycolysis, have also been found to be overexpressed in several studies of bladder cancer^26^. These findings, together with the down-regulation of SLC4A1, a pH-regulating membrane protein, in exosomes from the bladder urine support the fact that metabolic dysregulation is a major function of these exosomes.

Even though this study was not primarily designed to find or validate biomarkers, several potential biomarkers, such as TPP1, TMPRSS2 and FOLR1, were detected and highly upregulated in urinary exosomes derived from the bladder compared to those derived from the ureter. The TMPRSS2:ERG fusion protein has been discussed as a prognostic marker for prostate cancer in urine and tissue^27^ and was described to be overexpressed in prostate cells and shed in prostasomes^28^, however this has not previously been associated with UBC. FOLR1 has been shown to be up-regulated in several cancers, including lung and ovarian, and clinical trials with targeted antibodies are underway in these cancer types^29,30^. In addition, our finding of FOLR1 by western blot in urinary exosomes but not in whole urine further encourages larger studies on exosomal FOLR1 as biomarkers. TPP1 is a serine protease also known as CLN2, mainly known for being mutated in certain neurodegenerative diseases, but it has also been found to have increased activity in squamous esophageal carcinoma^31^ and breast cancer^32^, and is overexpressed and associated with liver metastasis in colorectal cancer^33^.

Taken together, TPP1, TMPRSS2 and FOLR1 could all be possible prognostic markers and treatment targets in bladder cancer. However, the potential biomarkers identified in this study needs to be evaluated in larger cohorts with differential staging and longer follow-up. Interestingly, both EPSL1 and EPSL2, which were among the proteins we found to be overexpressed in bladder exosomes, have been previously described as overexpressed in urinary microvesicles of bladder cancer patients, compared to healthy controls^34^. Additionally, NRAS, EHD4, ITGB1 and MUC1, which were among the protein set correlating with bladder cancer on PCA, have been found in various studies of bladder cancer exosomes^35,36^. This further supports our hypothesis that the exosomes found in our study are malignant; i.e., despite macroscopic tumour ablation, exosomes carrying a malignant metabolic phenotype are present in the bladder. Our findings further support that urinary exosomes are a good source of biomarkers in urinary bladder cancer. While we cannot draw definite conclusions on how these exosomes impact recurrence in this study, other studies comparing partial with radical cystectomy support our hypothesis that the bladder and surrounding tissues are susceptible to recurrence followed by metastasis as long as the bladder is present. In line with this, partial cystectomy, i.e. local resection with macroscopic and microscopic free margins, shows higher recurrence rates, than RC^37^.

In conclusion, we demonstrate that urinary exosomes from the bladder, even when no macroscopic tumour remain after a combination of TUR-B and NAC, differ from exosomes found in urine from the upper tract. These exosomes are showing a malignant metabolic phenotype, which could promote metastasis and recurrence. Through pathway analysis, we provide support for exosomal involvement in establishing a pre-metastatic niche through rewiring of metabolic signalling networks. We suggest that the bladder acts as a reservoir for exosomes able to disseminate to regional and distant sites in lymph nodes and distant organs, where they aid dissemination through metabolic rewiring. We hypothesise that this explains why so many invasive bladder cancer patients relapse even after NAC and RC, and the even higher recurrence rates in non-muscle invasive bladder cancer patients not treated with RC. Further follow-up, including metabolomic profiling of urine and bladder tissue is needed to establish this as a fact. In our proposed model, exosomes retain a malignant memory phenotype in the bladder even after TUR-B plus NAC, emphasising the importance of radical over minor surgery to remove the source of tumour-promoting exosomes.

## Supplementary information

Supplementary Figures.

Supplementary Dataset.

## Data Availability

All datasets generated during the study are available on request from the corresponding author.
