# Supplementary material for: Urinary Exosomes from Bladder Cancer Patients Show a Residual Cancer Phenotype despite Complete Pathological Downstaging
Source: Sci Rep. 2020 Apr 6;10:5960. doi: 10.1038/s41598-020-62753-x (PMC7136268; doi:10.1038/s41598-020-62753-x)
Supplement: Supplementary file 1 — Supplementary Figures. [file 41598_2020_62753_MOESM1_ESM.docx]

**Title:**

**Urinary Exosomes from Bladder Cancer Patients Show a Residual Cancer Phenotype despite Complete Pathological Downstaging**

**Running Title:** Malignant Exosomes in Downstaged Bladder Cancer

**Authors:**

Stefanie Hiltbrunner^1^*, Michael Mints^1,7^*, Maria Eldh^1^, Robert Rosenblatt^2,7^, Benny Holmström^3^, Farhood Alamdari^4^, Markus Johansson^5^, Rosanne E. Veerman^1^, Ola Winqvist^6^, Amir Sherif^7^, Susanne Gabrielsson^1^†

**Affiliations:**

^1^Division of Immunology and Allergy, Department of Medicine Solna, Karolinska Institute, Stockholm, Sweden

^2^Department of Urology, Södersjukhuset, Stockholm, Sweden

^3^Department of Urology, Akademiska University Hospital, Uppsala, Sweden

^4^Department of Urology, Västmanland Hospital, Västerås, Sweden

^5^Department of Urology, Sundsvall Hospital, Sundsvall, Umeå University, Sweden

^6^Department of Clinical Immunology and Transfusion Medicine, Karolinska University Hospital, Solna, Sweden

^7^Department of Surgical and Perioperative Sciences, Urology and Andrology, Umeå University, Umeå, Sweden

* These authors contributed equally to this work

**Supplementary Figures:**


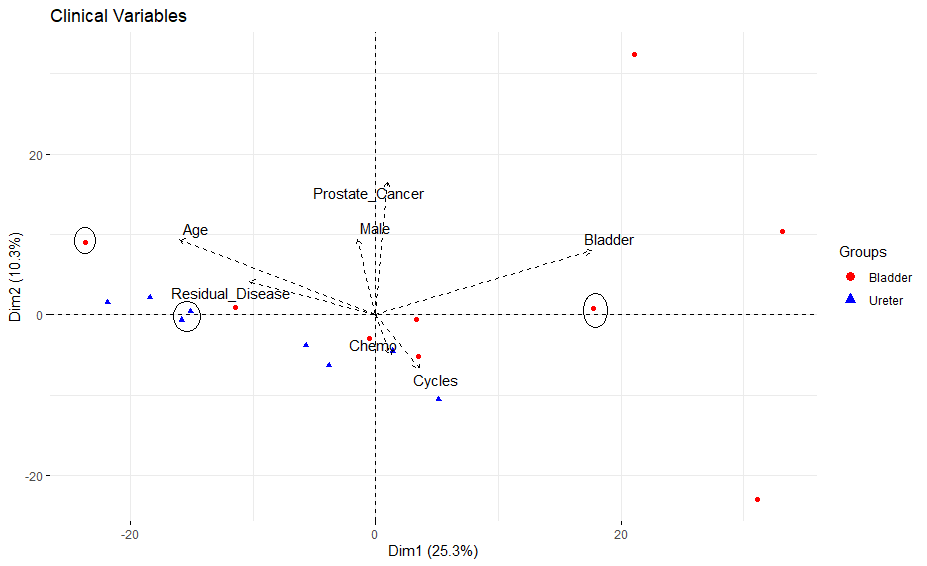


**Figure S1.** PCA biplot of clinical variables and their relationship to the PCA axes. Dots represent samples, dots with a circle represents samples from patients with residual tumor left. Dashed lines represent clinical variables – the longer the line, the stronger the contribution of that variable to separating the samples in the two first principal components.


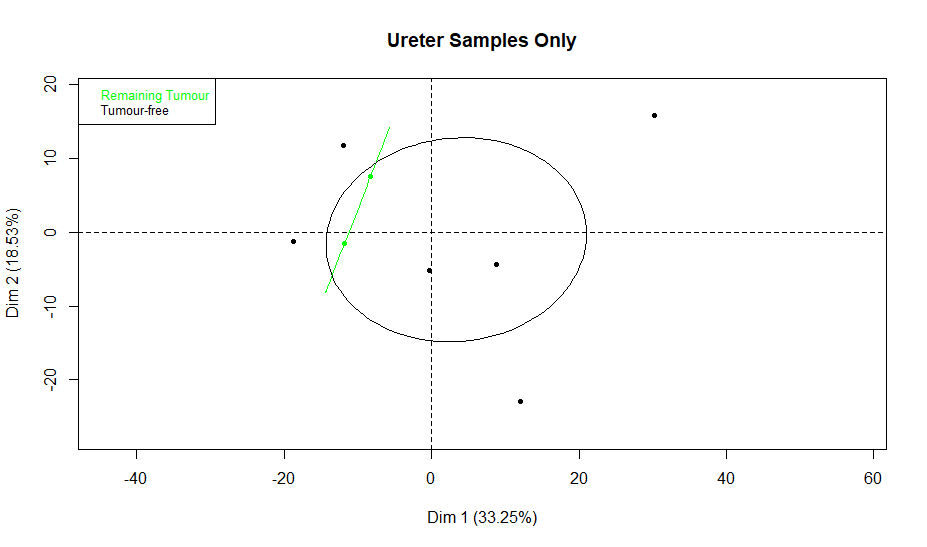


**Figure S2**. PCA of ureter samples only, with confidence ellipses for samples from tumour-free patients and patients with remaining tumour in the bladder, respectively.


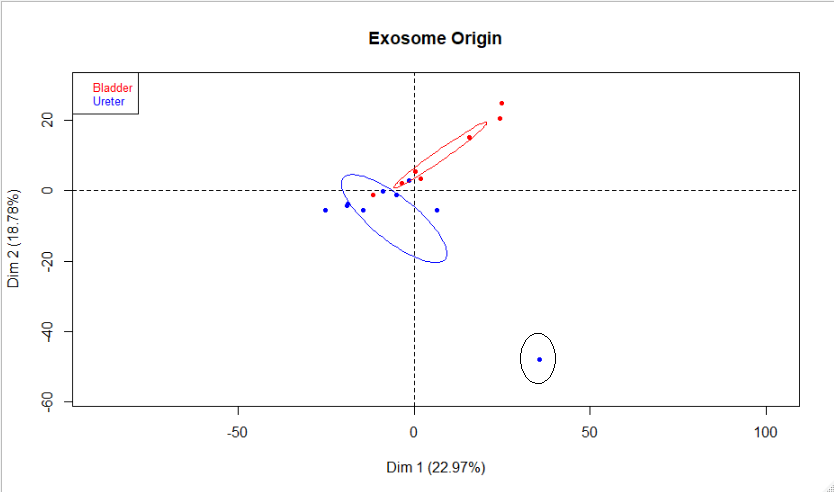


**Figure S3**. PCA of all samples showing the circled sample to be an outlier.
