# Supplementary material for: Urinary Exosomes from Bladder Cancer Patients Show a Residual Cancer Phenotype despite Complete Pathological Downstaging
Source: Sci Rep. 2020 Apr 6;10:5960. doi: 10.1038/s41598-020-62753-x (PMC7136268; doi:10.1038/s41598-020-62753-x)
Supplement: Supplementary file 2 — Supplementary Dataset. [file 41598_2020_62753_MOESM2_ESM.pdf]

Title:
Urinary Exosomes from Bladder Cancer Patients Show a Residual Cancer Phenotype despite Complete Pathological Downstaging

Running Title: Malignant Exosomes in Downstaged Bladder Cancer

Authors:
Stefanie Hiltbrunner[1]*, Michael Mints[1,7]*, Maria Eldh[1], Robert Rosenblatt[2,7], Benny Holmström[3], Farhood Alamdari[4], Markus Johansson[5], Rosanne E. Veerman[1], Ola Winqvist[6], Amir Sherif[7], Susanne Gabrielsson[1]†

Affiliations:
[1]Division of Immunology and Allergy, Department of Medicine Solna, Karolinska Institute, Stockholm, Sweden
[2]Department of Urology, Södersjukhuset, Stockholm, Sweden
[3]Department of Urology, Akademiska University Hospital, Uppsala, Sweden
[4]Department of Urology, Västmanland Hospital, Västerås, Sweden
[5]Department of Urology, Sundsvall Hospital, Sundsvall, Umeå University, Sweden
[6]Department of Clinical Immunology and Transfusion Medicine, Karolinska University Hospital, Solna, Sweden
[7]Department of Surgical and Perioperative Sciences, Urology and Andrology, Umeå University, Umeå, Sweden

* These authors contributed equally to this work

Table S1. Proteomics data.

uniprotswissprot	hgnc_symbol	Wikigene description	correlation with PCA 1st dim	p.value
A0PJK1	SLC5A10	solute carrier family 5 member 10	0.6813155	5.161365e-03
A5D8V6	VPS37C	VPS37C, ESCRT-I subunit	0.7299169	2.006751e-03
A6NEC2	NPEPPSL1	Puromycin-sensitive aminopeptidase-like protein	0.6430716	9.707747e-03
O00159	MYO1C	myosin IC	0.7405192	1.591065e-03
O00161	SNAP23	synaptosome associated protein 23	0.6077654	1.624171e-02
O00194	RAB27B	RAB27B, member RAS oncogene family	0.7838011	5.444528e-04
O00299	CLIC1	chloride intracellular channel 1	0.7103114	3.002814e-03
O00322	UPK1A	uroplakin 1A	0.6427640	9.753951e-03
O00401	WASL	Wiskott-Aldrich syndrome like	0.6833499	4.978398e-03
O00526	UPK2	uroplakin 2	0.6046419	1.695148e-02
O00560	SDCBP	syndecan binding protein	0.6527124	8.344162e-03
O00592	PODXL	podocalyxin like	0.6783757	5.435127e-03
O14745	SLC9A3R1	SLC9A3 regulator 1	0.5520500	3.286478e-02
O14773	TPP1	tripeptidyl peptidase 1	0.5379587	3.859049e-02
O15162	PLSCR1	phospholipid scramblase 1	0.6805678	5.229936e-03
O15244	SLC22A2	solute carrier family 22 member 2	0.5312251	4.157517e-02
O15393	TMPRSS2	transmembrane protease, serine 2	0.6822678	5.075068e-03
O15400	STX7	syntaxin 7	0.7368612	1.725790e-03
O15484	CAPN5	calpain 5	0.6997702	3.682076e-03
O43175	PHGDH	phosphoglycerate dehydrogenase	0.7904837	4.517279e-04
O43280	TREH	trehalase	0.5312251	4.157517e-02
O43451	MGAM	maltase-glucoamylase	0.6071541	1.637878e-02
O43488	AKR7A2	aldo-keto reductase family 7 member A2	0.8125730	2.318302e-04
O43490	PROM1	prominin 1	0.6108534	1.556272e-02
O43511	SLC26A4	solute carrier family 26 member 4	0.6238668	1.293856e-02
O43633	CHMP2A	charged multivesicular body protein 2A	0.6620188	7.174934e-03
O43707	ACTN4	actinin alpha 4	0.7505258	1.265245e-03
O43795	MYO1B	myosin IB	0.6912910	4.312740e-03
O43895	XPNPEP2	X-prolyl aminopeptidase 2	0.7288424	2.053329e-03
O60361	NME2P1	Putative nucleoside diphosphate kinase	0.7571369	1.081264e-03
O60500	NPHS1	NPHS1, nephrin	0.5312251	4.157517e-02
O60635	TSPAN1	tetraspanin 1	0.6517634	8.471306e-03
O60658	PDE8A	phosphodiesterase 8A	0.7579858	1.059297e-03
O60701	UGDH	UDP-glucose 6-dehydrogenase	0.6336171	1.120818e-02
O60749	SNX2	sorting nexin 2	0.5312251	4.157517e-02
O60884	DNAJA2	DnaJ heat shock protein family (Hsp40) member A2	0.5903983	2.049542e-02
O75083	WDR1	WD repeat domain 1	0.7679717	8.269206e-04
O75110	ATP9A	ATPase phospholipid transporting 9A (putative)	0.6315263	1.156311e-02
O75131	CPNE3	copine 3	0.6964272	3.921293e-03
O75264	SMIM24	small integral membrane protein 24	0.6124049	1.522995e-02
O75340	PDCD6	programmed cell death 6	0.7508307	1.256239e-03
O75347	TBCA	tubulin folding cofactor A	0.5723329	2.577426e-02
O75348	ATP6V1G1	ATPase H+ transporting V1 subunit G1	0.8577622	4.328972e-05
O75351	VPS4B	vacuolar protein sorting 4 homolog B	0.7123992	2.881015e-03
O75368	SH3BGRL	SH3 domain binding glutamate rich protein like	0.7572654	1.077914e-03
O75874	IDH1	isocitrate dehydrogenase (NADP(+)) 1, cytosolic	0.8024943	3.174984e-04
O75936	BBOX1	gamma-butyrobetaine hydroxylase 1	0.6582230	7.635144e-03
O75955	FLOT1	flotillin 1	0.5279114	4.310536e-02
O94760	DDAH1	dimethylarginine dimethylaminohydrolase 1	0.7971930	3.720125e-04
O94832	MYO1D	myosin ID	0.7998311	3.440022e-04
O94856	NFASC	neurofascin	0.5312251	4.157517e-02
O95154	AKR7A3	aldo-keto reductase family 7 member A3	0.5312251	4.157517e-02
O95336	PGLS	6-phosphogluconolactonase	0.8640556	3.277386e-05
O95394	PGM3	phosphoglucomutase 3	0.6100130	1.574529e-02
O95398	RAPGEF3	Rap guanine nucleotide exchange factor 3	0.5787042	2.380687e-02
O95497	VNN1	vanin 1	0.6447800	9.454244e-03
O95630	STAMBP	STAM binding protein	0.7160881	2.675481e-03
O95716	RAB3D	RAB3D, member RAS oncogene family	0.8429352	7.943086e-05
O95865	DDAH2	dimethylarginine dimethylaminohydrolase 2	0.6123114	1.524985e-02
O96009	NAPSA	napsin A aspartic peptidase	0.7455292	1.420417e-03
P00338	LDHA	lactate dehydrogenase A	0.6396118	1.023739e-02
P00352	ALDH1A1	aldehyde dehydrogenase 1 family member A1	0.6341674	1.111619e-02
P00390	GSR	glutathione-disulfide reductase	0.7584971	1.046243e-03
P00441	SOD1	superoxide dismutase 1	0.6637493	6.972508e-03
P00558	PGK1	phosphoglycerate kinase 1	0.7709341	7.665884e-04
P00918	CA2	carbonic anhydrase 2	0.7355730	1.775362e-03
P00966	ASS1	argininosuccinate synthase 1	0.7764087	6.645004e-04
P01111	NRAS	NRAS proto-oncogene, GTPase	0.5780351	2.400794e-02
P01116	KRAS	KRAS proto-oncogene, GTPase	0.7869730	4.986817e-04
P01620	IGKV3-20	Immunoglobulin kappa variable 3-20	0.5312251	4.157517e-02
P01911	HLA-DRB1	major histocompatibility complex, class II, DR beta 1	0.6269776	1.236544e-02
P04075	ALDOA	aldolase, fructose-bisphosphate A	0.7320158	1.918219e-03
P04080	CSTB	cystatin B	0.7342562	1.827209e-03
P04216	THY1	Thy-1 cell surface antigen	0.7794424	6.128745e-04
P04406	GAPDH	glyceraldehyde-3-phosphate dehydrogenase	0.5788295	2.376934e-02
P04792	HSPB1	heat shock protein family B (small) member 1	0.8243568	1.566752e-04
P04899	GNAI2	G protein subunit alpha i2	0.6077378	1.624788e-02
P05023	ATP1A1	ATPase Na+/K+ transporting subunit alpha 1	0.6466583	9.181525e-03
P05026	ATP1B1	ATPase Na+/K+ transporting subunit beta 1	0.6229110	1.311872e-02
P05062	ALDOB	aldolase, fructose-bisphosphate B	0.7301554	1.996530e-03
P05090	APOD	apolipoprotein D	0.6144849	1.479249e-02
P05413	FABP3	fatty acid binding protein 3	0.7556298	1.121167e-03
P05556	ITGB1	integrin subunit beta 1	0.5504130	3.349495e-02
P05937	CALB1	calbindin 1	0.5348726	3.993786e-02
P05981	HPN	hepsin	0.5918491	2.011057e-02
P06733	ENO1	enolase 1	0.7036208	3.421086e-03
P06744	GPI	glucose-6-phosphate isomerase	0.7246826	2.241890e-03
P07108	DBI	diazepam binding inhibitor, acyl-CoA binding protein	0.8250754	1.528367e-04
P07148	FABP1	fatty acid binding protein 1	0.7576114	1.068940e-03
P07195	LDHB	lactate dehydrogenase B	0.6527125	8.344153e-03
P07288	KLK3	kallikrein related peptidase 3	0.7765982	6.611757e-04

P07355	ANXA2	annexin A2	0.6103655	1.566851e-02
P07384	CAPN1	calpain 1	0.6006601	1.789062e-02
P07437	TUBB	tubulin beta class I	0.6394867	1.025695e-02
P07737	PFN1	profilin 1	0.6544641	8.113415e-03
P07858	CTSB	cathepsin B	0.5767787	2.438897e-02
P07900	HSP90AA1	heat shock protein 90 alpha family class A member 1	0.7318221	1.926256e-03
P07947	YES1	YES proto-oncogene 1, Src family tyrosine kinase	0.7974594	3.691024e-04
P07948	LYN	LYN proto-oncogene, Src family tyrosine kinase	0.5837598	2.232879e-02
P08107	HSPA1A	Heat shock 70 kDa protein 1A	0.6445474	9.488451e-03
P08118	MSMB	microseminoprotein beta	0.6146154	1.476538e-02
P08133	ANXA6	annexin A6	0.5901198	2.056993e-02
P08134	RHOC	ras homolog family member C	0.6656847	6.751462e-03
P08174	CD55	CD55 molecule (Cromer blood group)	0.7513676	1.240506e-03
P08183	ABCB1	ATP binding cassette subfamily B member 1	0.7582974	1.051326e-03
P08195	SLC3A2	solute carrier family 3 member 2	0.7072848	3.186647e-03
P08238	HSP90AB1	heat shock protein 90 alpha family class B member 1	0.6490650	8.841135e-03
P08473	MME	membrane metalloendopeptidase	0.9285336	5.887613e-07
P08582	MELTF	melanotransferrin	0.7742697	7.029830e-04
P08754	GNAI3	G protein subunit alpha i3	0.7632922	9.300279e-04
P08758	ANXA5	annexin A5	0.7383555	1.669688e-03
P08F94	PKHD1	PKHD1, fibrocystin/polyductin	0.5312251	4.157517e-02
P09211	GSTP1	glutathione S-transferase pi 1	0.6828767	5.020489e-03
P09417	QDPR	quinoid dihydropteridine reductase	0.6386556	1.038768e-02
P09467	FBP1	fructose-bisphosphatase 1	0.7328731	1.882975e-03
P09525	ANXA4	annexin A4	0.5541168	3.208191e-02
P09543	CNP	2',3'-cyclic nucleotide 3' phosphodiesterase	0.8616616	3.649114e-05
P09619	PDGFRB	platelet derived growth factor receptor beta	0.5312251	4.157517e-02
P09936	UCHL1	ubiquitin C-terminal hydrolase L1	0.7568737	1.088149e-03
P09960	LTA4H	leukotriene A4 hydrolase	0.6342926	1.109534e-02
P0CG48	UBC	ubiquitin C	0.6918130	4.271597e-03
P10301	RRAS	related RAS viral (r-ras) oncogene homolog	0.8264149	1.458866e-04
P10314	HLA-A	major histocompatibility complex, class I, A	0.5312251	4.157517e-02
P10599	TXN	thioredoxin	0.5567387	3.110906e-02
P10768	ESD	esterase D	0.8330111	1.153383e-04
P11142	HSPA8	heat shock protein family A (Hsp70) member 8	0.6998998	3.673037e-03
P11234	RALB	RAS like proto-oncogene B	0.6624592	7.122990e-03
P11279	LAMP1	lysosomal associated membrane protein 1	0.6719080	6.077840e-03
P11586	MTHFD1	methylenetetrahydrofolate dehydrogenase, cyclohydrolase and formyltetrahydrofolate synthetase 1	0.7578124	1.063754e-03
P11597	CETP	cholesteryl ester transfer protein	0.5312251	4.157517e-02
P11766	ADH5	alcohol dehydrogenase 5 (class III), chi polypeptide	0.8482807	6.428844e-05
P12277	CKB	creatine kinase B	0.8150064	2.142876e-04
P12821	ACE	angiotensin I converting enzyme	0.7025235	3.493910e-03
P12931	SRC	SRC proto-oncogene, non-receptor tyrosine kinase	0.7686729	8.123045e-04
P13473	LAMP2	lysosomal associated membrane protein 2	0.7287664	2.056654e-03
P13489	RNH1	ribonuclease/angiogenin inhibitor 1	0.6353535	1.091991e-02
P13639	EEF2	eukaryotic translation elongation factor 2	0.7654583	8.810790e-04
P13796	LCP1	lymphocyte cytosolic protein 1	0.6328120	1.134383e-02
P13861	PRKAR2A	protein kinase cAMP-dependent type II regulatory subunit alpha	0.8196817	1.836333e-04
P13987	CD59	CD59 molecule (CD59 blood group)	0.5745969	2.506154e-02
P14174	MIF	macrophage migration inhibitory factor (glycosylation-inhibiting factor)	0.7706614	7.719888e-04
P14384	CPM	carboxypeptidase M	0.7567199	1.092189e-03
P14550	AKR1A1	aldo-keto reductase family 1 member A1	0.7871173	4.966766e-04
P14618	PKM	pyruvate kinase, muscle	0.5812945	2.304054e-02
P15121	AKR1B1	aldo-keto reductase family 1 member B	0.7580307	1.058146e-03
P15144	ANPEP	alanyl aminopeptidase, membrane	0.6269996	1.236146e-02
P15309	ACPP	acid phosphatase, prostate	0.7872483	4.948619e-04
P15311	EZR	ezrin	0.5708741	2.624157e-02
P15313	ATP6V1B1	ATPase H+ transporting V1 subunit B1	0.7933500	4.161234e-04
P15328	FOLR1	folate receptor 1	0.6930500	4.175339e-03
P15941	MUC1	mucin 1, cell surface associated	0.6580231	7.660006e-03
P16152	CBR1	carbonyl reductase 1	0.8476304	6.599188e-05
P16444	DPEP1	dipeptidase 1 (renal)	0.7858941	5.138846e-04
P16870	CPE	carboxypeptidase E	0.5799846	2.342568e-02
P17174	GOT1	glutamic-oxaloacetic transaminase 1	0.8295990	1.304033e-04
P17342	NPR3	natriuretic peptide receptor 3	0.6414565	9.952257e-03
P17927	CR1	complement C3b/C4b receptor 1 (Knops blood group)	0.7164160	2.657794e-03
P17931	LGALS3	galectin 3	0.5210705	4.639594e-02
P18206	VCL	vinculin	0.7246021	2.245674e-03
P18669	PGAM1	phosphoglycerate mutase 1	0.7786689	6.257160e-04
P19440	GGT1	gamma-glutamyltransferase 1	0.5161813	4.885886e-02
P20073	ANXA7	annexin A7	0.6022008	1.752262e-02
P20337	RAB3B	RAB3B, member RAS oncogene family	0.6310472	1.164565e-02
P20339	RAB5A	RAB5A, member RAS oncogene family	0.8203854	1.793487e-04
P20711	DDC	dopa decarboxylase	0.7070001	3.204387e-03
P21266	GSTM3	glutathione S-transferase mu 3	0.7549374	1.139896e-03
P21281	ATP6V1B2	ATPase H+ transporting V1 subunit B2	0.8242394	1.573103e-04
P21283	ATP6V1C1	ATPase H+ transporting V1 subunit C1	0.5312251	4.157517e-02
P21695	GPD1	glycerol-3-phosphate dehydrogenase 1	0.6715741	6.112575e-03
P21796	VDAC1	voltage dependent anion channel 1	0.6173686	1.420211e-02
P21926	CD9	CD9 molecule	0.6869093	4.670660e-03
P21964	COMT	catechol-O-methyltransferase	0.7978465	3.649064e-04
P22314	UBA1	ubiquitin like modifier activating enzyme 1	0.8196888	1.835899e-04
P22748	CA4	carbonic anhydrase 4	0.8145132	2.177525e-04
P23526	AHCY	adenosylhomocysteinase	0.7879975	4.845878e-04
P23528	CFL1	cofilin 1	0.7376335	1.696606e-03
P24666	ACP1	acid phosphatase 1, soluble	0.5312251	4.157517e-02
P25311	AZGP1	alpha-2-glycoprotein 1, zinc-binding	0.6073166	1.634226e-02
P26012	ITGB8	integrin subunit beta 8	0.5312251	4.157517e-02
P26038	MSN	moesin	0.7515223	1.236002e-03
P27105	STOM	stomatin	0.7032975	3.442415e-03
P27348	YWHAQ	tyrosine 3-monooxygenase/tryptophan 5-monooxygenase activation protein theta	0.8609235	3.770514e-05
P27487	DPP4	dipeptidyl peptidase 4	0.7285436	2.066431e-03
P28838	LAP3	leucine aminopeptidase 3	0.8033286	3.095484e-04
P28907	CD38	CD38 molecule	0.6280722	1.216854e-02

P29401	TKT	transketolase	0.5504782	3.346969e-02
P29972	AQP1	aquaporin 1 (Colton blood group)	0.6041817	1.705804e-02
P29992	GNA11	G protein subunit alpha 11	0.6546993	8.082812e-03
P30039	PBLD	phenazine biosynthesis like protein domain containing	0.7578213	1.063526e-03
P30041	PRDX6	peroxiredoxin 6	0.7170728	2.622645e-03
P30043	BLVRB	biliverdin reductase B	0.6611565	7.277516e-03
P30044	PRDX5	peroxiredoxin 5	0.7015189	3.561654e-03
P30046	DDT	D-dopachrome tautomerase	0.6418687	9.889413e-03
P30085	CMPK1	cytidine/uridine monophosphate kinase 1	0.8409954	8.560478e-05
P30086	PEBP1	phosphatidylethanolamine binding protein 1	0.8264325	1.457973e-04
P30153	PPP2R1A	protein phosphatase 2 scaffold subunit Aalpha	0.5887379	2.094276e-02
P30492	HLA-B	major histocompatibility complex, class I, B	0.5312251	4.157517e-02
P30626	SRI	sorcin	0.6185559	1.396442e-02
P31150	GDI1	GDP dissociation inhibitor 1	0.6414016	9.960655e-03
P31639	SLC5A2	solute carrier family 5 member 2	0.6251634	1.269724e-02
P31689	DNAJA1	DnaJ heat shock protein family (Hsp40) member A1	0.7579453	1.060337e-03
P31939	ATIC	5-aminoimidazole-4-carboxamide ribonucleotide formyltransferase/IMP cyclohydrolase	0.6055050	1.675301e-02
P31946	YWHAB	tyrosine 3-monooxygenase/tryptophan 5-monooxygenase activation protein beta	0.7486300	1.322421e-03
P31947	SFN	stratifin	0.7140528	2.787374e-03
P31949	S100A11	S100 calcium binding protein A11	0.6234257	1.302147e-02
P32119	PRDX2	peroxiredoxin 2	0.6151513	1.465440e-02
P33176	KIF5B	kinesin family member 5B	0.8381528	9.536259e-05
P34896	SHMT1	serine hydroxymethyltransferase 1	0.7861942	5.096182e-04
P35080	PFN2	profilin 2	0.7459472	1.406878e-03
P35237	SERPINB6	serpin family B member 6	0.6330413	1.130506e-02
P35241	RDX	radixin	0.6643283	6.905795e-03
P35558	PCK1	phosphoenolpyruvate carboxykinase 1	0.5312251	4.157517e-02
P35579	MYH9	myosin heavy chain 9	0.5380501	3.855112e-02
P36405	ARL3	ADP ribosylation factor like GTPase 3	0.7580195	1.058434e-03
P36543	ATP6V1E1	ATPase H+ transporting V1 subunit E1	0.7560275	1.110525e-03
P36969	GPX4	glutathione peroxidase 4	0.6422967	9.824470e-03
P38606	ATP6V1A	ATPase H+ transporting V1 subunit A	0.8265506	1.451972e-04
P40121	CAPG	capping actin protein, gelsolin like	0.6555520	7.972639e-03
P40925	MDH1	malate dehydrogenase 1	0.8354092	1.056319e-04
P41181	AQP2	aquaporin 2	0.7429375	1.506747e-03
P41743	PRKCI	protein kinase C iota	0.5312251	4.157517e-02
P42685	FRK	fyn related Src family tyrosine kinase	0.6968035	3.893760e-03
P43353	ALDH3B1	aldehyde dehydrogenase 3 family member B1	0.6888110	4.512533e-03
P45974	USP5	ubiquitin specific peptidase 5	0.5312251	4.157517e-02
P46108	CRK	CRK proto-oncogene, adaptor protein	0.5312251	4.157517e-02
P46940	IQGAP1	IQ motif containing GTPase activating protein 1	0.6402983	1.013055e-02
P47755	CAPZA2	capping actin protein of muscle Z-line alpha subunit 2	0.8551260	4.845431e-05
P47756	CAPZB	capping actin protein of muscle Z-line beta subunit	0.7542760	1.158019e-03
P48509	CD151	CD151 molecule (Raph blood group)	0.5399988	3.771860e-02
P48637	GSS	glutathione synthetase	0.6413970	9.961369e-03
P49189	ALDH9A1	aldehyde dehydrogenase 9 family member A1	0.6219747	1.329710e-02
P49221	TGM4	transglutaminase 4	0.5312251	4.157517e-02
P49327	FASN	fatty acid synthase	0.6042380	1.704498e-02
P49773	HINT1	histidine triad nucleotide binding protein 1	0.8431720	7.870304e-05
P50053	KHK	ketohexokinase	0.6671517	6.587623e-03
P50148	GNAQ	G protein subunit alpha q	0.7636890	9.208997e-04
P50395	GDI2	GDP dissociation inhibitor 2	0.7944337	4.032745e-04
P50502	ST13	ST13, Hsp70 interacting protein	0.8304185	1.266454e-04
P50895	BCAM	basal cell adhesion molecule (Lutheran blood group)	0.8173223	1.986181e-04
P50990	CCT8	chaperonin containing TCP1 subunit 8	0.6074277	1.631731e-02
P50995	ANXA11	annexin A11	0.7214444	2.398083e-03
P51148	RAB5C	RAB5C, member RAS oncogene family	0.7426462	1.516708e-03
P51149	RAB7A	RAB7A, member RAS oncogene family	0.6803918	5.246178e-03
P51153	RAB13	RAB13, member RAS oncogene family	0.7965135	3.795211e-04
P51159	RAB27A	RAB27A, member RAS oncogene family	0.5952922	1.921934e-02
P51178	PLCD1	phospholipase C delta 1	0.6441985	9.539937e-03
P52209	PGD	phosphogluconate dehydrogenase	0.8199248	1.821439e-04
P52565	ARHGDIA	Rho GDP dissociation inhibitor alpha	0.8441273	7.582225e-05
P52788	SMS	spermine synthase	0.6223838	1.321893e-02
P53396	ACLY	ATP citrate lyase	0.5934429	1.969417e-02
P53990	IST1	IST1, ESCRT-III associated factor	0.6048940	1.689333e-02
P54289	CACNA2D1	calcium voltage-gated channel auxiliary subunit alpha2delta 1	0.8088904	2.606026e-04
P54652	HSPA2	heat shock protein family A (Hsp70) member 2	0.7578988	1.061533e-03
P54793	ARSF	arylsulfatase F	0.6699116	6.287876e-03
P54920	NAPA	NSF attachment protein alpha	0.7349049	1.801517e-03
P55017	SLC12A3	solute carrier family 12 member 3	0.8174535	1.977595e-04
P55072	VCP	valosin containing protein	0.5923788	1.997143e-02
P58876	HIST1H2BD	histone cluster 1 H2B family member d	0.6154359	1.459573e-02
P59665	DEFA1	defensin alpha 1	0.5953013	1.921702e-02
P60033	CD81	CD81 molecule	0.5312251	4.157517e-02
P60174	TPI1	triosephosphate isomerase 1	0.7030689	3.457561e-03
P60660	MYL6	myosin light chain 6	0.5509019	3.330578e-02
P60709	ACTB	actin beta	0.6711438	6.157572e-03
P60953	CDC42	cell division cycle 42	0.6970459	3.876112e-03
P60981	DSTN	destrin, actin depolymerizing factor	0.8083100	2.653932e-04
P61006	RAB8A	RAB8A, member RAS oncogene family	0.6856828	4.774949e-03
P61019	RAB2A	RAB2A, member RAS oncogene family	0.7585737	1.044299e-03
P61020	RAB5B	RAB5B, member RAS oncogene family	0.7515445	1.235357e-03
P61026	RAB10	RAB10, member RAS oncogene family	0.5524060	3.272891e-02
P61106	RAB14	RAB14, member RAS oncogene family	0.5754525	2.479613e-02
P61158	ACTR3	ARP3 actin related protein 3 homolog	0.6200642	1.366689e-02
P61160	ACTR2	ARP2 actin related protein 2 homolog	0.5252999	4.434043e-02
P61204	ARF3	ADP ribosylation factor 3	0.7272680	2.123136e-03
P62158	CALM1	Calmodulin-1	0.5312251	4.157517e-02
P61457	PCBD1	pterin-4 alpha-carbinolamine dehydratase 1	0.6658118	6.737146e-03
P61586	RHOA	ras homolog family member A	0.7321736	1.911695e-03
P61981	YWHAG	tyrosine 3-monooxygenase/tryptophan 5-monooxygenase activation protein gamma	0.8628689	3.457523e-05
P62140	PPP1CB	protein phosphatase 1 catalytic subunit beta	0.5805605	2.325576e-02
P62258	YWHAE	tyrosine 3-monooxygenase/tryptophan 5-monooxygenase activation protein epsilon	0.8259208	1.484192e-04

P62330	ARF6	ADP ribosylation factor 6	0.5613347	2.945750e-02
P62491	RAB11A	RAB11A, member RAS oncogene family	0.6366118	1.071465e-02
P62745	RHOB	ras homolog family member B	0.6723042	6.036818e-03
P62820	RAB1A	RAB1A, member RAS oncogene family	0.7857986	5.152482e-04
P62873	GNB1	G protein subunit beta 1	0.7546059	1.148952e-03
P62879	GNB2	G protein subunit beta 2	0.6491545	8.828665e-03
P62937	PPIA	peptidylprolyl isomerase A	0.6966670	3.903734e-03
P63000	RAC1	ras-related C3 botulinum toxin substrate 1 (rho family, small GTP binding protein Rac1)	0.7257098	2.194088e-03
P63027	VAMP2	vesicle associated membrane protein 2	0.6370063	1.065092e-02
P63092	GNAS	GNAS complex locus	0.6862332	4.727920e-03
P63096	GNAI1	G protein subunit alpha i1	0.6534913	8.240938e-03
P63104	YWHAZ	tyrosine 3-monooxygenase/tryptophan 5-monooxygenase activation protein zeta	0.6507211	8.612698e-03
P68032	ACTC1	actin, alpha, cardiac muscle 1	0.6991576	3.725017e-03
P68104	EEF1A1	eukaryotic translation elongation factor 1 alpha 1	0.8402767	8.799034e-05
P68363	TUBA1B	tubulin alpha 1b	0.8165655	2.036320e-04
P68371	TUBB4B	tubulin beta 4B class IVb	0.7024042	3.501898e-03
P68402	PAFAH1B2	platelet activating factor acetylhydrolase 1b catalytic subunit 2	0.6386157	1.039397e-02
P78369	CLDN10	claudin 10	0.5312251	4.157517e-02
P78417	GSTO1	glutathione S-transferase omega 1	0.6354676	1.090117e-02
P80297	MT1X	metallothionein 1X	0.6464679	9.208897e-03
P80723	BASP1	brain abundant membrane attached signal protein 1	0.6486561	8.898253e-03
P98161	PKD1	polycystin 1, transient receptor potential channel interacting	0.5312251	4.157517e-02
P98164	LRP2	LDL receptor related protein 2	0.6395528	1.024662e-02
P98172	EFNB1	ephrin B1	0.7031143	3.454550e-03
Q00796	SORD	sorbitol dehydrogenase	0.8334476	1.135187e-04
Q01518	CAP1	cyclase associated actin cytoskeleton regulatory protein 1	0.7833330	5.514887e-04
Q03154	ACY1	aminoacylase 1	0.5987312	1.835967e-02
Q03167	TGFBR3	transforming growth factor beta receptor 3	0.5312251	4.157517e-02
Q04609	FOLH1	folate hydrolase 1	0.5752653	2.485401e-02
Q04760	GLO1	glyoxalase I	0.8576292	4.353883e-05
Q05655	PRKCD	protein kinase C delta	0.6807098	5.216861e-03
Q06830	PRDX1	peroxiredoxin 1	0.7318618	1.924608e-03
Q07075	ENPEP	glutamyl aminopeptidase	0.8171740	1.995929e-04
Q08380	LGALS3BP	galectin 3 binding protein	0.5272362	4.342219e-02
Q12765	SCRN1	secernin 1	0.6438362	9.593641e-03
Q12913	PTPRJ	protein tyrosine phosphatase, receptor type J	0.7238601	2.280771e-03
Q12929	EPS8	epidermal growth factor receptor pathway substrate 8	0.7625106	9.482201e-04
Q13018	PLA2R1	phospholipase A2 receptor 1	0.5312251	4.157517e-02
Q13057	COASY	Coenzyme A synthase	0.5312251	4.157517e-02
Q13131	PRKAA1	protein kinase AMP-activated catalytic subunit alpha 1	0.5312251	4.157517e-02
Q13228	SELENBP1	selenium binding protein 1	0.7496347	1.291865e-03
Q13277	STX3	syntaxin 3	0.7204601	2.447247e-03
Q13621	SLC12A1	solute carrier family 12 member 1	0.6872831	4.639239e-03
Q14117	DPYS	dihydropyrimidinase	0.5312251	4.157517e-02
Q14118	DAG1	dystroglycan 1	0.5312251	4.157517e-02
Q14240	EIF4A2	eukaryotic translation initiation factor 4A2	0.5746463	2.504615e-02
Q14254	FLOT2	flotillin 2	0.6298507	1.185381e-02
Q14344	GNA13	G protein subunit alpha 13	0.6079165	1.620796e-02
Q14894	CRYM	crystallin mu	0.6055641	1.673948e-02
Q14914	PTGR1	prostaglandin reductase 1	0.5777558	2.409223e-02
Q14974	KPNB1	karyopherin subunit beta 1	0.7466654	1.383853e-03
Q15181	PPA1	pyrophosphatase (inorganic) 1	0.5312251	4.157517e-02
Q15185	PTGES3	prostaglandin E synthase 3	0.6695604	6.325410e-03
Q15274	QPRT	quinolinate phosphoribosyltransferase	0.5312251	4.157517e-02
Q15286	RAB35	RAB35, member RAS oncogene family	0.7795056	6.118341e-04
Q15365	PCBP1	poly(rC) binding protein 1	0.8460647	7.024668e-05
Q15599	SLC9A3R2	SLC9A3 regulator 2	0.7938601	4.100346e-04
Q15833	STXBP2	syntaxin binding protein 2	0.8216976	1.715775e-04
Q16651	PRSS8	protease, serine 8	0.7482280	1.334810e-03
Q16775	HAGH	hydroxyacylglutathione hydrolase	0.5156835	4.911494e-02
Q16827	PTPRO	protein tyrosine phosphatase, receptor type O	0.7702838	7.795167e-04
Q3LXA3	TKFC	triokinase and FMN cyclase	0.8047852	2.960576e-04
Q3SXY8	ARL13B	ADP ribosylation factor like GTPase 13B	0.7529636	1.194673e-03
Q4KMQ2	ANO6	anoctamin 6	0.5570255	3.100400e-02
Q4U2R8	SLC22A6	solute carrier family 22 member 6	0.7578262	1.063401e-03
Q53GD3	SLC44A4	solute carrier family 44 member 4	0.6673579	6.564847e-03
Q5IJ48	CRB2	crumbs 2, cell polarity complex component	0.6219216	1.330728e-02
Q5VW32	BROX	BRO1 domain and CAAX motif containing	0.7575373	1.070858e-03
Q687X5	STEAP4	STEAP4 metalloreductase	0.6293131	1.194826e-02
Q6EMK4	VASN	vasorin	0.5312251	4.157517e-02
Q6IWH7	ANO7	anoctamin 7	0.6278687	1.220496e-02
Q6P1N0	CC2D1A	coiled-coil and C2 domain containing 1A	0.7477738	1.348918e-03
Q6UXI9	NPNT	nephronectin	0.8519903	5.525032e-05
Q6ZQN7	SLCO4C1	solute carrier organic anion transporter family member 4C1	0.6662874	6.683766e-03
Q6ZS17	RIPOR1	RHO family interacting cell polarization regulator 1	0.7535775	1.177414e-03
Q7L576	CYFIP1	cytoplasmic FMR1 interacting protein 1	0.7687717	8.102615e-04
Q7L5L3	GDPD3	glycerophosphodiester phosphodiesterase domain containing 3	0.7528602	1.197602e-03
Q7L7X3	TAOK1	TAO kinase 1	0.6858692	4.758982e-03
Q7L9L4	MOB1B	MOB kinase activator 1B	0.7577346	1.065759e-03
Q7LBR1	CHMP1B	charged multivesicular body protein 1B	0.7102973	3.003651e-03
Q7Z2W4	ZC3HAV1	zinc finger CCCH-type containing, antiviral 1	0.7342384	1.827920e-03
Q7Z404	TMC4	transmembrane channel like 4	0.6290164	1.200065e-02
Q7Z4W1	DCXR	dicarbonyl and L-xylulose reductase	0.8188132	1.890377e-04
Q86XT2	VPS37D	VPS37D, ESCRT-I subunit	0.6352396	1.093864e-02
Q86YQ8	CPNE8	copine 8	0.6200632	1.366709e-02
Q8IWA5	SLC44A2	solute carrier family 44 member 2	0.7170408	2.624351e-03
Q8IX04	UEVLD	UEV and lactate/malate dehyrogenase domains	0.5647603	2.827023e-02
Q8IY95	TMEM192	transmembrane protein 192	0.5667273	2.760503e-02
Q8IYJ3	SYTL1	synaptotagmin like 1	0.8056325	2.884348e-04
Q8IZP0	ABI1	abl interactor 1	0.7997477	3.448613e-04
Q8N271	PROM2	prominin 2	0.7358156	1.765939e-03
Q8N392	ARHGAP18	Rho GTPase activating protein 18	0.8543182	5.013507e-05
Q8N3R9	MPP5	membrane palmitoylated protein 5	0.7580059	1.058783e-03
Q8N4C8	MINK1	misshapen like kinase 1	0.7534744	1.180298e-03

Q8N5I2	ARRDC1	arrestin domain containing 1	0.7340056	1.837215e-03
Q8N9U0	TC2N	tandem C2 domains, nuclear	0.6193596	1.380525e-02
Q8NFJ5	GPRC5A	G protein-coupled receptor class C group 5 member A	0.7882099	4.817066e-04
Q8NFU3	TSTD1	thiosulfate sulfurtransferase like domain containing 1	0.7919087	4.337298e-04
Q8NI35	PATJ	PATJ, crumbs cell polarity complex component	0.6008841	1.783674e-02
Q8TAD7	C12orf75	chromosome 12 open reading frame 75	0.7577480	1.065413e-03
Q8TE68	EPS8L1	EPS8 like 1	0.8222924	1.681470e-04
Q8TF65	GIPC2	GIPC PDZ domain containing family member 2	0.5312251	4.157517e-02
Q8WTX9	ZDHHC1	zinc finger DHHC-type containing 1	0.6427897	9.750094e-03
Q8WUM4	PDCD6IP	programmed cell death 6 interacting protein	0.6975491	3.839670e-03
Q8WW52	FAM151A	family with sequence similarity 151 member A	0.6322471	1.143976e-02
Q8WV92	MITD1	microtubule interacting and trafficking domain containing 1	0.7132236	2.834019e-03
Q8WWT9	SLC13A3	solute carrier family 13 member 3	0.7057454	3.283528e-03
Q92485	SMPDL3B	sphingomyelin phosphodiesterase acid like 3B	0.7503298	1.271063e-03
Q92597	NDRG1	N-myc downstream regulated 1	0.8280906	1.375606e-04
Q92630	DYRK2	dual specificity tyrosine phosphorylation regulated kinase 2	0.5312251	4.157517e-02
Q92817	EVPL	envoplakin	0.5973227	1.870812e-02
Q92930	RAB8B	RAB8B, member RAS oncogene family	0.7165059	2.652962e-03
Q93050	ATP6V0A1	ATPase H+ transporting V0 subunit a1	0.6737639	5.887581e-03
Q93088	BHMT	betaine--homocysteine S-methyltransferase	0.7200818	2.466359e-03
Q969P0	IGSF8	immunoglobulin superfamily member 8	0.6795266	5.326617e-03
Q96A22	C11orf52	chromosome 11 open reading frame 52	0.7163988	2.658716e-03
Q96C24	SYTL4	synaptotagmin like 4	0.6154803	1.458661e-02
Q96CF2	CHMP4C	charged multivesicular body protein 4C	0.5930685	1.979137e-02
Q96DG6	CMBL	carboxymethylenebutenolidase homolog	0.7547878	1.143974e-03
Q96EY5	MVB12A	multivesicular body subunit 12A	0.8505696	5.857883e-05
Q96FN5	KIF12	kinesin family member 12	0.7305110	1.981368e-03
Q96FZ7	CHMP6	charged multivesicular body protein 6	0.6740048	5.863235e-03
Q96IU4	ABHD14B	abhydrolase domain containing 14B	0.7706915	7.713906e-04
Q96J02	ITCH	itchy E3 ubiquitin protein ligase	0.8173835	1.982172e-04
Q96KP4	CNDP2	carnosine dipeptidase 2	0.8625033	3.514638e-05
Q96LD4	TRIM47	tripartite motif containing 47	0.5566670	3.113535e-02
Q96RF0	SNX18	sorting nexin 18	0.8416676	8.342192e-05
Q99497	PARK7	Parkinsonism associated deglycase	0.7682318	8.214753e-04
Q99536	VAT1	vesicle amine transport 1	0.6837784	4.940526e-03
Q99571	P2RX4	purinergic receptor P2X 4	0.5191256	4.736442e-02
Q99653	CHP1	calcineurin like EF-hand protein 1	0.5312251	4.157517e-02
Q99816	TSG101	tumor susceptibility 101	0.7347180	1.808891e-03
Q99835	SMO	smoothened, frizzled class receptor	0.6564191	7.861825e-03
Q99961	SH3GL1	SH3 domain containing GRB2 like 1, endophilin A2	0.6232758	1.304975e-02
Q9BRA2	TXNDC17	thioredoxin domain containing 17	0.5312251	4.157517e-02
Q9BRK3	MXRA8	matrix remodeling associated 8	0.5280628	4.303456e-02
Q9BUL8	PDCD10	programmed cell death 10	0.8122620	2.341548e-04
Q9BUT1	BDH2	3-hydroxybutyrate dehydrogenase 2	0.7071702	3.193776e-03
Q9BW04	C1orf116	chromosome 1 open reading frame 116	0.8012553	3.296134e-04
Q9BW30	TPPP3	tubulin polymerization promoting protein family member 3	0.5312251	4.157517e-02
Q9BV36	MLPH	melanophilin	0.6001312	1.801830e-02
Q9BWD1	ACAT2	acetyl-CoA acetyltransferase 2	0.6349492	1.098650e-02
Q9BY43	CHMP4A	charged multivesicular body protein 4A	0.6461121	9.260196e-03
Q9BZQ8	FAM129A	family with sequence similarity 129 member A	0.5953632	1.920127e-02
Q9BZV1	UBXN6	UBX domain protein 6	0.7486682	1.321247e-03
Q9C0H2	TTYH3	tweety family member 3	0.7274559	2.114704e-03
Q9H0E2	TOLLIP	toll interacting protein	0.8099745	2.518452e-04
Q9H0U4	RAB1B	RAB1B, member RAS oncogene family	0.6076439	1.626889e-02
Q9H0W9	C11orf54	chromosome 11 open reading frame 54	0.5140312	4.997197e-02
Q9H190	SDCBP2	syndecan binding protein 2	0.7546845	1.146797e-03
Q9H223	EHD4	EH domain containing 4	0.7521081	1.219067e-03
Q9H2G2	SLK	STE20 like kinase	0.7544909	1.152104e-03
Q9H2K8	TAOK3	TAO kinase 3	0.7980326	3.629034e-04
Q9H2M3	BHMT2	betaine--homocysteine S-methyltransferase 2	0.5312251	4.157517e-02
Q9H3R2	MUC13	mucin 13, cell surface associated	0.5142486	4.985858e-02
Q9H444	CHMP4B	charged multivesicular body protein 4B	0.6998507	3.676461e-03
Q9H4A4	RNPEP	arginyl aminopeptidase	0.6270489	1.235253e-02
Q9H4G4	GLIPR2	GLI pathogenesis related 2	0.5669941	2.751574e-02
Q9H4M9	EHD1	EH domain containing 1	0.7731921	7.230426e-04
Q9H6S3	EPS8L2	EPS8 like 2	0.8493002	6.169101e-05
Q9H7P6	MVB12B	multivesicular body subunit 12B	0.7537673	1.172119e-03
Q9H9H4	VPS37B	VPS37B, ESCRT-I subunit	0.8275696	1.401059e-04
Q9HBG4	ATP6V0A4	ATPase H+ transporting V0 subunit a4	0.8544925	4.976840e-05
Q9HD42	CHMP1A	charged multivesicular body protein 1A	0.7859410	5.132167e-04
Q9NP72	RAB18	RAB18, member RAS oncogene family	0.5312251	4.157517e-02
Q9NP79	VTA1	vesicle trafficking 1	0.7057699	3.281970e-03
Q9NP85	NPHS2	NPHS2, podocin	0.8037726	3.053838e-04
Q9NQ84	GPRC5C	G protein-coupled receptor class C group 5 member C	0.6878041	4.595717e-03
Q9NQA5	TRPV5	transient receptor potential cation channel subfamily V member 5	0.5153649	4.927933e-02
Q9NQR4	NIT2	nitrilase family member 2	0.6460307	9.271964e-03
Q9NQX5	NPDC1	neural proliferation, differentiation and control 1	0.6219562	1.330065e-02
Q9NR45	NANS	N-acetylneuraminate synthase	0.6331003	1.129511e-02
Q9NRM0	SLC2A9	solute carrier family 2 member 9	0.5312251	4.157517e-02
Q9NRX4	PHPT1	phosphohistidine phosphatase 1	0.7070293	3.202566e-03
Q9NV96	TMEM30A	transmembrane protein 30A	0.6710070	6.171934e-03
Q9NXU5	ARL15	ADP ribosylation factor like GTPase 15	0.7579678	1.059759e-03
Q9NZH0	GPRC5B	G protein-coupled receptor class C group 5 member B	0.8237343	1.600645e-04
Q9NZM1	MYOF	myoferlin	0.6283602	1.211714e-02
Q9NZV1	CRIM1	cysteine rich transmembrane BMP regulator 1	0.6449164	9.434229e-03
Q9NZZ3	CHMP5	charged multivesicular body protein 5	0.7534739	1.180312e-03
Q9UBI6	GNG12	G protein subunit gamma 12	0.6892681	4.475172e-03
Q9UBP0	SPAST	spastin	0.5916640	2.015935e-02
Q9UBQ7	GRHPR	glyoxylate and hydroxypyruvate reductase	0.6445110	9.493813e-03
Q9UBV8	PEF1	penta-EF-hand domain containing 1	0.8211932	1.745315e-04
Q9UGT4	SUSD2	sushi domain containing 2	0.6524971	8.372878e-03
Q9UHI7	SLC23A1	solute carrier family 23 member 1	0.5770118	2.431793e-02
Q9UHR4	BAIAP2L1	BAI1 associated protein 2 like 1	0.7642971	9.070527e-04
Q9UI12	ATP6V1H	ATPase H+ transporting V1 subunit H	0.8506465	5.839453e-05

Q9UK41	VPS28	VPS28, ESCRT-I subunit	0.7728825	7.288927e-04
Q9UKS6	PACSIN3	protein kinase C and casein kinase substrate in neurons 3	0.7763139	6.661695e-04
Q9UL25	RAB21	RAB21, member RAS oncogene family	0.8599533	3.935161e-05
Q9UM54	MYO6	myosin VI	0.8626769	3.487420e-05
Q9UN37	VPS4A	vacuolar protein sorting 4 homolog A	0.7133796	2.825199e-03
Q9UNF0	PACSIN2	protein kinase C and casein kinase substrate in neurons 2	0.8276081	1.399168e-04
Q9UQB8	BAIAP2	BAI1 associated protein 2	0.6900803	4.409384e-03
Q9UQN3	CHMP2B	charged multivesicular body protein 2B	0.7200607	2.467430e-03
Q9Y287	ITM2B	integral membrane protein 2B	0.5827482	2.261879e-02
Q9Y2S2	CRYL1	crystallin lambda 1	0.8645666	3.202250e-05
Q9Y3E7	CHMP3	charged multivesicular body protein 3	0.8635852	3.347841e-05
Q9Y3R5	DOPEY2	dopey family member 2	0.6225360	1.318996e-02
Q9Y490	TLN1	talin 1	0.7267653	2.145820e-03
Q9Y5K6	CD2AP	CD2 associated protein	0.8205721	1.782255e-04
Q9Y617	PSAT1	phosphoserine aminotransferase 1	0.7516651	1.231856e-03
Q9Y696	CLIC4	chloride intracellular channel 4	0.6026654	1.741277e-02
Q9Y6E0	STK24	serine/threonine kinase 24	0.7465390	1.387884e-03
Q9Y6R1	SLC4A4	solute carrier family 4 member 4	0.7530033	1.193552e-03
Q9Y6W3	CAPN7	calpain 7	0.7303593	1.987824e-03

Table S2. Differential expression for proteins across clinical parameters.

Uniprot Accession	HGNC Symbol	Wikigene description	Prostate_Cancer_logfc	Prostate_Cancer_p.value	Chemo_logfc	Chemo_p.value	Male_logfc	Male_p.value	Bladder_logfc	Bladder_p.value
P02730	SLC4A1	solute carrier family 4 member 1 (Diego blood group)	0.612732087	0.9189448	-0.722842498	9.18E-01	0.817275998	0.796476479	-5.091758941	5.61E-06
P02042	HBD	hemoglobin subunit delta	1.195040643	0.852021	-1.360279828	8.38E-01	2.271687232	0.49858242	-3.839714599	1.70E-01
P69905	HBA2	hemoglobin subunit alpha 2	0.488602273	0.9225917	2.059619008	7.39E-01	2.219094798	0.49858242	-3.512491284	7.88E-02
P09758	TACSTD2	tumor associated calcium signal transducer 2	-1.600353864	0.606544	-0.306280501	9.55E-01	0.257804023	0.945693298	-3.40024048	7.19E-02
P68871	HBB	hemoglobin subunit beta	1.198022089	0.6401388	1.623879366	7.94E-01	2.76344534	0.49858242	-2.902783114	1.10E-01
P04040	CAT	catalase	-0.254074277	0.9487033	-2.670520343	5.97E-01	1.574567072	0.49858242	-2.5737639	1.70E-01
P08729	KRT7	keratin 7	-0.926277947	0.7243624	-1.610869373	5.97E-01	0.35476929	0.889206745	-2.550785708	8.99E-02
P35221	CTNNA1	catenin alpha 1	-2.251525564	0.606544	1.84215728	5.97E-01	-1.501194481	0.523405303	-2.53296626	1.10E-01
P35222	CTNNB1	catenin beta 1	-2.137693012	0.606544	0.434518426	9.04E-01	-1.258509531	0.575800847	-2.404904638	1.02E-01
P00915	CA1	carbonic anhydrase 1	0.126053147	0.9790738	-0.963778127	8.42E-01	0.181984413	0.952344606	-2.224960479	1.95E-01
P11166	SLC2A1	solute carrier family 2 member 1	0.011002635	0.9953954	-0.790142272	8.72E-01	0.206858763	0.941538111	-2.135757355	1.87E-01
P16070	CD44	CD44 molecule (Indian blood group)	0.728306474	0.8089368	-0.674844316	8.41E-01	1.201111033	0.49858242	-2.069083625	1.70E-01
P08603	CFH	complement factor H	1.061250551	0.8184847	1.39881098	8.09E-01	-0.24546098	0.957265005	-1.824850373	4.05E-01
O60716	CTNND1	catenin delta 1	-1.583280464	0.606544	0.172588957	9.55E-01	-0.5297679	0.838926923	-1.781190522	2.09E-01
P08727	KRT19	keratin 19	-1.710299463	0.606544	-0.754104901	8.38E-01	-0.929940869	0.738171102	-1.559256613	4.05E-01
P00734	F2	coagulation factor II, thrombin	-0.180025397	0.9677089	0.548505413	9.04E-01	-0.420833901	0.878195	-1.510458907	4.05E-01
P16157	ANK1	ankyrin 1	0.799431182	0.8340657	-1.520980825	7.45E-01	1.187693624	0.49858242	-1.48461703	3.33E-01
P16452	EPB42	erythrocyte membrane protein band 4.2	0.622629268	0.8721792	-1.293553246	7.78E-01	1.164718118	0.49858242	-1.455897648	3.15E-01
P50897	PPT1	palmitoyl-protein thioesterase 1	-1.257982333	0.606544	1.029258273	5.97E-01	-0.676768968	0.778956299	-1.415230125	3.10E-01
P16403	HIST1H1C	histone cluster 1 H1 family member c	0.242887067	0.9357694	-0.899649501	8.17E-01	1.054009594	0.49858242	-1.317511992	3.10E-01
O60814	HIST1H2BK	histone cluster 1 H2B family member k	1.224163005	0.7503981	-2.499730146	5.97E-01	2.077277866	0.49858242	-1.30679313	4.66E-01
P28066	PSMA5	proteasome subunit alpha 5	-1.15810131	0.606544	0.947537435	5.97E-01	-0.497617792	0.817499764	-1.302863974	3.10E-01
P14923	JUP	junction plakoglobin	-1.127064184	0.606544	0.922143424	5.97E-01	-0.761850227	0.734141735	-1.267947208	3.15E-01
P11277	SPTB	spectrin beta, erythrocytic	1.662968798	0.606544	-1.113578147	7.78E-01	0.997781279	0.49858242	-1.247226599	3.15E-01
Q9Y624	F11R	F11 receptor	-1.095483411	0.606544	-0.527253662	8.59E-01	-0.719138861	0.738171102	-1.232418837	3.15E-01
P30101	PDIA3	protein disulfide isomerase family A member 3	0.043187511	0.9886331	-1.061652448	7.84E-01	0.980896666	0.49858242	-1.226120833	3.15E-01
P02647	APOA1	apolipoprotein A1	0.482165542	0.9189448	2.421850438	5.97E-01	1.559081797	0.532173149	-1.218075234	4.61E-01
P35542	SAA4	serum amyloid A4, constitutive	-0.91337394	0.6870404	1.694204837	5.97E-01	0.430974269	0.843844089	-1.211062736	4.05E-01
Q9P2B2	PTGFRN	prostaglandin F2 receptor inhibitor	-1.042358398	0.606544	0.852838689	5.97E-01	-0.520100103	0.793741327	-1.172653198	3.10E-01
Q04828	AKR1C1	aldo-keto reductase family 1 member C1	0.193833086	0.9371571	-0.659589601	8.26E-01	-0.532858499	0.785396207	-1.159684738	3.10E-01
Q14764	MVP	major vault protein	-0.579368236	0.9086495	1.289444291	7.78E-01	1.719830799	0.49858242	-1.155442981	5.05E-01
P25786	PSMA1	proteasome subunit alpha 1	-0.981965894	0.606544	-0.643215189	8.26E-01	0.883769305	0.49858242	-1.104711631	3.10E-01
P08514	ITGA2B	integrin subunit alpha 2b	-0.96470322	0.606544	0.789302635	5.97E-01	-0.200421642	0.909075327	-1.085291123	3.15E-01
P02749	APOH	apolipoprotein H	0.171644066	0.9371571	0.789071766	5.97E-01	-0.509007563	0.78099142	-1.084973678	3.10E-01
Q9HCH5	SYTL2	synaptotagmin like 2	-0.962579584	0.606544	0.787565114	5.97E-01	-1.732643251	0.49858242	-1.082902032	3.15E-01
P07738	BPGM	bisphosphoglycerate mutase	0.479445012	0.8604831	-0.979950801	7.74E-01	-0.171311569	0.923322861	-1.076737785	3.16E-01
P01765	NA	NA	0.535746366	0.9041836	0.222839601	9.55E-01	0.404010308	0.862480694	-1.060246106	4.47E-01
P25788	PSMA3	proteasome subunit alpha 3	-0.926853872	0.606544	0.758334987	5.97E-01	-0.416945289	0.807619793	-1.042710607	3.10E-01
P13716	ALAD	aminolevulinate dehydratase	0.301133082	0.9189448	-0.747822189	8.17E-01	0.832149495	0.49858242	-1.040186869	3.10E-01
Q5QNW6	HIST2H2BF	histone cluster 2 H2B family member f	1.627184358	0.6738958	-0.67412293	9.20E-01	2.350587285	0.49858242	-1.036039189	6.18E-01
P05106	ITGB3	integrin subunit beta 3	0.443627381	0.9189448	-1.407060146	6.23E-01	0.47158376	0.837774529	-1.009754865	4.57E-01
P01889	HLA-B	major histocompatibility complex, class I, B	0.333471787	0.9124383	-0.775282337	8.08E-01	0.805247318	0.49858242	-1.006559147	3.11E-01
Q14651	PLS1	plastin 1	-0.75877658	0.7435884	0.142672514	9.62E-01	-2.207332493	0.49858242	-0.948592148	4.40E-01
P69891	HBG1	hemoglobin subunit gamma 1	-0.813267541	0.606544	0.665400716	5.97E-01	0.731940787	0.49858242	-0.914925984	4.05E-01
P02743	APCS	amyloid P component, serum	-2.071593982	0.606544	0.100499978	9.76E-01	-1.046873023	0.629743838	-0.89321571	5.12E-01
P62826	RAN	RAN, member RAS oncogene family	0.638470767	0.8271667	-0.113487993	9.75E-01	0.560758112	0.736277848	-0.892609716	4.28E-01
Q9BQE3	TUBA1C	tubulin alpha 1c	0.305176385	0.9225917	0.148587898	9.56E-01	1.627972111	0.496346254	-0.877853021	4.53E-01
P05546	SERPIND1	serpin family D member 1	1.227554454	0.7268602	1.380029945	6.37E-01	0.206508591	0.953177657	-0.782907935	6.80E-01
P00738	HP	haptoglobin	-1.721676311	0.606544	2.419312552	5.97E-01	-0.21124506	0.911322336	-0.770914875	6.17E-01
P16401	HIST1H1B	histone cluster 1 H1 family member b	1.034526734	0.6983379	-0.528250987	9.14E-01	1.582149741	0.49858242	-0.763137593	5.81E-01
O76070	SNCG	synuclein gamma	-1.822710343	0.606544	-0.753517081	8.41E-01	-3.063270841	0.317975793	-0.760092302	6.17E-01
P61224	RAP1B	RAP1B, member of RAS oncogene family	1.390476218	0.6256201	-1.953170626	5.97E-01	-0.316031461	0.917193947	-0.720812202	6.91E-01
P0DJI8	SAA1	serum amyloid A1	-0.6201585	0.606544	-1.395356625	5.97E-01	0.55814265	0.49858242	-0.697678312	4.05E-01
P18463	HLA-B	major histocompatibility complex, class I, B	-0.611087527	0.606544	0.499980704	5.97E-01	-1.099957548	0.49858242	-0.687473468	4.05E-01
O43520	ATP8B1	ATPase phospholipid transporting 8B1	-0.603241131	0.606544	0.493560925	5.97E-01	-1.085834035	0.49858242	-0.678646272	4.05E-01
Q13885	TUBB2A	tubulin beta 2A class IIa	-0.601308886	0.606544	0.491979997	5.97E-01	0.541177997	0.49858242	-0.676472496	4.05E-01
P02549	SPTA1	spectrin alpha, erythrocytic 1	0.881730999	0.606544	-1.322596499	5.97E-01	0.5290386	0.49858242	-0.66129825	4.05E-01
P30443	HLA-A	major histocompatibility complex, class I, A	-0.57693346	0.606544	-1.298100285	5.97E-01	0.519240114	0.49858242	-0.649050143	4.05E-01
P10319	HLA-B	major histocompatibility complex, class I, B	-0.573470758	0.606544	-1.290309206	5.97E-01	0.516123683	0.49858242	-0.645154603	4.05E-01
P28074	PSMB5	proteasome subunit beta 5	-0.572883003	0.606544	0.468722457	5.97E-01	0.515594703	0.49858242	-0.644493378	4.05E-01
P11171	EPB41	erythrocyte membrane protein band 4.1	0.856172161	0.606544	-1.284258242	5.97E-01	0.513703297	0.49858242	-0.642129121	4.05E-01
P01605	NA	NA	0.854360656	0.606544	0.466014904	5.97E-01	0.512616394	0.49858242	-0.640770492	4.05E-01
P10451	SPP1	secreted phosphoprotein 1	0.853437513	0.606544	0.465511371	5.97E-01	0.512062508	0.49858242	-0.640078135	4.05E-01
P62136	PPP1CA	protein phosphatase 1 catalytic subunit alpha	-0.562700271	0.606544	0.46039113	5.97E-01	-1.012860487	0.49858242	-0.633037804	4.05E-01
P07360	C8G	complement C8 gamma chain	0.838678429	0.606544	0.457460961	5.97E-01	0.503207058	0.49858242	-0.629008822	4.05E-01
P18564	ITGB6	integrin subunit beta 6	-0.557541743	0.606544	0.456170517	5.97E-01	-1.003575137	0.49858242	-0.627234461	4.05E-01
A6NMY6	NA	NA	-0.549917358	0.606544	0.449932384	5.97E-01	0.494925622	0.49858242	-0.618657028	4.05E-01
B0FP48	UPK3BL1	uroplakin 3B like 1	-0.549554456	0.606544	0.449635464	5.97E-01	-0.98919802	0.49858242	-0.618248763	4.05E-01
P04632	CAPNS1	calpain small subunit 1	0.821895936	0.606544	-1.232843903	5.97E-01	0.493137561	0.49858242	-0.616421952	4.05E-01
P60763	RAC3	Rac family small GTPase 3	-0.545719747	0.606544	0.446497975	5.97E-01	0.491147772	0.49858242	-0.613934715	4.05E-01
P02724	GYPA	glycophorin A (MNS blood group)	0.804812263	0.606544	-1.207218395	5.97E-01	0.482887358	0.49858242	-0.603609198	4.05E-01
P13798	APEH	acylaminoacyl-peptide hydrolase	0.786624682	0.606544	-1.179937023	5.97E-01	0.471974809	0.49858242	-0.589968511	4.05E-01
Q14126	DSG2	desmoglein 2	-0.521698342	0.606544	0.426844098	5.97E-01	-0.939057015	0.49858242	-0.586910634	4.05E-01
P01023	A2M	alpha-2-macroglobulin	-1.152042789	0.7305966	3.300071961	5.97E-01	-0.363275313	0.878266067	-0.586343954	7.46E-01
Q9NUN5	LMBRD1	LMBR1 domain containing 1	-0.519607452	0.606544	0.42513337	5.97E-01	0.467646707	0.49858242	-0.584558384	4.05E-01
Q9UHN6	CEMIP2	cell migration inducing hyaluronidase 2	-0.513962095	0.606544	0.420514441	5.97E-01	-0.925131771	0.49858242	-0.578207357	4.05E-01
P30464	HLA-B	major histocompatibility complex, class I, B	-0.323162086	0.9225917	-0.396734428	9.21E-01	0.000111244	0.999924593	-0.573896638	6.67E-01
P26447	S100A4	S100 calcium binding protein A4	0.762554891	0.606544	-1.143832336	5.97E-01	0.457532934	0.49858242	-0.571916168	4.05E-01
P11021	HSPA5	heat shock protein family A (Hsp70) member 5	-0.508175146	0.606544	-1.143394079	5.97E-01	0.457357631	0.49858242	-0.571697039	4.05E-01
P00568	AK1	adenylate kinase 1	0.7597147	0.606544	-1.13957205	5.97E-01	0.45582882	0.49858242	-0.569786025	4.05E-01
P23229	ITGA6	integrin subunit alpha 6	-0.506453175	0.606544	0.41437078	5.97E-01	-0.911615716	0.49858242	-0.569759822	4.05E-01
P37840	SNCA	synuclein alpha	0.757162597	0.606544	-1.135743896	5.97E-01	0.454297558	0.49858242	-0.567871948	4.05E-01
P18462	HLA-A	major histocompatibility complex, class I, A	0.756558474	0.606544	0.412668259	5.97E-01	0.453935085	0.49858242	-0.567418856	4.05E-01
O95833	CLIC3	chloride intracellular channel 3	-0.504063344	0.606544	0.412415463	5.97E-01	-0.907314019	0.49858242	-0.567071262	4.05E-01
O75631	UPK3A	uroplakin 3A	-1.703766263	0.606544	1.393990579	5.97E-01	-3.066779273	0.496346254	-0.565975179	6.89E-01
P15259	PGAM2	phosphoglycerate mutase 2	0.753902947	0.606544	-1.130854421	5.97E-01	0.452341768	0.49858242	-0.565427211	4.05E-01
P29692	EEF1D	eukaryotic translation elongation factor 1 delta	-0.500851956	0.606544	0.409787964	5.97E-01	-0.901533521	0.49858242	-0.563458451	4.05E-01
O00151	PDLIM1	PDZ and LIM domain 1	-0.500778437	0.606544	0.409727812	5.97E-01	-0.901401187	0.49858242	-0.563375742	4.05E-01
P04350	TUBB4A	tubulin beta 4A class IVa	-0.497608059	0.606544	-1.119618133	5.97E-01	0.447847253	0.49858242	-0.559809067	4.05E-01
P18124	RPL7	ribosomal protein L7	-0.49155644	0.606544	-1.106001989	5.97E-01	0.442400796	0.49858242	-0.553000995	4.05E-01
P16144	ITGB4	integrin subunit beta 4	-0.491089333	0.606544	0.401800364	5.97E-01	-0.8839608	0.49858242	-0.5524755	4.05E-01
Q99829	CPNE1	copine 1	-0.489649021	0.606544	0.400621926	5.97E-01	-0.881368238	0.49858242	-0.550855149	4.05E-01
P60900	PSMA6	proteasome subunit alpha 6	-0.305740076	0.9225917	1.302636288	5.97E-01	0.105949343	0.967847875	-0.550695773	6.73E-01
Q9H4G0	EPB41L1	erythrocyte membrane protein band 4.1 like 1	-0.48945918	0.606544	0.400466602	5.97E-01	-0.881026525	0.49858242	-0.550641578	4.05E-01
Q99436	PSMB7	proteasome subunit beta 7	-0.488532454	0.606544	0.399708372	5.97E-01	0.439679209	0.49858242	-0.549599011	4.05E-01
Q86X29	LSR	lipolysis stimulated lipoprotein receptor	-0.486919623	0.606544	0.398388782	5.97E-01	-0.876455321	0.49858242	-0.547784575	4.05E-01
P00533	EGFR	epidermal growth factor receptor	-0.480797154	0.606544	0.393379489	5.97E-01	-0.865434877	0.49858242	-0.540896798	4.05E-01
Q02878	RPL6	ribosomal protein L6	-0.479750527	0.606544	-1.079438686	5.97E-01	0.431775475	0.49858242	-0.539719343	4.05E-01
Q3YBM2	TMEM176B	transmembrane protein 176B	-0.477566822	0.606544	0.390736491	5.97E-01	0.42981014	0.49858242	-0.537262675	4.05E-01
P36578	RPL4	ribosomal protein L4	-0.473537161	0.606544	-1.065458612	5.97E-01	0.426183445	0.49858242	-0.532729306	4.05E-01
O75355	ENTPD3	ectonucleoside triphosphate diphosphohydrolase 3	-0.472830095	0.606544	0.386860987	5.97E-01	-0.851094171	0.49858242	-0.531933857	4.05E-01
P43652	AFM	afamin	-0.469615748	0.606544	0.384231067	5.97E-01	0.422654173	0.49858242	-0.528317717	4.05E-01
O14818	PSMA7	proteasome subunit alpha 7	-0.468260817	0.606544	0.383122487	5.97E-01	0.421434736	0.49858242	-0.526793419	4.05E-01
O15031	PLXNB2	plexin B2	-0.301631941	0.9225917	-1.471523548	5.97E-01	-0.284151625	0.90439469	-0.525843352	6.65E-01
P62888	RPL30	ribosomal protein L30	-0.466539413	0.606544	-1.049713679	5.97E-01	0.419885472	0.49858242	-0.52485684	4.05E-01
P49755	TMED10	transmembrane p24 trafficking protein 10	-0.463358308	0.606544	-1.042556193	5.97E-01	0.417022477	0.49858242	-0.521278097	4.05E-01
Q9UHD8	SEPTIN9	septin 9	-0.462980028	0.606544	0.378801841	5.97E-01	-0.83336405	0.49858242	-0.520852531	4.05E-01
P28072	PSMB6	proteasome subunit beta 6	0.618519857	0.7649939	-0.150303803	9.55E-01	1.114835003	0.49858242	-0.517054379	6.00E-01
P01764	IGHV3-23	NA	0.689274407	0.606544	0.375967858	5.97E-01	0.413564644	0.49858242	-0.516955805	4.05E-01
P52895	AKR1C2	aldo-keto reductase family 1 member C2	-0.458319307	0.606544	0.374988524	5.97E-01	-0.824974754	0.49858242	-0.515609221	4.05E-01
P01781	NA	NA	1.080756831	0.606544	-0.15861923	9.55E-01	1.3068023	0.49858242	-0.515445858	6.65E-01
P13727	PRG2	proteoglycan 2, pro eosinophil major basic protein	0.682973075	0.606544	0.372530768	5.97E-01	0.409783845	0.49858242	-0.512229806	4.05E-01
Q96QK1	VPS35	VPS35 retromer complex component	-0.455296928	0.606544	0.372515668	5.97E-01	-0.819534471	0.49858242	-0.512209044	4.05E-01
P17301	ITGA2	integrin subunit alpha 2	-0.451504628	0.606544	0.369412878	5.97E-01	-0.812708331	0.49858242	-0.507942707	4.05E-01
O75369	FLNB	filamin B	-0.450236968	0.606544	0.368375701	5.97E-01	-0.810426543	0.49858242	-0.506516589	4.05E-01
P15880	RPS2	ribosomal protein S2	-0.449149919	0.606544	-1.010587317	5.97E-01	0.404234927	0.49858242	-0.505293658	4.05E-01
P06239	LCK	LCK proto-oncogene, Src family tyrosine kinase	-0.446344831	0.606544	-1.00427587	5.97E-01	0.401710348	0.49858242	-0.502137935	4.05E-01
P00736	NA	NA	0.667372915	0.606544	0.36402159	5.97E-01	0.400423749	0.49858242	-0.500529686	4.05E-01
P35573	AGL	amylo-alpha-1, 6-glucosidase, 4-alpha-glucanotransferase	-0.444162943	0.606544	0.363406044	5.97E-01	-0.799493297	0.49858242	-0.499683311	4.05E-01
Q14019	COTL1	coactosin like F-actin binding protein 1	0.665569774	0.606544	-0.998354661	5.97E-01	0.399341864	0.49858242	-0.499177331	4.05E-01
P30050	RPL12	ribosomal protein L12	-0.442041376	0.606544	-0.994593096	5.97E-01	0.397837239	0.49858242	-0.497296548	4.05E-01
P78310	CXADR	CXADR Ig-like cell adhesion molecule	-0.44070242	0.606544	0.360574707	5.97E-01	-0.793264356	0.49858242	-0.495790222	4.05E-01
P04626	ERBB2	erb-b2 receptor tyrosine kinase 2	-0.438941577	0.606544	0.359134017	5.97E-01	-0.790094838	0.49858242	-0.493809274	4.05E-01
P06681	C2	complement C2	0.656930301	0.606544	-0.985395452	5.97E-01	0.394158181	0.49858242	-0.492697726	4.05E-01
O95994	AGR2	anterior gradient 2, protein disulphide isomerase family mem	-0.433574963	0.606544	-0.975543667	5.97E-01	0.390217467	0.49858242	-0.487771834	4.05E-01
P01602	IGKV1-5	NA	-0.433260328	0.606544	0.354485723	5.97E-01	0.389934295	0.49858242	-0.487417869	4.05E-01

P01766	IGHV3-13	NA	0.070669707	0.9886331	0.641932146	9.00E-01	1.101846767	0.651775557	-0.486492666	8.13E-01
Q9Y5Y6	ST14	suppression of tumorigenicity 14	-0.430825729	0.606544	0.352493778	5.97E-01	-0.775486312	0.49858242	-0.484678945	4.05E-01
P20020	ATP2B1	ATPase plasma membrane Ca2+ transporting 1	-0.42913359	0.606544	0.351109301	5.97E-01	-0.772440462	0.49858242	-0.482775289	4.05E-01
P41218	MNDA	myeloid cell nuclear differentiation antigen	0.981246303	0.606544	-0.171036343	9.55E-01	1.210257989	0.49858242	-0.47994194	6.64E-01
Q92734	TFG	trafficking from ER to golgi regulator	-0.426558013	0.606544	0.34900201	5.97E-01	-0.767804423	0.49858242	-0.479877764	4.05E-01
P16422	EPCAM	epithelial cell adhesion molecule	-0.229241601	0.9357694	-0.570810679	8.38E-01	0.357155899	0.865894662	-0.474502226	7.05E-01
P25787	PSMA2	proteasome subunit alpha 2	-0.419730768	0.606544	0.343416083	5.97E-01	0.377757691	0.49858242	-0.472197114	4.05E-01
Q12846	STX4	syntaxin 4	-0.419629041	0.606544	0.343332852	5.97E-01	-0.755332274	0.49858242	-0.472082671	4.05E-01
Q92599	SEPTIN8	septin 8	-0.418349534	0.606544	0.342285983	5.97E-01	-0.753029162	0.49858242	-0.470643226	4.05E-01
Q15366	PCBP2	poly(rC) binding protein 2	0.626711575	0.606544	0.341842677	5.97E-01	0.376026945	0.49858242	-0.470033681	4.05E-01
O15195	VILL	villin like	-0.41679442	0.606544	0.341013616	5.97E-01	-0.750229956	0.49858242	-0.468893723	4.05E-01
O15231	ZNF185	zinc finger protein 185 with LIM domain	-0.415137781	0.606544	0.339658184	5.97E-01	-0.747248005	0.49858242	-0.467030003	4.05E-01
P27635	RPL10	ribosomal protein L10	-0.415084782	0.606544	-0.93394076	5.97E-01	0.373576304	0.49858242	-0.46697038	4.05E-01
Q15102	PAFAH1B3	platelet activating factor acetylhydrolase 1b catalytic subunit	0.617499806	0.606544	-0.926249709	5.97E-01	0.370499884	0.49858242	-0.463124855	4.05E-01
Q9BYE9	CDHR2	cadherin related family member 2	-0.410180631	0.606544	0.335602335	5.97E-01	-0.738325136	0.49858242	-0.46145321	4.05E-01
O14936	CASK	calcium/calmodulin dependent serine protein kinase	-0.408674584	0.606544	0.334370114	5.97E-01	-0.735614251	0.49858242	-0.459758907	4.05E-01
P28070	PSMB4	proteasome subunit beta 4	-0.407517313	0.606544	0.333423256	5.97E-01	0.366765582	0.49858242	-0.458456977	4.05E-01
Q9Y5Z4	HEBP2	heme binding protein 2	-0.402199699	0.606544	0.329072481	5.97E-01	-0.723959459	0.49858242	-0.452474662	4.05E-01
P07237	P4HB	prolyl 4-hydroxylase subunit beta	-0.401393136	0.606544	-0.903134555	5.97E-01	0.361253822	0.49858242	-0.451567278	4.05E-01
P62424	RPL7A	ribosomal protein L7a	-0.400972601	0.606544	-0.902188352	5.97E-01	0.360875341	0.49858242	-0.451094176	4.05E-01
Q8IVW8	SPNS2	sphingolipid transporter 2	-0.390238031	0.606544	0.319285661	5.97E-01	0.351214228	0.49858242	-0.439017785	4.05E-01
P20160	AZU1	azurocidin 1	0.975327813	0.8121217	-0.040896617	9.95E-01	1.08934306	0.567502564	-0.437779868	8.22E-01
P21589	NT5E	5'-nucleotidase ecto	-0.386427748	0.606544	0.316168158	5.97E-01	-0.695569947	0.49858242	-0.434731217	4.05E-01
Q92530	PSMF1	proteasome inhibitor subunit 1	0.573189204	0.606544	-0.859783805	5.97E-01	0.343913522	0.49858242	-0.429891903	4.05E-01
Q00013	MPP1	membrane palmitoylated protein 1	0.569402391	0.606544	-0.854103587	5.97E-01	0.341641435	0.49858242	-0.427051793	4.05E-01
P18283	GPX2	glutathione peroxidase 2	-0.371035775	0.606544	0.303574725	5.97E-01	-0.667864396	0.49858242	-0.417415247	4.05E-01
P04843	RPN1	ribophorin I	-0.369408472	0.606544	-0.831169061	5.97E-01	0.332467624	0.49858242	-0.415584531	4.05E-01
P12956	XRCC6	X-ray repair cross complementing 6	-0.34751526	0.606544	-0.781909336	5.97E-01	0.312763734	0.49858242	-0.390954668	4.05E-01
Q8WWI5	SLC44A1	solute carrier family 44 member 1	-0.342739831	0.606544	0.280423498	5.97E-01	0.308465848	0.49858242	-0.38558231	4.05E-01
O60493	SNX3	sorting nexin 3	-0.340843079	0.606544	0.27887161	5.97E-01	-0.613517542	0.49858242	-0.383448464	4.05E-01
P26006	ITGA3	integrin subunit alpha 3	0.358875892	0.930027	-3.742553751	5.41E-05	-0.962435446	0.682498634	-0.3574878	8.45E-01
P80188	LCN2	lipocalin 2	1.508455196	0.606544	0.00919759	9.97E-01	1.621037732	0.49858242	-0.354998418	8.34E-01
Q14210	LY6D	lymphocyte antigen 6 family member D	-1.154469954	0.606544	-1.312839613	6.87E-01	-1.611555459	0.49858242	-0.354081249	8.27E-01
P01008	SERPINC1	serpin family C member 1	1.446143651	0.606544	0.188911893	9.55E-01	0.565753912	0.812285999	-0.327643557	8.50E-01
P27918	CFP	complement factor properdin	-0.348365001	0.9189448	1.20109285	5.97E-01	-0.165101992	0.9483529	-0.324445412	8.12E-01
P05155	SERPING1	serpin family G member 1	1.205487282	0.606544	0.513389082	8.55E-01	0.853253425	0.582720693	-0.316786024	8.27E-01
Q96K21	ZFYVE19	zinc finger FYVE-type containing 19	-1.000135151	0.6382731	0.281562868	9.44E-01	-0.380957325	0.862480694	-0.314588548	8.37E-01
P04196	HRG	histidine rich glycoprotein	-0.084028015	0.9860434	3.390073107	1.94E-02	-1.466699278	0.572520278	-0.286203849	8.97E-01
P02787	TF	transferrin	-1.768207228	0.606544	-0.962505645	8.17E-01	-1.784755861	0.49858242	-0.279340216	8.85E-01
P09327	VIL1	villin 1	-1.569225382	0.606544	-0.383499674	9.21E-01	-2.392069542	0.49858242	-0.267753086	8.97E-01
P02747	C1QC	complement C1q C chain	-0.898069028	0.7385922	1.353739687	5.97E-01	0.344761636	0.88674295	-0.267683697	8.78E-01
P25815	S100P	S100 calcium binding protein P	-1.226547877	0.7183056	-0.307650006	9.55E-01	-2.873228822	0.49858242	-0.263720497	9.05E-01
P06756	ITGAV	integrin subunit alpha V	0.052510139	0.9886331	-2.200512423	5.97E-01	0.16454292	0.9483529	-0.213042011	8.97E-01
P11678	EPX	eosinophil peroxidase	1.045768503	0.807657	0.073378424	9.87E-01	2.392598247	0.496346254	-0.191996764	9.25E-01
O14672	ADAM10	ADAM metallopeptidase domain 10	-1.780397623	0.606544	-0.765244324	6.47E-01	-1.001894926	0.621660822	-0.183389966	9.02E-01
P04439	HLA-A	major histocompatibility complex, class I, A	0.010623368	0.9953954	0.839911067	5.97E-01	-0.727648502	0.721528858	-0.15473857	9.01E-01
P06703	S100A6	S100 calcium binding protein A6	-1.727936381	0.606544	-2.054661971	3.55E-01	-0.549792853	0.807619793	-0.139016069	9.31E-01
P07602	PSAP	prosaposin	-1.701505493	0.606544	1.821514959	5.97E-01	-1.694023745	0.49858242	-0.129692824	9.36E-01
Q6IAA8	LAMTOR1	late endosomal/lysosomal adaptor, MAPK and MTOR activa	-1.933892339	0.606544	2.331160971	8.35E-02	-2.669177179	0.496346254	-0.125729875	9.36E-01
P05556	ITGB1	integrin subunit beta 1	-1.406864357	0.606544	-2.000703222	5.97E-01	-0.344573035	0.884208047	-0.105915172	9.48E-01
P11233	RALA	RAS like proto-oncogene A	-1.272471625	0.6253347	0.393582773	9.37E-01	-0.496745779	0.857313494	-0.099048956	9.57E-01
P01625	NA	NA	-1.117528993	0.6956246	1.456721633	5.97E-01	-1.068355463	0.655818184	-0.074894824	9.66E-01
P01891	HLA-A	major histocompatibility complex, class I, A	-1.237866665	0.606544	1.012799998	5.97E-01	-2.228159996	0.49858242	-0.069041458	9.59E-01
P17858	PFKL	phosphofructokinase, liver type	0.11304174	0.9614725	-0.551566921	8.39E-01	-0.604502544	0.761738131	-0.053180202	9.61E-01
P05026	ATP1B1	ATPase Na+/K+ transporting subunit beta 1	-0.034470825	0.9928211	-1.008465136	8.17E-01	0.770320373	0.734445265	-0.049518273	9.77E-01
P37802	TAGLN2	transgelin 2	-0.955963875	0.606544	0.782152262	5.97E-01	-0.52348472	0.775475352	-0.006485917	9.96E-01
P22392	NME2	NME/NM23 nucleoside diphosphate kinase 2	1.295070439	0.606544	-0.709315223	8.17E-01	0.777042264	0.49858242	-0.002288916	9.99E-01
Q14847	LASP1	LIM and SH3 protein 1	0.464329902	0.9106899	-1.383487393	6.01E-01	-0.93050893	0.655818184	-0.000268138	1.00E+00
P21730	C5AR1	complement C5a receptor 1	0.27294151	0.9189448	0.671746805	5.97E-01	-0.296360357	0.839453572	0.000706931	1.00E+00
Q13748	NA	NA	0.329865721	0.9189448	-0.824002544	8.17E-01	-0.381479542	0.838926923	0.011260966	9.93E-01
O75891	ALDH1L1	aldehyde dehydrogenase 1 family member L1	-0.912791449	0.606544	0.746829367	5.97E-01	-1.643024608	0.49858242	0.01463057	9.90E-01
P08670	VIM	vimentin	0.171882241	0.9371571	-0.748856676	8.17E-01	0.844626695	0.49858242	0.014917394	9.90E-01
P67936	TPM4	tropomyosin 4	-0.3180299	0.9225917	-0.50680031	8.92E-01	-2.18799468	0.49858242	0.01877633	9.90E-01
P60842	EIF4A1	eukaryotic translation initiation factor 4A1	0.309855479	0.9189448	-0.785251838	8.17E-01	0.890944251	0.49858242	0.019405164	9.89E-01
Q9NVJ2	ARL8B	ADP ribosylation factor like GTPase 8B	-0.966031141	0.606544	0.790389116	5.97E-01	0.869428027	0.49858242	0.032833837	9.77E-01
Q96BW5	PTER	phosphotriesterase related	1.334764794	0.606544	-2.002147191	5.97E-01	0.800858877	0.49858242	0.050302291	9.59E-01
O14828	SCAMP3	secretory carrier membrane protein 3	-0.779002374	0.606544	0.637365578	5.97E-01	0.701102136	0.49858242	0.050938094	9.54E-01
P17252	PRKCA	protein kinase C alpha	-0.79263986	0.606544	0.648523522	5.97E-01	-0.36884359	0.801293263	0.052841013	9.54E-01
P52943	CRIP2	cysteine rich protein 2	-1.958320973	0.606544	1.602262614	5.97E-01	-2.223829748	0.49858242	0.070766857	9.59E-01
Q96S37	SLC22A12	solute carrier family 22 member 12	0.211443989	0.9254988	-0.597978057	8.19E-01	-0.367362856	0.810864902	0.070918676	9.40E-01
P22061	PCMT1	protein-L-isoaspartate (D-aspartate) O-methyltransferase	1.196086861	0.606544	-0.558468621	8.24E-01	0.717652116	0.49858242	0.073811881	9.36E-01
P01600	NA	NA	1.763103779	0.606544	0.96169297	5.97E-01	1.057862267	0.49858242	0.076343487	9.54E-01
P12955	PEPD	peptidase D	1.585731044	0.606544	-2.378596565	5.97E-01	0.951438626	0.49858242	0.078738251	9.48E-01
P52758	RIDA	reactive intermediate imine deaminase A homolog	1.315391801	0.606544	-1.973087701	5.97E-01	0.789235081	0.49858242	0.091388209	9.29E-01
P02753	RBP4	retinol binding protein 4	0.244584705	0.9214958	-0.558199378	8.26E-01	0.755366933	0.49858242	0.100962584	9.21E-01
P02511	CRYAB	crystallin alpha B	0.234302053	0.9189448	-0.602002544	8.17E-01	0.691790054	0.49858242	0.102400184	9.13E-01
Q06495	SLC34A1	solute carrier family 34 member 1	-0.773111819	0.606544	0.632546034	5.97E-01	-1.391601275	0.49858242	0.10269265	9.13E-01
P00491	PNP	purine nucleoside phosphorylase	0.189138864	0.9341569	-0.599070549	8.26E-01	0.807280273	0.49858242	0.105930478	9.22E-01
O75015	FCGR3B	Fc fragment of IgG receptor IIIb	1.412994404	0.606544	0.77072422	5.97E-01	0.847796642	0.49858242	0.110098969	9.22E-01
P26641	EEF1G	eukaryotic translation elongation factor 1 gamma	0.259893892	0.9189448	0.710187499	5.97E-01	-0.344279596	0.835127935	0.111110852	9.16E-01
P08697	SERPINF2	serpin family F member 2	0.260988099	0.9189448	0.713254366	5.97E-01	0.784579803	0.49858242	0.111563559	9.16E-01
O43396	TXNL1	thioredoxin like 1	0.254191102	0.9189448	0.659487466	5.97E-01	-0.30582258	0.83794346	0.115570667	9.06E-01
P13760	HLA-DRB1	major histocompatibility complex, class II, DR beta 1	-1.170356073	0.606544	0.95756406	5.97E-01	-2.106640932	0.49858242	0.123069314	9.29E-01
P01598	NA	NA	1.502527632	0.606544	0.819560527	5.97E-01	0.901516579	0.49858242	0.123701386	9.19E-01
P02675	FGB	fibrinogen beta chain	-2.316919569	0.606544	2.15800307	5.97E-01	-1.233477108	0.502679643	0.140589982	9.44E-01
P28799	GRN	granulin precursor	-1.929973674	0.606544	1.579069369	5.97E-01	-2.038355795	0.49858242	0.141191491	9.24E-01
P35613	BSG	basigin (Ok blood group)	0.293218678	0.9189448	-0.719779413	8.17E-01	-0.316783249	0.839453572	0.14134939	8.98E-01
P63218	GNG5	G protein subunit gamma 5	-0.850296983	0.606544	0.695697531	5.97E-01	-0.29771536	0.843844089	0.144147331	8.94E-01
P07358	C8B	complement C8 beta chain	0.714975822	0.8434492	0.262940633	9.44E-01	0.540786131	0.800413265	0.151134768	9.25E-01
Q1EHB4	SLC5A12	solute carrier family 5 member 12	-0.966323401	0.606544	0.790628237	5.97E-01	-0.351273266	0.838926923	0.152269081	8.99E-01
Q9BV40	VAMP8	vesicle associated membrane protein 8	0.314220035	0.9189448	0.734755702	5.97E-01	0.808231272	0.49858242	0.158670878	8.89E-01
P19827	ITIH1	inter-alpha-trypsin inhibitor heavy chain 1	1.059167555	0.7795884	1.693817379	6.69E-01	-0.932010892	0.768785785	0.159200605	9.39E-01
O75594	PGLYRP1	peptidoglycan recognition protein 1	1.036247994	0.7649939	-1.155234018	7.60E-01	0.85009905	0.682969445	0.160221794	9.29E-01
P62330	ARF6	ADP ribosylation factor 6	0.48636926	0.8556187	-1.242767924	5.97E-01	1.420892429	0.496346254	0.164962805	8.97E-01
P61088	UBE2N	ubiquitin conjugating enzyme E2 N	1.315890917	0.606544	-1.973836375	5.97E-01	0.78953455	0.49858242	0.16729234	8.81E-01
P01903	HLA-DRA	major histocompatibility complex, class II, DR alpha	-0.947352308	0.606544	0.775106434	5.97E-01	-0.322784446	0.845401319	0.16855875	8.88E-01
Q08257	CRYZ	crystallin zeta	1.430144577	0.606544	-2.145216866	5.97E-01	0.858086746	0.49858242	0.169745483	8.88E-01
P27169	PON1	paraoxonase 1	0.86519669	0.8271667	1.961045802	5.97E-01	0.795505377	0.717089821	0.185583275	9.22E-01
P59666	DEFA3	defensin alpha 3	2.5636441	0.606544	1.398351327	5.97E-01	1.53818646	0.49858242	0.192329258	9.24E-01
Q9GIY3	HLA-DRB1	major histocompatibility complex, class II, DR beta 1	-1.088772048	0.606544	0.890813494	5.97E-01	0.979894843	0.49858242	0.194216433	8.88E-01
P13640	MT1G	metallothionein 1G	1.757741288	0.606544	-1.546255621	5.97E-01	0.497875316	0.800413265	0.19690526	8.99E-01
P82980	RBP5	retinol binding protein 5	1.600928577	0.606544	-2.401392866	5.97E-01	0.960557146	0.49858242	0.206246651	8.79E-01
Q9Y6W5	WASF2	WASP family member 2	-0.837842116	0.606544	0.685507186	5.97E-01	-0.217545631	0.881117832	0.209722421	8.39E-01
Q10589	BST2	bone marrow stromal cell antigen 2	0.362012357	0.8968647	-0.797618728	7.94E-01	-0.230888926	0.878195	0.210274801	8.44E-01
P21333	FLNA	filamin A	1.526138358	0.606544	-0.561708456	8.38E-01	0.915683015	0.49858242	0.212716938	8.69E-01
Q99832	CCT7	chaperonin containing TCP1 subunit 7	0.362397025	0.8923854	0.671289734	5.97E-01	-0.19934618	0.888817484	0.21759379	8.29E-01
P30508	HLA-C	major histocompatibility complex, class I, C	0.432757099	0.9189448	-1.032449502	8.13E-01	-0.414978123	0.846141883	0.220347071	8.88E-01
Q96NY7	CLIC6	chloride intracellular channel 6	-2.117240409	0.606544	1.732287608	5.97E-01	-2.337920875	0.49858242	0.225908768	8.97E-01
P01617	NA	NA	0.750182184	0.8539819	0.329984366	9.37E-01	0.519810516	0.83794346	0.230909219	9.02E-01
P09871	C1S	complement C1s	1.365451395	0.606544	0.74479167	5.97E-01	0.819270837	0.49858242	0.234798186	8.34E-01
P02656	APOC3	apolipoprotein C3	-1.155711747	0.606544	0.945582338	5.97E-01	-2.080281144	0.49858242	0.235921842	8.73E-01
O43653	PSCA	prostate stem cell antigen	-0.954119616	0.7898154	-2.131526517	5.97E-01	-2.811678694	0.181135969	0.239264913	9.05E-01
P29373	CRABP2	cellular retinoic acid binding protein 2	-1.98933376	0.606544	-0.07134344	9.82E-01	-2.526086017	0.49858242	0.257036068	8.76E-01
P62993	GRB2	growth factor receptor bound protein 2	-0.75796538	0.6738958	0.237898766	9.37E-01	-0.831920949	0.636970154	0.257259673	8.46E-01
P00739	HPR	haptoglobin-related protein	0.749598447	0.9041836	0.013707553	9.97E-01	0.797503676	0.796476479	0.265747348	9.13E-01
P02768	ALB	albumin	-0.119630708	0.9371571	0.158521634	9.17E-01	-0.094365338	0.941443756	0.280803451	6.56E-01
P36955	SERPINF1	serpin family F member 1	0.338108037	0.9203876	1.494918712	5.97E-01	-0.950371787	0.617067843	0.283295926	8.44E-01
P00751	CFB	complement factor B	0.204134106	0.9679774	0.972119128	8.25E-01	-0.421290303	0.901781911	0.293974135	9.01E-01
P26022	PTX3	pentraxin 3	1.571003786	0.606544	0.856911156	5.97E-01	0.942602272	0.49858242	0.314715834	8.08E-01
P02533	KRT14	keratin 14	-1.340771387	0.606544	1.096994771	5.97E-01	-0.877810766	0.738257663	0.352797381	8.30E-01
P01024	C3	complement C3	-1.997601923	0.606544	1.077835312	8.26E-01	-1.925180641	0.49858242	0.352945288	8.66E-01
P02792	FTL	ferritin light chain	-1.481496514	0.606544	4.578704819	0.00E+00	-0.730962278	0.767371774	0.367070973	8.45E-01
P04275	VWF	von Willebrand factor	-2.171315519	0.606544	3.353714944	5.97E-01	-0.385672946	0.917193947	0.393975139	8.73E-01
Q16270	IGFBP7	insulin like growth factor binding protein 7	-1.171344023	0.606544	0.958372382	5.97E-01	-2.108419241	0.49858242	0.423759399	7.62E-01
P08519	LPA	lipoprotein(a)	1.852751008	0.606544	1.010591459	5.97E-01	1.111650605	0.49858242	0.429928929	7.71E-01
P02746	C1QB	complement C1q B chain	-0.351019623	0.9311092	2.617404175	8.35E-02	-1.615555416	0.49858242	0.43862661	8.12E-01
O43866	CD5L	CD5 molecule like	-0.037141333	0.9928211	1.108577728	8.17E-01	0.629079822	0.837774529	0.44162404	8.27E-01
Q9NRD8	DUOX2	dual oxidase 2	-0.371394304	0.9311092	-0.923932098	8.31E-01	-1.195586989	0.600120045	0.443044071	8.22E-01
P46940	IQGAP1	IQ motif containing GTPase activating protein 1	-0.150976642	0.9687687	0.227535714	9.55E-01	-0.498931303	0.846688909	0.45002152	8.05E-01

P19823	ITIH2	inter-alpha-trypsin inhibitor heavy chain 2	0.867116353	0.8524024	1.633325665	6.17E-01	0.495891547	0.867184582	0.450802228	8.28E-01
P35908	KRT2	keratin 2	-2.119561175	0.606544	2.656425837	9.04E-02	-1.912215999	0.49858242	0.450825647	8.14E-01
Q15113	PCOLCE	procollagen C-endopeptidase enhancer	0.529606179	0.606544	0.288876098	5.97E-01	0.317763708	0.49858242	0.453948154	4.05E-01
Q8N357	SLC35F6	solute carrier family 35 member F6	-0.742579916	0.8271667	1.177237914	6.33E-01	-0.023427911	0.993572665	0.456081407	7.94E-01
O43776	NARS	asparaginyl-tRNA synthetase	0.536145637	0.606544	0.292443075	5.97E-01	0.321687382	0.49858242	0.459553403	4.05E-01
P01892	HLA-A	major histocompatibility complex, class I, A	0.749634111	0.8539819	-1.86415766	5.97E-01	-0.852102961	0.734445265	0.460715828	7.99E-01
P46059	SLC15A1	solute carrier family 15 member 1	-0.362607412	0.606544	0.296678792	5.97E-01	0.326346671	0.49858242	0.46620953	4.05E-01
P04220	NA	NA	2.00111844	0.606544	1.091519149	5.97E-01	1.200671064	0.49858242	0.468903078	7.69E-01
Q9HAC8	UBTD1	ubiquitin domain containing 1	-0.366121761	0.606544	0.299554168	5.97E-01	0.329509585	0.49858242	0.470727979	4.05E-01
P02538	KRT6A	keratin 6A	-1.351306464	0.606544	1.105614379	5.97E-01	-1.015305711	0.706275784	0.47217445	7.70E-01
P13647	KRT5	keratin 5	-1.184250078	0.606544	0.968931882	5.97E-01	-0.957024969	0.68972714	0.473834769	7.36E-01
Q15435	PPP1R7	protein phosphatase 1 regulatory subunit 7	0.556257892	0.606544	0.303413396	5.97E-01	0.333754736	0.49858242	0.476792479	4.05E-01
P62491	RAB11A	RAB11A, member RAS oncogene family	-1.137395836	0.6696571	-1.779688916	5.97E-01	-0.262273455	0.909610229	0.477785939	7.58E-01
Q9NRM0	SLC2A9	solute carrier family 2 member 9	0.559946784	0.606544	-0.839920175	5.97E-01	0.33596807	0.49858242	0.479954386	4.05E-01
Q00325	SLC25A3	solute carrier family 25 member 3	-0.374335387	0.606544	0.306274408	5.97E-01	0.336901848	0.49858242	0.481288355	4.05E-01
P60709	ACTB	actin beta	-0.182949419	0.9042183	0.128512979	9.21E-01	-0.381902812	0.586423149	0.485449881	4.05E-01
P45974	USP5	ubiquitin specific peptidase 5	0.568304451	0.606544	-0.852456677	5.97E-01	0.340982671	0.49858242	0.487118101	4.05E-01
Q9NQT8	KIF13B	kinesin family member 13B	0.570710442	0.606544	0.311296605	5.97E-01	0.342426266	0.49858242	0.489180379	4.05E-01
P12111	COL6A3	collagen type VI alpha 3 chain	-0.384455003	0.606544	0.314554093	5.97E-01	-0.692019005	0.49858242	0.494299289	4.05E-01
Q16851	UGP2	UDP-glucose pyrophosphorylase 2	0.579590158	0.606544	0.316140086	5.97E-01	0.347754095	0.49858242	0.496791564	4.05E-01
Q15797	SMAD1	SMAD family member 1	-0.386633383	0.606544	0.316336404	5.97E-01	0.347970044	0.49858242	0.497100063	4.05E-01
Q9H0R4	HDHD2	haloacid dehalogenase like hydrolase domain containing 2	0.581941214	0.606544	0.31742248	5.97E-01	0.349164729	0.49858242	0.498806755	4.05E-01
Q96HP8	TMEM176A	transmembrane protein 176A	-0.388028457	0.606544	0.317477828	5.97E-01	0.349225611	0.49858242	0.49889373	4.05E-01
P21980	TGM2	transglutaminase 2	0.582093398	0.606544	0.31750549	5.97E-01	0.349256039	0.49858242	0.498937198	4.05E-01
P61457	PCBD1	pterin-4 alpha-carbinolamine dehydratase 1	0.583255547	0.606544	-0.874883321	5.97E-01	0.349953328	0.49858242	0.499933326	4.05E-01
Q5VWZ2	LYPLAL1	lysophospholipase like 1	0.584359636	0.606544	0.31874162	5.97E-01	0.350615782	0.49858242	0.500879688	4.05E-01
Q9P265	DIP2B	disco interacting protein 2 homolog B	-0.391883176	0.606544	0.32063169	5.97E-01	0.352694859	0.49858242	0.503849798	4.05E-01
Q92630	DYRK2	dual specificity tyrosine phosphorylation regulated kinase 2	0.587824764	0.606544	-0.881737147	5.97E-01	0.352694859	0.49858242	0.503849798	4.05E-01
O95721	SNAP29	synaptosome associated protein 29	-0.393278777	0.606544	0.321773545	5.97E-01	0.3539509	0.49858242	0.505644143	4.05E-01
Q05513	PRKCZ	protein kinase C zeta	-0.393729403	0.606544	0.322142238	5.97E-01	0.354356462	0.49858242	0.506223518	4.05E-01
Q6PHR2	ULK3	unc-51 like kinase 3	-0.394018128	0.606544	0.322378468	5.97E-01	0.354616315	0.49858242	0.506594736	4.05E-01
Q16864	ATP6V1F	ATPase H+ transporting V1 subunit F	-0.394943395	0.606544	0.323135505	5.97E-01	0.355449055	0.49858242	0.507784364	4.05E-01
P30711	GSTT1	glutathione S-transferase theta 1	0.596789549	0.606544	0.325521572	5.97E-01	0.35807373	0.49858242	0.511533899	4.05E-01
Q99653	CHP1	calcineurin like EF-hand protein 1	0.597761639	0.606544	-0.896642459	5.97E-01	0.358656984	0.49858242	0.512367119	4.05E-01
P19801	AOC1	amine oxidase copper containing 1	-0.399396531	0.606544	0.32677898	5.97E-01	0.359456878	0.49858242	0.513509825	4.05E-01
P21283	ATP6V1C1	ATPase H+ transporting V1 subunit C1	0.601639374	0.606544	-0.902459062	5.97E-01	0.360983625	0.49858242	0.515690892	4.05E-01
P13498	CYBA	cytochrome b-245 alpha chain	0.603567215	0.606544	0.329218481	5.97E-01	0.362140329	0.49858242	0.517343327	4.05E-01
P55083	MFAP4	microfibril associated protein 4	0.605031743	0.606544	0.330017314	5.97E-01	0.363019046	0.49858242	0.518598637	4.05E-01
P52790	HK3	hexokinase 3	0.609263528	0.606544	0.332325561	5.97E-01	0.365558117	0.49858242	0.522225881	4.05E-01
P21291	CSRP1	cysteine and glycine rich protein 1	0.610251376	0.606544	0.332864387	5.97E-01	0.366150826	0.49858242	0.523072608	4.05E-01
P15428	HPGD	15-hydroxyprostaglandin dehydrogenase	-0.612680257	0.8857221	-2.825597285	8.35E-02	-2.469124111	0.49858242	0.524605325	7.66E-01
Q9H201	EPN3	epsin 3	-0.408505978	0.606544	0.334232164	5.97E-01	0.36765538	0.49858242	0.525221972	4.05E-01
P08637	FCGR3A	Fc fragment of IgG receptor IIIa	-0.347869816	0.9189448	-0.508470439	9.00E-01	0.071754585	0.98183702	0.525317136	6.65E-01
O43278	SPINT1	serine peptidase inhibitor, Kunitz type 1	0.61305399	0.606544	0.334393085	5.97E-01	0.367832394	0.49858242	0.525474849	4.05E-01
O75223	GGCT	gamma-glutamylcyclotransferase	0.614979826	0.606544	0.335443541	5.97E-01	0.368987895	0.49858242	0.527125565	4.05E-01
P35321	SPRR1A	small proline rich protein 1A	-0.410650563	0.606544	-0.923963767	5.97E-01	-0.739171014	0.49858242	0.527979295	4.05E-01
P25685	DNAJB1	DnaJ heat shock protein family (Hsp40) member B1	-0.410678144	0.606544	0.336009391	5.97E-01	0.36961033	0.49858242	0.528014757	4.05E-01
Q7Z7D3	VTCN1	V-set domain containing T cell activation inhibitor 1	-0.412021307	0.606544	0.337108342	5.97E-01	-0.741638353	0.49858242	0.529741681	4.05E-01
P09619	PDGFRB	platelet derived growth factor receptor beta	0.619780158	0.606544	-0.929670236	5.97E-01	0.371868095	0.49858242	0.531240135	4.05E-01
O95154	AKR7A3	aldo-keto reductase family 7 member A3	0.620022641	0.606544	-0.930033961	5.97E-01	0.372013584	0.49858242	0.531447978	4.05E-01
O76054	SEC14L2	SEC14 like lipid binding 2	0.620305092	0.606544	0.338348232	5.97E-01	0.372183055	0.49858242	0.531690079	4.05E-01
P24666	ACP1	acid phosphatase 1	0.620627309	0.606544	-0.930940964	5.97E-01	0.372376386	0.49858242	0.531966265	4.05E-01
Q9HDC9	APMAP	adipocyte plasma membrane associated protein	0.621149587	0.606544	0.338808866	5.97E-01	0.372689752	0.49858242	0.532413932	4.05E-01
P14136	GFAP	glial fibrillary acidic protein	-0.414686487	0.606544	0.339288944	5.97E-01	0.373217838	0.49858242	0.53316834	4.05E-01
P09382	LGALS1	galectin 1	0.623578651	0.606544	0.340133809	5.97E-01	0.37414719	0.49858242	0.534495986	4.05E-01
P21399	ACO1	aconitase 1	-0.416271174	0.606544	0.340585506	5.97E-01	0.374644057	0.49858242	0.535205795	4.05E-01
O95967	EFEMP2	EGF containing fibulin extracellular matrix protein 2	-0.416689968	0.606544	-0.937552427	5.97E-01	-0.750041942	0.49858242	0.535744244	4.05E-01
P46108	CRK	CRK proto-oncogene, adaptor protein	0.625465463	0.606544	-0.938198195	5.97E-01	0.375279278	0.49858242	0.536113254	4.05E-01
O75970	MPDZ	multiple PDZ domain crumbs cell polarity complex compone	-0.418426722	0.606544	0.342349136	5.97E-01	0.37658405	0.49858242	0.537977214	4.05E-01
P49588	AARS	alanyl-tRNA synthetase	0.629137961	0.606544	0.343166161	5.97E-01	0.377482777	0.49858242	0.53926111	4.05E-01
Q5T0N5	FNBP1L	formin binding protein 1 like	-0.419832401	0.606544	0.343499238	5.97E-01	0.377849161	0.49858242	0.539784516	4.05E-01
Q15274	QPRT	quinolinate phosphoribosyltransferase	0.629786693	0.606544	-0.94468004	5.97E-01	0.377872016	0.49858242	0.539817165	4.05E-01
O60488	ACSL4	acyl-CoA synthetase long chain family member 4	-0.419984678	0.606544	0.343623827	5.97E-01	0.37798621	0.49858242	0.5399803	4.05E-01
Q9Y485	DMXL1	Dmx like 1	-0.420187389	0.606544	0.343789682	5.97E-01	0.37816865	0.49858242	0.540240929	4.05E-01
Q9Y226	SLC22A13	solute carrier family 22 member 13	-0.420692555	0.606544	0.344203	5.97E-01	0.3786233	0.49858242	0.540890429	4.05E-01
P05787	KRT8	keratin 8	-0.420843659	0.606544	-0.946898232	5.97E-01	-0.757518585	0.49858242	0.541084704	4.05E-01
Q06124	PTPN11	protein tyrosine phosphatase non-receptor type 11	-0.421295739	0.606544	0.344696514	5.97E-01	0.379166165	0.49858242	0.54166595	4.05E-01
O60610	DIAPH1	diaphanous related formin 1	-0.422764792	0.606544	0.345898466	5.97E-01	0.380488313	0.49858242	0.543554733	4.05E-01
O60268	KIAA0513	KIAA0513	0.641016143	0.606544	0.349645169	5.97E-01	0.384609686	0.49858242	0.549442408	4.05E-01
Q15181	PPA1	pyrophosphatase (inorganic) 1	0.641797616	0.606544	-0.962696424	5.97E-01	0.38507857	0.49858242	0.550112242	4.05E-01
O75674	TOM1L1	target of myb1 like 1 membrane trafficking protein	-0.431947264	0.606544	0.353411398	5.97E-01	0.388752537	0.49858242	0.555360768	4.05E-01
Q9NV96	TMEM30A	transmembrane protein 30A	-0.145601185	0.9487033	-0.339489064	9.20E-01	-0.059681174	0.983062223	0.557427062	5.92E-01
Q9UJU6	DBNL	drebrin like	-0.434156933	0.606544	0.355219308	5.97E-01	-0.781482479	0.49858242	0.55820177	4.05E-01
P00740	F9	coagulation factor IX	0.651235399	0.606544	0.355219308	5.97E-01	0.390741239	0.49858242	0.55820177	4.05E-01
Q9BUF7	CRB3	crumbs cell polarity complex component 3	-0.434669223	0.606544	0.355638455	5.97E-01	0.391202301	0.49858242	0.558860429	4.05E-01
Q96FW1	OTUB1	OTU deubiquitinase, ubiquitin aldehyde binding 1	0.65280192	0.606544	0.356073775	5.97E-01	0.391681152	0.49858242	0.559544503	4.05E-01
Q13131	PRKAA1	protein kinase AMP-activated catalytic subunit alpha 1	0.654255226	0.606544	-0.981382839	5.97E-01	0.392553136	0.49858242	0.560790194	4.05E-01
O94804	STK10	serine/threonine kinase 10	-0.436782335	0.606544	0.357367365	5.97E-01	-0.786208202	0.49858242	0.561577287	4.05E-01
Q8IV45	UNC5CL	unc-5 family C-terminal like	-0.43706542	0.606544	0.35759898	5.97E-01	-0.786717757	0.49858242	0.561941255	4.05E-01
Q96TA1	NIBAN2	niban apoptosis regulator 2	-1.108476255	0.606544	0.530937399	8.38E-01	-1.55103919	0.49858242	0.563297517	6.52E-01
P31948	STIP1	stress induced phosphoprotein 1	0.657382761	0.606544	0.358572415	5.97E-01	0.394429657	0.49858242	0.563470938	4.05E-01
Q8NFT2	STEAP2	STEAP2 metalloreductase	0.657608531	0.606544	0.358695562	5.97E-01	0.394565119	0.49858242	0.563664455	4.05E-01
P37837	TALDO1	transaldolase 1	0.657640759	0.606544	0.358713141	5.97E-01	0.394584455	0.49858242	0.563692079	4.05E-01
Q9P0T7	TMEM9	transmembrane protein 9	-1.252360242	0.606544	1.02465838	5.97E-01	-1.065604751	0.49858242	0.566525656	5.81E-01
P35240	NF2	neurofibromin 2	-0.440828635	0.606544	0.360677974	5.97E-01	0.396745771	0.49858242	0.566779673	4.05E-01
P50591	TNFSF10	TNF superfamily member 10	0.665754038	0.606544	0.363138566	5.97E-01	0.399452423	0.49858242	0.570646318	4.05E-01
P61970	NUTF2	nuclear transport factor 2	0.665815414	0.606544	0.363172044	5.97E-01	0.399489248	0.49858242	0.570698926	4.05E-01
Q9UL46	PSME2	proteasome activator subunit 2	0.667068668	0.606544	0.363855637	5.97E-01	0.400241201	0.49858242	0.571773144	4.05E-01
P13693	TPT1	tumor protein, translationally-controlled 1	0.667068668	0.606544	0.363855637	5.97E-01	0.400241201	0.49858242	0.571773144	4.05E-01
Q9UBC9	SPRR3	small proline rich protein 3	-0.445016554	0.606544	-1.001287246	5.97E-01	-0.801029797	0.49858242	0.572164141	4.05E-01
Q9UL54	TAOK2	TAO kinase 2	-0.445440912	0.606544	0.364451656	5.97E-01	0.400896821	0.49858242	0.572709745	4.05E-01
P14543	NID1	nidogen 1	0.671278405	0.606544	0.366151857	5.97E-01	0.402767043	0.49858242	0.57538149	4.05E-01
O15244	SLC22A2	solute carrier family 22 member 2	0.673021147	0.606544	-1.009531721	5.97E-01	0.403812688	0.49858242	0.576875269	4.05E-01
P78371	CCT2	chaperonin containing TCP1 subunit 2	0.673929574	0.606544	0.36759795	5.97E-01	0.404357745	0.49858242	0.577653921	4.05E-01
P53634	CTSC	cathepsin C	-0.450121006	0.606544	0.368280823	5.97E-01	0.405108905	0.49858242	0.578727008	4.05E-01
P01011	SERPINA3	serpin family A member 3	0.877649492	0.6585388	-0.303211062	9.25E-01	-0.023888298	0.993572665	0.581237026	6.00E-01
P35268	RPL22	ribosomal protein L22	0.67839889	0.606544	0.370035758	5.97E-01	0.407039334	0.49858242	0.581484763	4.05E-01
Q14118	DAG1	dystroglycan 1	0.678683159	0.606544	-1.018024738	5.97E-01	0.407209895	0.49858242	0.581728422	4.05E-01
P98161	PKD1	polycystin 1, transient receptor potential channel interacting	0.681136049	0.606544	-1.021704074	5.97E-01	0.40868163	0.49858242	0.583830899	4.05E-01
Q92542	NCSTN	nicastrin	-0.455591848	0.606544	0.372756967	5.97E-01	0.410032663	0.49858242	0.585760948	4.05E-01
Q8N5J2	MINDY1	MINDY lysine 48 deubiquitinase 1	-0.455775777	0.606544	0.372907454	5.97E-01	0.410198199	0.49858242	0.585997427	4.05E-01
Q5T2W1	PDZK1	PDZ domain containing 1	-0.664003188	0.8757534	0.779589284	8.77E-01	-0.229834069	0.948759698	0.586149525	7.33E-01
Q9BRA2	TXNDC17	thioredoxin domain containing 17	0.684570884	0.606544	-1.026856325	5.97E-01	0.41074253	0.49858242	0.586775043	4.05E-01
Q13057	COASY	Coenzyme A synthase	0.68536408	0.606544	-1.02804612	5.97E-01	0.411218448	0.49858242	0.587454926	4.05E-01
P11597	CETP	cholesteryl ester transfer protein	0.687990085	0.606544	-1.031985128	5.97E-01	0.412794051	0.49858242	0.589705787	4.05E-01
Q6EMK4	VASN	vasorin	0.688178	0.606544	-1.032267	5.97E-01	0.4129068	0.49858242	0.589866857	4.05E-01
O95867	LY6G6C	lymphocyte antigen 6 family member G6C	-0.459017615	0.606544	-1.032789634	5.97E-01	-0.826231707	0.49858242	0.590165505	4.05E-01
O14791	APOL1	apolipoprotein L1	-0.4604012	0.606544	0.376691891	5.97E-01	-0.828722161	0.49858242	0.591944401	4.05E-01
Q9HBA9	NA	NA	-0.460841036	0.606544	0.377051757	5.97E-01	0.414756932	0.49858242	0.592509903	4.05E-01
P05156	CFI	complement factor I	-0.461802589	0.606544	-1.039055825	5.97E-01	-0.83124466	0.49858242	0.593746186	4.05E-01
P34096	RNASE4	ribonuclease A family member 4	0.693095089	0.606544	0.378051867	5.97E-01	0.415857054	0.49858242	0.594081505	4.05E-01
Q4KMQ2	ANO6	anoctamin 6	-1.263880367	0.606544	-1.530583209	5.97E-01	-1.617179993	0.49858242	0.594198395	7.08E-01
O00401	WASL	WASP like actin nucleation promoting factor	-0.289974953	0.9189448	-0.148449217	9.55E-01	0.00031759	0.999924593	0.594669624	5.56E-01
P51798	CLCN7	chloride voltage-gated channel 7	-0.462600455	0.606544	0.378491282	5.97E-01	0.41634041	0.49858242	0.594772014	4.05E-01
Q9Y2A7	NCKAP1	NCK associated protein 1	-0.46428139	0.606544	0.379866592	5.97E-01	0.417853251	0.49858242	0.596933216	4.05E-01
Q03167	TGFBR3	transforming growth factor beta receptor 3	0.69662624	0.606544	-1.04493936	5.97E-01	0.417975744	0.49858242	0.597108206	4.05E-01
Q86VE9	SERINC5	serine incorporator 5	0.6970338	0.606544	0.380200255	5.97E-01	0.41822028	0.49858242	0.597457543	4.05E-01
Q9NRR3	CDC42SE2	CDC42 small effector 2	-0.465803021	0.606544	0.381111563	5.97E-01	0.419222719	0.49858242	0.598889598	4.05E-01
O00764	PDXK	pyridoxal kinase	0.698855589	0.606544	0.381193957	5.97E-01	0.419313353	0.49858242	0.599019076	4.05E-01
Q7KZF4	SND1	staphylococcal nuclease and tudor domain containing 1	0.698931066	0.606544	0.381235127	5.97E-01	0.41935864	0.49858242	0.599083771	4.05E-01
Q9Y365	STARD10	StAR related lipid transfer domain containing 10	0.700333739	0.606544	0.382000222	5.97E-01	0.420200244	0.49858242	0.600286062	4.05E-01
Q9NP72	RAB18	RAB18, member RAS oncogene family	0.70035868	0.606544	-1.05053802	5.97E-01	0.420215208	0.49858242	0.60030744	4.05E-01
Q9Y3L5	RAP2C	RAP2C, member of RAS oncogene family	-0.46705532	0.606544	0.382136171	5.97E-01	0.420349788	0.49858242	0.600499697	4.05E-01
Q9Y512	SAMM50	SAMM50 sorting and assembly machinery component	-0.467237809	0.606544	0.38228548	5.97E-01	0.420514028	0.49858242	0.600734326	4.05E-01
P17987	TCP1	t-complex 1	0.701452395	0.606544	0.382610397	5.97E-01	0.420871437	0.49858242	0.60124491	4.05E-01
Q86VN1	VPS36	vacuolar protein sorting 36 homolog	-0.467997715	0.606544	0.382907222	5.97E-01	0.421197944	0.49858242	0.601711348	4.05E-01
P11226	MBL2	mannose binding lectin 2	-0.46909532	0.606544	0.383805262	5.97E-01	-0.844371577	0.49858242	0.603122555	4.05E-01
P06310	IGKV2-30	NA	-0.469777883	0.606544	0.384363722	5.97E-01	-0.845600189	0.49858242	0.604000135	4.05E-01
P61421	ATP6V0D1	ATPase H+ transporting V0 subunit d1	-1.262745734	0.606544	1.0331556	5.97E-01	-1.032872738	0.49858242	0.606520427	5.58E-01

Q99985	SEMA3C	semaphorin 3C	0.70915054	0.606544	0.386809385	5.97E-01	0.425490324	0.49858242	0.60784332	4.05E-01
P06727	APOA4	apolipoprotein A4	0.324589419	0.9341569	1.754622217	5.97E-01	0.39967389	0.884208047	0.608574009	7.16E-01
P13797	PLS3	plastin 3	-0.47345882	0.606544	0.387375398	5.97E-01	0.426112938	0.49858242	0.608732769	4.05E-01
Q9NQX4	MYO5C	myosin VC	0.71084523	0.606544	0.387733762	5.97E-01	0.426507138	0.49858242	0.609295912	4.05E-01
Q6PIF6	MYO7B	myosin VIIB	-0.474161909	0.606544	0.387950653	5.97E-01	-0.853491436	0.49858242	0.60963674	4.05E-01
P12259	F5	coagulation factor V	0.712499677	0.606544	0.388636187	5.97E-01	0.427499806	0.49858242	0.610714009	4.05E-01
Q6ZWJ1	STXBP4	syntaxin binding protein 4	-0.475477948	0.606544	0.389027412	5.97E-01	0.427930153	0.49858242	0.61132879	4.05E-01
Q9BXJ7	AMN	amnion associated transmembrane protein	-0.476641586	0.606544	0.38997948	5.97E-01	0.428977428	0.49858242	0.612824896	4.05E-01
Q9BUD6	SPON2	spondin 2	0.715145053	0.606544	0.39007912	5.97E-01	0.429087032	0.49858242	0.612981474	4.05E-01
O75264	SMIM24	small integral membrane protein 24	-0.180900284	0.9614725	-1.077877625	8.17E-01	-0.910292579	0.684236411	0.613410843	7.19E-01
Q14117	DPYS	dihydropyrimidinase	0.719024869	0.606544	-1.078537303	5.97E-01	0.431414921	0.49858242	0.61630703	4.05E-01
Q8WW52	FAM151A	family with sequence similarity 151 member A	-0.403201081	0.9189448	-0.697438939	8.46E-01	0.25852943	0.917193947	0.618624561	6.89E-01
Q9BW30	TPPP3	tubulin polymerization promoting protein family member 3	0.723707627	0.606544	-1.085561441	5.97E-01	0.434224577	0.49858242	0.620320824	4.05E-01
O00214	LGALS8	galectin 8	-0.483550439	0.606544	0.395632177	5.97E-01	0.435195395	0.49858242	0.621707707	4.05E-01
P78369	CLDN10	claudin 10	0.725411514	0.606544	-1.088117271	5.97E-01	0.435246908	0.49858242	0.621781298	4.05E-01
P49368	CCT3	chaperonin containing TCP1 subunit 3	0.725583092	0.606544	0.395772596	5.97E-01	0.435349855	0.49858242	0.621928364	4.05E-01
P41743	PRKCI	protein kinase C iota	0.727374077	0.606544	-1.091061116	5.97E-01	0.436424447	0.49858242	0.623463495	4.05E-01
Q7Z7M0	MEGF8	multiple EGF like domains 8	-0.485184351	0.606544	-1.091664789	5.97E-01	-0.873331832	0.49858242	0.623808451	4.05E-01
Q9BT76	UPK3B	uroplakin 3B	-0.485704976	0.606544	-1.092836197	5.97E-01	-0.874268957	0.49858242	0.624477827	4.05E-01
P26012	ITGB8	integrin subunit beta 8	0.728830982	0.606544	-1.093246473	5.97E-01	0.437298589	0.49858242	0.62471227	4.05E-01
O60749	SNX2	sorting nexin 2	0.728873021	0.606544	-1.093309532	5.97E-01	0.437323813	0.49858242	0.624748304	4.05E-01
Q7Z3F1	GPR155	G protein-coupled receptor 155	-0.486055374	0.606544	0.39768167	5.97E-01	0.437449837	0.49858242	0.624928338	4.05E-01
O94856	NFASC	neurofascin	0.729753416	0.606544	-1.094630124	5.97E-01	0.43785205	0.49858242	0.625502928	4.05E-01
Q86UX7	FERMT3	fermitin family member 3	0.731810414	0.606544	0.399169317	5.97E-01	0.439086248	0.49858242	0.627266069	4.05E-01
P40227	CCT6A	chaperonin containing TCP1 subunit 6A	-0.487997441	0.606544	0.399270633	5.97E-01	0.439197697	0.49858242	0.627425281	4.05E-01
Q8NEU8	APPL2	adaptor protein, phosphotyrosine interacting with PH domair	0.732367035	0.606544	0.399472928	5.97E-01	0.439420221	0.49858242	0.627743173	4.05E-01
Q13510	ASAH1	N-acylsphingosine amidohydrolase 1	-0.074074729	0.9860434	0.470606821	9.17E-01	-0.574306372	0.835127935	0.629535155	6.93E-01
Q6UWE0	LRSAM1	leucine rich repeat and sterile alpha motif containing 1	-0.489987218	0.606544	0.400898633	5.97E-01	0.440988496	0.49858242	0.629983566	4.05E-01
P27105	STOM	stomatin	-0.20771867	0.9124383	-0.439794613	5.97E-01	-0.066385812	0.938574723	0.630624737	3.11E-01
O75882	ATRN	attractin	-0.494539203	0.606544	0.404622984	5.97E-01	0.445085283	0.49858242	0.635836118	4.05E-01
Q9BXI6	TBC1D10A	TBC1 domain family member 10A	0.742022592	0.606544	0.404739595	5.97E-01	0.445213555	0.49858242	0.636019364	4.05E-01
P02679	FGG	fibrinogen gamma chain	-2.948490165	0.606544	2.70976406	5.97E-01	-1.664353142	0.49858242	0.636231193	7.61E-01
P12273	PIP	prolactin induced protein	-0.495917882	0.606544	0.405750995	5.97E-01	-0.892652188	0.49858242	0.637608706	4.05E-01
Q9UKU9	ANGPTL2	angiopoietin like 2	-0.496594496	0.606544	-1.117337616	5.97E-01	-0.893870093	0.49858242	0.638478638	4.05E-01
P33908	MAN1A1	mannosidase alpha class 1A member 1	-0.496861453	0.606544	0.406523007	5.97E-01	-0.894350615	0.49858242	0.638821868	4.05E-01
Q14CN2	CLCA4	chloride channel accessory 4	-0.498299559	0.606544	0.407699639	5.97E-01	-0.896939207	0.49858242	0.640670862	4.05E-01
Q9P2M7	CGN	cingulin	-0.500311687	0.606544	-1.125701295	5.97E-01	-0.900561036	0.49858242	0.643257883	4.05E-01
Q9Y376	CAB39	calcium binding protein 39	-0.501462727	0.606544	0.410287685	5.97E-01	0.451316454	0.49858242	0.644737792	4.05E-01
P10321	HLA-C	major histocompatibility complex, class I, C	-0.502505462	0.606544	0.411140832	5.97E-01	0.452254916	0.49858242	0.646078451	4.05E-01
Q9H0B6	KLC2	kinesin light chain 2	0.753921033	0.606544	0.411229654	5.97E-01	0.45235262	0.49858242	0.646218028	4.05E-01
P45880	VDAC2	voltage dependent anion channel 2	-1.29390306	0.606544	-0.234194598	9.42E-01	0.021117263	0.993572665	0.647784783	5.42E-01
P22894	MMP8	matrix metallopeptidase 8	0.756273422	0.606544	0.412512775	5.97E-01	0.453764053	0.49858242	0.648234361	4.05E-01
O14713	ITGB1BP1	integrin subunit beta 1 binding protein 1	-0.505611421	0.606544	0.413682071	5.97E-01	0.455050279	0.49858242	0.650071826	4.05E-01
Q8NHG7	SVIP	small VCP interacting protein	-0.506732349	0.606544	0.414599195	5.97E-01	0.456059114	0.49858242	0.65151302	4.05E-01
Q9NUQ9	FAM49B	family with sequence similarity 49 member B	0.76086353	0.606544	0.415016471	5.97E-01	0.456518118	0.49858242	0.65216874	4.05E-01
Q9BRG1	VPS25	vacuolar protein sorting 25 homolog	-0.507600274	0.606544	0.415309315	5.97E-01	0.456840246	0.49858242	0.652628923	4.05E-01
P08F94	PKHD1	PKHD1 ciliary IPT domain containing fibrocystin/polyductin	0.763342914	0.606544	-1.145014371	5.97E-01	0.458005749	0.49858242	0.654293927	4.05E-01
O14964	HGS	hepatocyte growth factor-regulated tyrosine kinase substrate	-0.509769144	0.606544	0.417083845	5.97E-01	0.458792229	0.49858242	0.65541747	4.05E-01
Q01469	FABP5	fatty acid binding protein 5	-0.510152831	0.606544	-1.147843869	5.97E-01	-0.918275095	0.49858242	0.655910782	4.05E-01
P05023	ATP1A1	ATPase Na+/K+ transporting subunit alpha 1	-1.313332941	0.606544	-0.898023287	6.02E-01	-0.719441602	0.592511747	0.655972205	5.81E-01
P17612	PRKACA	protein kinase cAMP-activated catalytic subunit alpha	0.767044329	0.606544	0.418387816	5.97E-01	0.460226597	0.49858242	0.657466568	4.05E-01
P50991	CCT4	chaperonin containing TCP1 subunit 4	0.76764499	0.606544	0.418715449	5.97E-01	0.460586994	0.49858242	0.65798142	4.05E-01
Q9P0V3	SH3BP4	SH3 domain binding protein 4	-0.51248271	0.606544	0.419304035	5.97E-01	0.461234439	0.49858242	0.658906341	4.05E-01
Q13443	ADAM9	ADAM metallopeptidase domain 9	-0.120394411	0.9565129	-0.22102117	9.43E-01	-1.028142294	0.49858242	0.658982066	5.21E-01
Q9NSK0	KLC4	kinesin light chain 4	0.772017365	0.606544	0.421100381	5.97E-01	0.463210419	0.49858242	0.66172917	4.05E-01
Q8N335	GPD1L	glycerol-3-phosphate dehydrogenase 1 like	0.773020214	0.606544	0.42164739	5.97E-01	0.463812129	0.49858242	0.662588755	4.05E-01
Q9Y6U3	SCIN	scinderin	-0.516267566	0.606544	0.422400736	5.97E-01	0.464640809	0.49858242	0.663772585	4.05E-01
P07384	CAPN1	calpain 1	0.909068279	0.6325632	-0.397228106	9.14E-01	0.030088831	0.993572665	0.664167423	5.42E-01
P60022	DEFB1	defensin beta 1	-0.519338117	0.606544	0.424913005	5.97E-01	-0.93480861	0.49858242	0.667720436	4.05E-01
Q93077	HIST1H2AC	histone cluster 1 H2A family member c	0.102353798	0.9847168	0.446205388	9.37E-01	1.481747817	0.49858242	0.667836843	7.40E-01
P53801	PTTG1IP	PTTG1 interacting protein	-0.520082375	0.606544	0.425521943	5.97E-01	-0.936148275	0.49858242	0.668677339	4.05E-01
P35558	PCK1	phosphoenolpyruvate carboxykinase 1	0.780293465	0.606544	-1.170440197	5.97E-01	0.468176079	0.49858242	0.66882297	4.05E-01
P11117	ACP2	acid phosphatase 2, lysosomal	-0.520821681	0.606544	0.42612683	5.97E-01	0.468739513	0.49858242	0.669627875	4.05E-01
P54108	CRISP3	cysteine rich secretory protein 3	0.784485695	0.606544	0.427901288	5.97E-01	0.470691417	0.49858242	0.67241631	4.05E-01
Q12965	MYO1E	myosin IE	-0.523840285	0.606544	0.428596597	5.97E-01	0.471456256	0.49858242	0.673508938	4.05E-01
Q9BSQ5	CCM2	CCM2 scaffold protein	-0.524347075	0.606544	0.429011243	5.97E-01	0.471912368	0.49858242	0.674160525	4.05E-01
Q9BYG5	PARD6B	par-6 family cell polarity regulator beta	-0.524871302	0.606544	0.429440156	5.97E-01	0.472384172	0.49858242	0.674834531	4.05E-01
P01031	C5	complement C5	-0.532727336	0.9048944	2.527537199	8.35E-02	-1.702361622	0.49858242	0.674871099	6.79E-01
P30504	HLA-C	major histocompatibility complex, class I, C	-0.524960076	0.606544	0.42951279	5.97E-01	-0.944928137	0.49858242	0.674948669	4.05E-01
Q9H3G5	CPVL	carboxypeptidase vitellogenic like	-1.424863551	0.606544	-0.304067608	9.29E-01	0.063211589	0.983062223	0.677073448	5.60E-01
P04792	HSPB1	heat shock protein family B (small) member 1	-0.050579621	0.9688177	-0.56732551	5.97E-01	-0.229790855	0.715707085	0.679090193	2.18E-01
Q13113	PDZK1IP1	PDZK1 interacting protein 1	-1.49837221	0.606544	-0.535991867	9.08E-01	-1.339090709	0.516900608	0.681667277	6.95E-01
P11169	SLC2A3	solute carrier family 2 member 3	0.795635885	0.606544	0.43398321	5.97E-01	0.477381531	0.49858242	0.681973616	4.05E-01
P31939	ATIC	5-aminoimidazole-4-carboxamide ribonucleotide formyltrans	0.920377859	0.606544	-0.323465951	9.20E-01	0.090128595	0.963063137	0.685748406	5.06E-01
Q13018	PLA2R1	phospholipase A2 receptor 1	0.801949405	0.606544	-1.202924108	5.97E-01	0.481169643	0.49858242	0.687385205	4.05E-01
Q02790	FKBP4	FKBP prolyl isomerase 4	0.802828273	0.606544	0.437906331	5.97E-01	0.481696964	0.49858242	0.688138519	4.05E-01
P60903	S100A10	S100 calcium binding protein A10	-0.535926822	0.606544	-1.205835349	5.97E-01	-0.964668279	0.49858242	0.689048771	4.05E-01
P11216	PYGB	glycogen phosphorylase B	0.805477229	0.606544	0.439351216	5.97E-01	0.483286337	0.49858242	0.690409053	4.05E-01
P16885	PLCG2	phospholipase C gamma 2	-0.537417554	0.606544	0.439705271	5.97E-01	0.483675798	0.49858242	0.690965426	4.05E-01
P01593	IGKV1D-33	NA	-0.540013604	0.606544	0.441829312	5.97E-01	-0.972024487	0.49858242	0.694303205	4.05E-01
O60271	SPAG9	sperm associated antigen 9	-0.54107622	0.606544	0.442698725	5.97E-01	0.486968598	0.49858242	0.695669426	4.05E-01
P54578	USP14	ubiquitin specific peptidase 14	0.812888741	0.606544	0.443393859	5.97E-01	0.487733245	0.49858242	0.696761778	4.05E-01
P05186	ALPL	alkaline phosphatase, biomineralization associated	2.096759163	0.606544	-1.685908367	5.97E-01	1.258055498	0.49858242	0.696886778	5.42E-01
P30043	BLVRB	biliverdin reductase B	1.499371271	0.606544	-1.252343827	7.32E-01	2.254714271	0.241031753	0.700613676	6.37E-01
O60262	GNG7	G protein subunit gamma 7	-0.546298983	0.606544	0.446971895	5.97E-01	0.491669085	0.49858242	0.702384407	4.05E-01
O60884	DNAJA2	DnaJ heat shock protein family (Hsp40) member A2	-0.155494795	0.9487033	-0.358966764	9.20E-01	0.061166124	0.983062223	0.702592697	5.25E-01
P02652	APOA2	apolipoprotein A2	0.434124807	0.9223554	1.746965853	5.97E-01	1.965606581	0.49858242	0.703955036	6.67E-01
Q96CX2	KCTD12	potassium channel tetramerization domain containing 12	-0.548665879	0.606544	0.448908447	5.97E-01	0.493799291	0.49858242	0.705427559	4.05E-01
P00748	F12	coagulation factor XII	-0.549142786	0.606544	0.449298643	5.97E-01	-0.988457015	0.49858242	0.706040725	4.05E-01
Q495M3	SLC36A2	solute carrier family 36 member 2	-0.550302628	0.606544	0.450247605	5.97E-01	0.495272366	0.49858242	0.707531951	4.05E-01
Q7Z5L0	VMO1	vitelline membrane outer layer 1 homolog	0.827082486	0.606544	0.451135902	5.97E-01	0.496249492	0.49858242	0.708927846	4.05E-01
Q02156	PRKCE	protein kinase C epsilon	-0.551737548	0.606544	0.45142163	5.97E-01	0.496563793	0.49858242	0.709376847	4.05E-01
P43353	ALDH3B1	aldehyde dehydrogenase 3 family member B1	-0.264237935	0.9266018	-0.268232772	9.39E-01	0.012618913	0.99564488	0.709599667	5.49E-01
Q6UWP8	SBSN	suprabasin	-0.55456807	0.606544	-1.247778157	5.97E-01	-0.998222526	0.49858242	0.71301609	4.05E-01
Q8TF65	GIPC2	GIPC PDZ domain containing family member 2	0.832474101	0.606544	-1.248711152	5.97E-01	0.499484461	0.49858242	0.71354923	4.05E-01
P47895	ALDH1A3	aldehyde dehydrogenase 1 family member A3	0.836522071	0.606544	0.456284766	5.97E-01	0.501913242	0.49858242	0.717018918	4.05E-01
Q07507	DPT	dermatopontin	-0.55850096	0.606544	-1.256627161	5.97E-01	-1.005301729	0.49858242	0.718072663	4.05E-01
Q9BR76	CORO1B	coronin 1B	-0.047811051	0.9887973	1.297826777	5.97E-01	-1.193723498	0.507491123	0.718905419	5.84E-01
Q14449	GRB14	growth factor receptor bound protein 14	-0.559234897	0.606544	0.457555825	5.97E-01	0.503311408	0.49858242	0.719016297	4.05E-01
P31997	CEACAM8	carcinoembryonic antigen related cell adhesion molecule 8	0.841343254	0.606544	0.458914502	5.97E-01	0.504805952	0.49858242	0.72115136	4.05E-01
Q14624	ITIH4	inter-alpha-trypsin inhibitor heavy chain 4	0.802272811	0.8340657	1.815732813	5.97E-01	-0.731390239	0.768785785	0.721973427	6.57E-01
Q14204	DYNC1H1	dynein cytoplasmic 1 heavy chain 1	0.846091312	0.606544	0.461504352	5.97E-01	0.507654787	0.49858242	0.725221124	4.05E-01
Q04917	YWHAH	tyrosine 3-monooxygenase/tryptophan 5-monooxygenase ac	-0.564420906	0.606544	0.461798923	5.97E-01	0.507978815	0.49858242	0.725684022	4.05E-01
Q96KK5	HIST1H2AH	histone cluster 1 H2A family member h	1.742447973	0.606544	0.48157046	9.29E-01	1.458061806	0.49858242	0.726013918	7.18E-01
P52907	CAPZA1	capping actin protein of muscle Z-line subunit alpha 1	-0.566162947	0.606544	0.463224229	5.97E-01	0.509546652	0.49858242	0.727923788	4.05E-01
P35858	IGFALS	insulin like growth factor binding protein acid labile subunit	0.850728079	0.606544	0.464033498	5.97E-01	0.510436847	0.49858242	0.729195496	4.05E-01
Q9NS93	TM7SF3	transmembrane 7 superfamily member 3	-1.609960041	0.606544	1.317240034	5.97E-01	0.02038879	0.993572665	0.729551938	5.80E-01
P00352	ALDH1A1	aldehyde dehydrogenase 1 family member A1	0.948179268	0.6949271	-1.814457078	5.97E-01	1.431721548	0.49858242	0.730215772	5.77E-01
O14908	GIPC1	GIPC PDZ domain containing family member 1	-1.296264895	0.606544	1.060580369	5.97E-01	-1.118054534	0.49858242	0.734398637	4.92E-01
P50053	KHK	ketohexokinase	1.049457225	0.606544	-1.835836582	5.97E-01	1.205305974	0.49858242	0.735048318	5.10E-01
P34913	EPHX2	epoxide hydrolase 2	0.860454452	0.606544	0.469338792	5.97E-01	0.516272671	0.49858242	0.737532388	4.05E-01
Q06033	ITIH3	inter-alpha-trypsin inhibitor heavy chain 3	0.864163447	0.606544	0.47136188	5.97E-01	0.518498068	0.49858242	0.740711526	4.05E-01
Q15847	ADIRF	adipogenesis regulatory factor	-0.273048205	0.9311751	-1.937881877	5.97E-01	-1.455526952	0.49858242	0.74541061	5.71E-01
Q07837	SLC3A1	solute carrier family 3 member 1	-0.580297495	0.606544	0.474788859	5.97E-01	0.522267745	0.49858242	0.746096779	4.05E-01
P01871	IGHM	NA	-1.264498318	0.6304734	0.140423388	9.58E-01	-0.543988068	0.768785785	0.748104798	5.77E-01
Q05655	PRKCD	protein kinase C delta	-0.447939674	0.8871219	-0.067584326	9.81E-01	0.023111929	0.993572665	0.748201348	5.49E-01
P17213	BPI	bactericidal permeability increasing protein	-0.281707234	0.925356	-0.396811644	9.20E-01	-1.380557118	0.49858242	0.752143273	5.42E-01
P61626	LYZ	lysozyme	-0.012163687	0.9953954	0.473331395	8.76E-01	0.791961276	0.618655046	0.753241496	5.42E-01
Q9H2M3	BHMT2	betaine--homocysteine S-methyltransferase 2	0.883061228	0.606544	-1.324591841	5.97E-01	0.529836737	0.49858242	0.756909624	4.05E-01
A8MUH7	NA	NA	-0.590531233	0.606544	-1.328695273	5.97E-01	-1.062956219	0.49858242	0.759254442	4.05E-01
Q9UKN1	MUC12	mucin 12, cell surface associated	0.885821439	0.606544	0.48317533	5.97E-01	0.531492863	0.49858242	0.759275519	4.05E-01
Q14240	EIF4A2	eukaryotic translation initiation factor 4A2	1.054559853	0.652089	-0.193463118	9.55E-01	-0.010750942	0.99564488	0.760272987	5.60E-01
O43280	TREH	trehalase	0.888310747	0.606544	-1.332466121	5.97E-01	0.532986448	0.49858242	0.761409212	4.05E-01
P01620	NA	NA	0.892772241	0.606544	-1.339158361	5.97E-01	0.535663344	0.49858242	0.765233349	4.05E-01
P80511	S100A12	S100 calcium binding protein A12	1.912765037	0.606544	0.774374719	8.42E-01	1.384336488	0.49858242	0.766157794	6.92E-01
Q86T26	TMPRSS11B	transmembrane serine protease 11B	-0.597273731	0.606544	-1.343865894	5.97E-01	-1.075092715	0.49858242	0.767923368	4.05E-01
O60500	NPHS1	NPHS1 adhesion molecule, nephrin	0.896295881	0.606544	-1.344443822	5.97E-01	0.537777529	0.49858242	0.768253613	4.05E-01
P13688	CEACAM1	carcinoembryonic antigen related cell adhesion molecule 1	0.973162959	0.6546304	1.219516187	5.97E-01	-0.022158249	0.993572665	0.777437854	5.25E-01
P08575	PTPRC	protein tyrosine phosphatase receptor type C	0.906946405	0.6613763	1.14906479	5.97E-01	-0.031674898	0.993572665	0.780377317	4.99E-01

P01714	IGLV3-19	NA	0.912527606	0.606544	0.497742331	5.97E-01	0.547516564	0.49858242	0.78216652	4.05E-01
Q96F07	CYFIP2	cytoplasmic FMR1 interacting protein 2	-0.609440322	0.606544	0.49863299	5.97E-01	0.54849629	0.49858242	0.783566128	4.05E-01
P60033	CD81	CD81 molecule	0.915359281	0.606544	-1.373038921	5.97E-01	0.549215568	0.49858242	0.784593669	4.05E-01
A6NGU5	NA	NA	-0.61134611	0.606544	-1.375528747	5.97E-01	-1.100422997	0.49858242	0.786016427	4.05E-01
P50552	VASP	vasodilator stimulated phosphoprotein	1.054594375	0.606544	1.159777101	5.97E-01	0.118358076	0.955733699	0.789116931	4.99E-01
Q12923	PTPN13	protein tyrosine phosphatase non-receptor type 13	-0.615368671	0.606544	0.503483458	5.97E-01	0.553831804	0.49858242	0.791188291	4.05E-01
P04839	CYBB	cytochrome b-245 beta chain	1.304441659	0.606544	1.279013034	5.97E-01	0.283265521	0.881432248	0.792183807	5.45E-01
P54707	ATP12A	ATPase H+/K+ transporting non-gastric alpha2 subunit	-0.619478328	0.606544	0.506845905	5.97E-01	0.557530496	0.49858242	0.796472137	4.05E-01
P30492	HLA-B	major histocompatibility complex, class I, B	0.93323691	0.606544	-1.399855365	5.97E-01	0.559942146	0.49858242	0.799917351	4.05E-01
O00602	FCN1	ficolin 1	1.053300623	0.6655294	1.354767812	5.97E-01	-0.054631001	0.992706054	0.802393065	5.53E-01
O75828	CBR3	carbonyl reductase 3	0.945845718	0.606544	0.515915846	5.97E-01	0.567507431	0.49858242	0.810724902	4.05E-01
Q9HD89	RETN	resistin	-0.63125915	0.606544	-1.420333086	5.97E-01	-1.136266469	0.49858242	0.811618907	4.05E-01
P01857	IGHG1	NA	-0.453064725	0.9274558	0.979945702	8.17E-01	-0.213604557	0.949132218	0.812786381	6.53E-01
P05534	HLA-A	major histocompatibility complex, class I, A	-2.33674039	0.606544	-0.129771448	9.75E-01	-0.702550144	0.78099142	0.819398346	6.13E-01
P01624	IGKV3-15	NA	-0.64472345	0.606544	0.527501004	5.97E-01	-1.16050221	0.49858242	0.82893015	4.05E-01
P12814	ACTN1	actinin alpha 1	1.141942554	0.6325632	1.355141849	5.97E-01	1.490656033	0.49858242	0.834970314	5.42E-01
Q8IX04	UEVLD	UEV and lactate/malate dehyrogenase domains	0.07630759	0.9847168	-1.096170023	7.62E-01	0.915298583	0.610902087	0.835164864	5.51E-01
P01861	IGHG4	NA	1.93907766	0.606544	1.775828495	5.97E-01	1.953411344	0.49858242	0.839435979	6.53E-01
P80748	IGLV3-21	NA	1.406197656	0.606544	1.53750901	5.97E-01	0.16568554	0.954254821	0.840785745	5.84E-01
P07225	PROS1	protein S	-1.314935969	0.606544	1.94565636	3.50E-01	-1.983390114	0.49858242	0.848344952	5.39E-01
P04114	APOB	apolipoprotein B	-0.38968107	0.9371571	3.331230109	8.35E-02	-0.049995862	0.993572665	0.853024196	6.92E-01
P23527	HIST1H2BO	histone cluster 1 H2B family member o	-0.669478918	0.606544	0.547755479	5.97E-01	-1.205062053	0.49858242	0.860758609	4.05E-01
Q99571	P2RX4	purinergic receptor P2X 4	-0.147609965	0.9552645	1.200367803	5.97E-01	1.320404583	0.49858242	0.865521952	4.72E-01
P13671	C6	complement C6	0.850591701	0.7211264	1.259122162	5.97E-01	-0.189388466	0.942252124	0.868130495	4.96E-01
Q9UGT4	SUSD2	sushi domain containing 2	1.177652894	0.652089	-2.178356168	5.97E-01	1.612720755	0.49858242	0.885257732	5.45E-01
P19013	KRT4	keratin 4	-0.692625123	0.606544	0.566693282	5.97E-01	-1.246725221	0.49858242	0.890518015	4.05E-01
P21695	GPD1	glycerol-3-phosphate dehydrogenase 1	1.112049392	0.6385538	-2.036803385	5.97E-01	1.478434087	0.49858242	0.89455387	5.07E-01
P10314	HLA-A	major histocompatibility complex, class I, A	1.047582529	0.606544	-1.571373794	5.97E-01	0.628549518	0.49858242	0.897927882	4.05E-01
O60282	KIF5C	kinesin family member 5C	1.167857005	0.606544	-0.097096718	9.75E-01	0.168184658	0.941526432	0.899692675	4.79E-01
P61106	RAB14	RAB14, member RAS oncogene family	-0.188557764	0.9502692	1.40263211	5.97E-01	0.084161943	0.98183702	0.901695657	5.22E-01
Q08380	LGALS3BP	galectin 3 binding protein	-0.446548194	0.606544	0.375738642	5.97E-01	-0.562350414	0.49858242	0.908779931	5.55E-02
Q6W4X9	MUC6	mucin 6, oligomeric mucus/gel-forming	-0.716966159	0.606544	0.586608676	5.97E-01	-1.290539087	0.49858242	0.921813633	4.05E-01
P09525	ANXA4	annexin A4	-1.437566779	0.606544	-0.615889149	6.13E-01	-0.82767675	0.49858242	0.931092088	4.05E-01
P08311	CTSG	cathepsin G	1.878762429	0.606544	0.811566784	8.38E-01	1.314884654	0.549720269	0.932489777	6.23E-01
Q9H3R2	MUC13	mucin 13, cell surface associated	-0.465883071	0.9048944	1.424117779	5.97E-01	1.566529557	0.49858242	0.941893348	5.14E-01
P50990	CCT8	chaperonin containing TCP1 subunit 8	1.240496558	0.606544	-0.313193165	9.37E-01	0.223416819	0.914498633	0.947014658	4.57E-01
O75955	FLOT1	flotillin 1	-1.63762398	0.606544	-0.336470151	8.26E-01	-1.028507486	0.49858242	0.950930935	4.05E-01
Q93050	ATP6V0A1	ATPase H+ transporting V0 subunit a1	-0.186399511	0.9392316	-0.347210721	9.21E-01	1.267142265	0.49858242	0.954072575	4.10E-01
P29401	TKT	transketolase	0.556361787	0.8556187	0.127015526	9.74E-01	0.343225165	0.864476625	0.956974837	4.27E-01
P02671	FGA	fibrinogen alpha chain	-3.063755081	0.606544	1.58065724	7.78E-01	-1.180993641	0.705287042	0.961739754	6.72E-01
O60361	NA	NA	0.168570419	0.9321266	-0.513953308	8.26E-01	0.675557146	0.49858242	0.965081638	3.10E-01
Q03591	CFHR1	complement factor H related 1	1.128136687	0.7117516	1.615634505	5.97E-01	-0.203370742	0.952344606	0.981401898	5.45E-01
P05107	ITGB2	integrin subunit beta 2	1.776460828	0.606544	0.620966498	9.04E-01	1.372127175	0.5126016	0.983618119	5.97E-01
Q9H0W9	C11orf54	chromosome 11 open reading frame 54	0.06989309	0.9860434	-2.439533313	5.97E-01	0.898505884	0.627475017	0.992320533	4.84E-01
P08779	KRT16	keratin 16	-0.774222595	0.606544	0.633454851	5.97E-01	-1.393600672	0.49858242	0.995429051	4.05E-01
Q9H0U4	RAB1B	RAB1B, member RAS oncogene family	1.33755318	0.606544	-0.504566859	9.18E-01	0.132278272	0.963063137	0.996863968	5.06E-01
P13646	KRT13	keratin 13	-0.779530584	0.606544	0.63779775	5.97E-01	-1.403155051	0.49858242	1.002253608	4.05E-01
P01834	NA	NA	0.37018444	0.8588545	-0.409598815	7.07E-01	-0.276369921	0.800413265	1.00319548	2.84E-01
P19440	GGT1	gamma-glutamyltransferase 1	0.010985737	0.9953954	0.67388792	9.04E-01	1.054476657	0.676640473	1.003641104	5.29E-01
Q9UBP0	SPAST	spastin	0.338356823	0.9225917	-1.298969715	6.74E-01	-0.187688767	0.938574723	1.005212776	4.30E-01
P60660	MYL6	myosin light chain 6	-0.504450618	0.8524024	0.206157057	9.11E-01	-1.024823222	0.49858242	1.006768793	3.47E-01
Q96LD4	TRIM47	tripartite motif containing 47	-0.340815721	0.9189448	1.220073583	5.97E-01	1.342080941	0.49858242	1.006849204	4.19E-01
O60658	PDE8A	phosphodiesterase 8A	0.210095214	0.9225917	-0.578639672	8.19E-01	0.705750199	0.49858242	1.00821457	3.10E-01
P40121	CAPG	capping actin protein, gelsolin like	-1.791192038	0.606544	-1.73768297	5.97E-01	-1.797427453	0.49858242	1.011989697	4.68E-01
Q86YQ8	CPNE8	copine 8	-1.322680951	0.606544	0.450075182	9.21E-01	-0.501419741	0.854193535	1.019606423	5.40E-01
P19971	TYMP	thymidine phosphorylase	0.316295649	0.9048944	0.652589184	5.97E-01	-0.232679182	0.865530632	1.02549729	3.10E-01
P14780	MMP9	matrix metallopeptidase 9	-0.035814833	0.9928211	-0.010677689	9.97E-01	-0.029283654	0.993572665	1.030655165	4.38E-01
P31146	CORO1A	coronin 1A	2.742882144	0.606544	1.496117533	5.97E-01	1.645729286	0.49858242	1.032252967	4.98E-01
P61019	RAB2A	RAB2A, member RAS oncogene family	0.194107	0.9540881	0.492117287	9.04E-01	0.99289536	0.58859717	1.033132055	4.86E-01
Q9BUT1	BDH2	3-hydroxybutyrate dehydrogenase 2	0.98871238	0.6788966	-1.855122326	5.97E-01	1.411745691	0.49858242	1.033279568	4.23E-01
Q14247	CTTN	cortactin	-1.804706075	0.606544	2.21731627	8.35E-02	-2.500723646	0.496346254	1.034505689	4.30E-01
P04279	SEMG1	semenogelin 1	-0.815492644	0.606544	0.667221254	5.97E-01	-1.467886759	0.49858242	1.048490542	4.05E-01
P21266	GSTM3	glutathione S-transferase mu 3	0.143487867	0.9371571	-0.509803382	8.29E-01	0.7341502	0.49858242	1.048786	3.10E-01
P36969	GPX4	glutathione peroxidase 4	1.230684453	0.606544	-0.60486563	8.19E-01	0.738410672	0.49858242	1.054872388	3.10E-01
P35527	KRT9	keratin 9	-2.196596563	0.606544	2.587556994	5.97E-01	-2.488562706	0.49858242	1.057362982	5.49E-01
O75368	SH3BGRL	SH3 domain binding glutamate rich protein like	0.188486494	0.9311516	-0.56835315	8.26E-01	0.741463396	0.49858242	1.059233423	3.10E-01
P01009	SERPINA1	serpin family A member 1	-0.089416855	0.9826758	-0.811189029	6.74E-01	-0.494455183	0.784805924	1.065295083	4.05E-01
P49221	TGM4	transglutaminase 4	1.244459909	0.606544	-1.866689863	5.97E-01	0.746675945	0.49858242	1.066679922	4.05E-01
P07858	CTSB	cathepsin B	-1.672371339	0.606544	0.880969756	7.98E-01	-1.268987767	0.502679643	1.076056029	4.38E-01
Q02383	SEMG2	semenogelin 2	-0.837739391	0.606544	0.685423138	5.97E-01	-1.507930904	0.49858242	1.077093503	4.05E-01
P02794	FTH1	ferritin heavy chain 1	-1.594030756	0.606544	2.562940022	5.97E-01	-0.998060808	0.621660822	1.083323926	4.50E-01
P08118	MSMB	microseminoprotein beta	1.267477101	0.606544	-0.426628283	8.47E-01	0.760486261	0.49858242	1.086408944	3.10E-01
P11215	ITGAM	integrin subunit alpha M	1.802510433	0.606544	0.703626142	8.91E-01	1.327520262	0.567502564	1.087912845	5.71E-01
P08246	ELANE	elastase, neutrophil expressed	0.871214385	0.8556187	-1.131042055	8.26E-01	0.387513618	0.904141505	1.08809022	5.71E-01
P68402	PAFAH1B2	platelet activating factor acetylhydrolase 1b catalytic subunit	1.274226916	0.606544	-0.591683787	8.24E-01	0.76453615	0.49858242	1.092194499	3.10E-01
P00918	CA2	carbonic anhydrase 2	0.759704735	0.8768591	-0.97150724	8.41E-01	1.337594329	0.501877688	1.093451404	5.42E-01
P09936	UCHL1	ubiquitin C-terminal hydrolase L1	0.184655997	0.9341569	-0.575057251	8.26E-01	0.76655476	0.49858242	1.095078229	3.10E-01
P20073	ANXA7	annexin A7	0.258206689	0.9189448	-0.446222965	7.62E-01	-0.52233428	0.630259159	1.099087785	3.16E-01
P61158	ACTR3	actin related protein 3	0.199768795	0.9311092	0.700233984	5.97E-01	0.770257382	0.49858242	1.100367688	3.10E-01
O43291	SPINT2	serine peptidase inhibitor, Kunitz type 2	0.260331137	0.9189448	-0.669691654	8.17E-01	-0.335184426	0.835588099	1.100610812	3.10E-01
Q12765	SCRN1	secernin 1	1.294517297	0.606544	-0.652642319	8.17E-01	0.776710378	0.49858242	1.109586254	3.10E-01
P07148	FABP1	fatty acid binding protein 1	0.210942685	0.9274558	-0.615003998	8.23E-01	0.783463546	0.49858242	1.119233638	3.10E-01
Q9NQA5	TRPV5	transient receptor potential cation channel subfamily V mem	-0.597447887	0.7649939	0.095738625	9.74E-01	-0.652596503	0.697971465	1.124650861	4.04E-01
Q7L5L3	GDPD3	glycerophosphodiester phosphodiesterase domain containin	0.126930887	0.9501086	-0.514122683	8.33E-01	0.788356508	0.49858242	1.126223583	3.10E-01
P03951	F11	coagulation factor XI	-0.876446701	0.606544	-0.703942198	8.17E-01	-1.577604062	0.49858242	1.126860044	3.10E-01
P78417	GSTO1	glutathione S-transferase omega 1	1.317818745	0.606544	-0.584699612	8.26E-01	0.790691247	0.49858242	1.129558924	3.10E-01
P02765	AHSG	alpha 2-HS glycoprotein	0.391567545	0.9326901	-3.306262627	7.10E-02	-1.084817307	0.703381751	1.129790893	5.42E-01
Q9BZV1	UBXN6	UBX domain protein 6	0.086104757	0.9687687	-0.46788301	8.42E-01	0.796859779	0.49858242	1.138371112	3.10E-01
Q99961	SH3GL1	SH3 domain containing GRB2 like 1, endophilin A2	0.169796347	0.9371571	0.725518109	5.97E-01	0.79806992	0.49858242	1.140099886	3.10E-01
Q7Z2W4	ZC3HAV1	zinc finger CCCH-type containing, antiviral 1	-0.013817609	0.9949121	-0.34588671	9.00E-01	0.798258308	0.49858242	1.140369011	3.15E-01
Q9UBD6	RHCG	Rh family C glycoprotein	0.287473372	0.9189448	-1.998957763	5.97E-01	-0.329195242	0.838926923	1.142261579	3.10E-01
Q99828	CIB1	calcium and integrin binding 1	0.350110044	0.9048944	-0.479667121	8.41E-01	-0.262061883	0.864887297	1.143179877	3.10E-01
Q6IWH7	ANO7	anoctamin 7	1.334216917	0.606544	-0.533800191	8.31E-01	0.80053015	0.49858242	1.1436145	3.10E-01
Q7Z404	TMC4	transmembrane channel like 4	1.334431435	0.606544	-0.542086734	8.31E-01	0.800658861	0.49858242	1.143798373	3.10E-01
O43511	SLC26A4	solute carrier family 26 member 4	0.16226802	0.9371571	0.728051831	5.97E-01	0.800857014	0.49858242	1.144081449	3.10E-01
P50995	ANXA11	annexin A11	0.021495061	0.9925048	-0.478121038	5.97E-01	-0.248089635	0.83794346	1.144803072	1.70E-01
P61160	ACTR2	actin related protein 2	-0.674551841	0.8332014	1.687866341	5.97E-01	-0.9845865	0.655818184	1.148590885	4.05E-01
Q7L9L4	MOB1B	MOB kinase activator 1B	0.222565511	0.9266018	-0.639606523	8.20E-01	0.806207471	0.49858242	1.151724958	3.10E-01
P62942	FKBP1A	FKBP prolyl isomerase 1A	0.244096278	0.9225917	0.734388013	5.97E-01	-0.382637471	0.81904282	1.154038306	3.10E-01
P0CG48	UBC	ubiquitin C	-0.295116189	0.9036023	-0.587213223	5.97E-01	-0.145451724	0.867184582	1.157587256	1.10E-01
Q96C24	SYTL4	synaptotagmin like 4	1.355811414	0.606544	-0.461530133	8.47E-01	0.813486849	0.49858242	1.16212407	3.10E-01
O75363	BCAS1	breast carcinoma amplified sequence 1	-0.59154574	0.853127	0.178763731	9.55E-01	-2.039455049	0.49858242	1.165198855	4.05E-01
O75636	FCN3	ficolin 3	0.009794537	0.9953954	0.74330156	5.97E-01	-0.643527272	0.721528858	1.168045308	3.14E-01
Q9Y617	PSAT1	phosphoserine aminotransferase 1	0.118461305	0.9540881	-0.51858663	8.38E-01	0.821045062	0.49858242	1.172921517	3.10E-01
P30046	DDT	D-dopachrome tautomerase	1.371690646	0.606544	-0.669552882	8.19E-01	0.823014388	0.49858242	1.17573484	3.10E-01
O95865	DDAH2	dimethylarginine dimethylaminohydrolase 2	0.282481301	0.9189448	0.749276161	5.97E-01	0.824203777	0.49858242	1.177433967	3.10E-01
A8MQ03	CYSRT1	cysteine rich tail 1	-0.917075413	0.606544	-0.692666016	8.17E-01	-1.650735743	0.49858242	1.179096959	3.10E-01
P07357	C8A	complement C8 alpha chain	0.186388508	0.9371571	0.757678604	5.97E-01	-0.465457582	0.793543145	1.190637806	3.10E-01
P01623	NA	NA	-0.210629398	0.9540881	0.219655569	9.55E-01	-0.134415967	0.963063137	1.192699567	4.24E-01
P36873	PPP1CC	protein phosphatase 1 catalytic subunit gamma	0.213180861	0.9311516	0.759645153	5.97E-01	-0.438252405	0.800288577	1.193728098	3.10E-01
P0C0L4	C4A	complement C4A (Rodgers blood group)	-0.769840276	0.8814327	-1.173874284	5.97E-01	-0.957356547	0.735299939	1.193847952	5.09E-01
O95394	PGM3	phosphoglucomutase 3	1.393129607	0.606544	-0.4414764	8.61E-01	0.835877764	0.49858242	1.194111092	3.10E-01
P63096	GNAI1	G protein subunit alpha i1	-0.107659238	0.9828123	-1.161448453	8.17E-01	-0.534980514	0.860197922	1.198177079	5.09E-01
P09417	QDPR	quinoid dihydropteridine reductase	1.402230638	0.606544	-0.651506723	8.24E-01	0.841338383	0.49858242	1.201911975	3.10E-01
A6NEC2	NA	NA	1.403156458	0.606544	-0.698417519	8.19E-01	0.841893875	0.49858242	1.202705535	3.10E-01
P32119	PRDX2	peroxiredoxin 2	2.22011471	0.606544	-0.179002484	9.75E-01	1.807886213	0.49858242	1.203140176	5.07E-01
P21964	COMT	catechol-O-methyltransferase	0.217737217	0.9371571	0.284914814	9.27E-01	0.346409538	0.862480694	1.204381881	4.05E-01
P48637	GSS	glutathione synthetase	1.409306984	0.606544	-0.682788728	8.19E-01	0.845584191	0.49858242	1.207977415	3.10E-01
P10599	TXN	thioredoxin	1.182708874	0.606544	-1.042846152	6.56E-01	1.514504204	0.496346254	1.210259006	4.05E-01
P52566	ARHGDIB	Rho GDP dissociation inhibitor beta	-0.567518111	0.8469954	0.249979037	9.38E-01	-1.931738749	0.49858242	1.210766574	4.05E-01
P17342	NPR3	natriuretic peptide receptor 3	1.41310325	0.606544	-0.685271548	8.19E-01	0.84786195	0.49858242	1.211231357	3.10E-01
P15153	RAC2	Rac family small GTPase 2	1.658761114	0.606544	0.824772145	8.31E-01	1.065662516	0.65994855	1.211863534	4.98E-01
O14745	SLC9A3R1	SLC9A3 regulator 1	-1.339657753	0.606544	-0.584448425	6.94E-01	-1.513742795	0.49858242	1.213104924	3.33E-01
P31689	DNAJA1	DnaJ heat shock protein family (Hsp40) member A1	0.282994878	0.9189448	-0.734201318	8.17E-01	0.851156728	0.49858242	1.215938182	3.10E-01
P03973	SLPI	secretory leukocyte peptidase inhibitor	-0.822351096	0.7117516	-1.489020302	5.97E-01	-0.84183933	0.685038046	1.218953316	4.05E-01
Q13563	PKD2	polycystin 2, transient receptor potential cation channel	0.367793855	0.9086495	-0.842117218	8.04E-01	-0.290474212	0.861815025	1.228020671	3.10E-01
P02748	C9	complement C9	0.590849836	0.9189448	1.779391681	5.97E-01	-0.927746857	0.745922129	1.228825034	5.05E-01
P05413	FABP3	fatty acid binding protein 3	0.180178334	0.9371571	-0.612248621	8.26E-01	0.860465465	0.49858242	1.229236379	3.10E-01
P36543	ATP6V1E1	ATPase H+ transporting V1 subunit E1	0.187891633	0.9371571	-0.621783445	8.26E-01	0.860616171	0.49858242	1.229451672	3.10E-01

Q9Y490	TLN1	talin 1	0.570508218	0.8539819	0.0577761	9.83E-01	1.619602658	0.496346254	1.230147715	4.05E-01
P36405	ARL3	ADP ribosylation factor like GTPase 3	0.276493272	0.9223554	-0.730934733	8.17E-01	0.861524579	0.49858242	1.230749399	3.10E-01
Q8WVN6	SECTM1	secreted and transmembrane 1	0.134970581	0.9502692	-0.814701312	8.12E-01	-0.544396078	0.768785785	1.23243626	3.10E-01
P30153	PPP2R1A	protein phosphatase 2 scaffold subunit Aalpha	1.439888243	0.606544	-0.33856574	9.06E-01	0.863932946	0.49858242	1.234189923	3.15E-01
P28907	CD38	CD38 molecule	1.442124218	0.606544	-0.578529922	8.31E-01	0.865274531	0.49858242	1.236106473	3.10E-01
P13796	LCP1	lymphocyte cytosolic protein 1	1.444075645	0.606544	-0.617480644	8.26E-01	0.866445387	0.49858242	1.237779124	3.10E-01
P26038	MSN	moesin	-0.598457605	0.6660033	-0.406254595	6.96E-01	-0.698044783	0.49858242	1.238552715	1.10E-01
Q14344	GNA13	G protein subunit alpha 13	-0.887207571	0.606544	-0.50364753	6.66E-01	-0.977552428	0.49858242	1.239808356	1.87E-01
P31947	SFN	stratifin	-1.678932384	0.606544	-1.329242347	5.97E-01	-1.273643166	0.49858242	1.240698303	4.05E-01
Q8N4C8	MINK1	misshapen like kinase 1	0.148008815	0.9478026	-0.576600792	8.31E-01	0.868897941	0.49858242	1.241282773	3.10E-01
P52788	SMS	spermine synthase	1.450189888	0.606544	-0.539962025	8.38E-01	0.870113933	0.49858242	1.243019904	3.10E-01
P98160	HSPG2	heparan sulfate proteoglycan 2	0.290075014	0.9189448	-0.63472647	8.26E-01	-0.384191232	0.835127935	1.245486155	3.10E-01
Q15485	FCN2	ficolin 2	0.122196036	0.9553457	0.794596443	5.97E-01	-0.567273151	0.767371774	1.248651554	3.10E-01
P43490	NAMPT	nicotinamide phosphoribosyltransferase	1.46678686	0.606544	0.80006556	5.97E-01	0.880072116	0.49858242	1.25724588	3.10E-01
Q9NUP9	LIN7C	lin-7 homolog C, crumbs cell polarity complex component	-1.928546744	0.606544	0.269952098	9.37E-01	-2.037143313	0.49858242	1.260251399	4.05E-01
Q9NZM1	MYOF	myoferlin	-1.685139077	0.606544	0.543749168	9.04E-01	-2.001797494	0.49858242	1.263289088	4.10E-01
Q9UBQ7	GRHPR	glyoxylate and hydroxypyruvate reductase	1.477339578	0.606544	-0.753492867	8.17E-01	0.886403747	0.49858242	1.266291067	3.10E-01
P11586	MTHFD1	methylenetetrahydrofolate dehydrogenase, cyclohydrolase a	0.249753802	0.9254988	-0.710334709	8.20E-01	0.888401094	0.49858242	1.26914442	3.10E-01
O75351	VPS4B	vacuolar protein sorting 4 homolog B	-0.582066643	0.8271667	-1.089040211	5.97E-01	-0.386399436	0.738055058	1.270170112	2.49E-01
P02751	FN1	fibronectin 1	-0.728697474	0.8925184	0.57946287	8.81E-01	0.587199288	0.851349271	1.272596247	4.62E-01
Q5TFQ8	SIRPB1	signal regulatory protein beta 1	0.213207589	0.9341569	-2.227484488	5.97E-01	-0.48253084	0.796117674	1.272848279	3.10E-01
P08294	SOD3	superoxide dismutase 3	-0.996622381	0.606544	-0.956702399	7.85E-01	-1.793920285	0.49858242	1.281371632	3.10E-01
Q8N9U0	TC2N	tandem C2 domains, nuclear	1.495650242	0.606544	-0.535440631	8.41E-01	0.897390145	0.49858242	1.281985922	3.10E-01
Q687X5	STEAP4	STEAP4 metalloreductase	1.497072818	0.606544	-0.610567954	8.31E-01	0.898243691	0.49858242	1.283205273	3.10E-01
Q9Y5S2	CDC42BPB	CDC42 binding protein kinase beta	-0.998735616	0.606544	0.817147322	5.97E-01	-0.168512803	0.929712425	1.284088649	3.15E-01
P55072	VCP	valosin containing protein	2.421908069	0.606544	-1.147290202	8.12E-01	2.805405005	0.069521741	1.288157497	4.05E-01
Q14914	PTGR1	prostaglandin reductase 1	0.539155787	0.8795112	-2.879054137	5.97E-01	0.480653114	0.807619793	1.293370177	4.05E-01
P02760	AMBP	alpha-1-microglobulin/bikunin precursor	0.928237491	0.822321	-2.715381805	5.37E-01	-0.911238348	0.703381751	1.296340242	4.50E-01
P11274	BCR	BCR activator of RhoGEF and GTPase	-1.008311643	0.606544	-0.381470568	9.04E-01	-0.154198004	0.938574723	1.296400684	3.15E-01
O95498	VNN2	vanin 2	0.423572938	0.8968647	0.825338163	5.97E-01	-0.26883881	0.878195	1.29695997	3.10E-01
P07911	UMOD	uromodulin	-1.695797406	0.606544	-0.723294988	8.04E-01	-1.191152414	0.49858242	1.29810752	4.05E-01
Q9NZH0	GPRC5B	G protein-coupled receptor class C group 5 member B	-0.619348908	0.6253347	-0.530024615	7.78E-01	-0.580735574	0.49858242	1.29999425	7.88E-02
Q9UHI7	SLC23A1	solute carrier family 23 member 1	-0.251068441	0.9371571	-0.805999831	8.26E-01	-0.860320341	0.64370328	1.302521935	4.05E-01
Q8TAD7	C12orf75	chromosome 12 open reading frame 75	0.253371979	0.9266018	-0.726899842	8.20E-01	0.915075309	0.49858242	1.307250442	3.10E-01
Q9Y696	CLIC4	chloride intracellular channel 4	-0.989574402	0.7849608	-1.16451059	7.74E-01	-1.634527287	0.49858242	1.308226182	4.40E-01
Q9BWD1	ACAT2	acetyl-CoA acetyltransferase 2	1.530889258	0.606544	-0.674296238	8.26E-01	0.918533555	0.49858242	1.312190793	3.10E-01
Q3SXY8	ARL13B	ADP ribosylation factor like GTPase 13B	0.426788701	0.8968647	-0.942406865	7.94E-01	0.920965612	0.49858242	1.31566516	3.10E-01
P80297	MT1X	metallothionein 1X	1.539839444	0.606544	-0.813762399	8.17E-01	0.923903667	0.49858242	1.319862381	3.10E-01
Q6UXY8	TMC5	transmembrane channel like 5	0.326340539	0.9189448	-0.651901681	8.27E-01	-0.388449227	0.83794346	1.323030374	3.10E-01
P01911	HLA-DRB1	major histocompatibility complex, class II, DR beta 1	-0.030248627	0.9925048	0.842008299	5.97E-01	0.926209129	0.49858242	1.323155899	3.15E-01
Q4U2R8	SLC22A6	solute carrier family 22 member 6	0.3182995	0.9189448	-0.8125196	8.17E-01	0.928134471	0.49858242	1.325906387	3.10E-01
Q9H190	SDCBP2	syndecan binding protein 2	0.1772951	0.939106	-0.640081886	8.31E-01	0.929483379	0.49858242	1.327833398	3.10E-01
P51148	RAB5C	RAB5C, member RAS oncogene family	-0.75376016	0.7305966	-0.680068409	5.97E-01	-0.565718498	0.523405303	1.334122929	2.09E-01
Q13938	CAPS	calcyphosine	-0.369039968	0.9225917	-0.605875773	8.80E-01	-2.510041645	0.498467678	1.334981728	4.05E-01
Q96IU4	ABHD14B	abhydrolase domain containing 14B	0.63131686	0.8340657	-0.187994946	9.55E-01	1.604422732	0.496346254	1.336738913	3.65E-01
P14555	PLA2G2A	phospholipase A2 group IIA	0.359572597	0.9189448	0.852242406	5.97E-01	-0.361634912	0.843844089	1.339238066	3.10E-01
P30039	PBLD	phenazine biosynthesis like protein domain containing	0.322046923	0.9189448	-0.821648862	8.17E-01	0.938100805	0.49858242	1.340144007	3.10E-01
P22732	SLC2A5	solute carrier family 2 member 5	-0.083050235	0.9828123	-2.239795546	5.97E-01	-2.025578701	0.49858242	1.343526949	4.05E-01
Q96CF2	CHMP4C	charged multivesicular body protein 4C	-1.47056078	0.606544	-1.132665274	6.94E-01	-2.791472605	0.076861408	1.344012771	4.05E-01
Q6ZS17	RIPOR1	RHO family interacting cell polarization regulator 1	0.162469232	0.9476282	-0.62883732	8.31E-01	0.944775178	0.49858242	1.349678826	3.10E-01
P61769	B2M	beta-2-microglobulin	0.880302272	0.7268602	-1.083540457	6.64E-01	0.707763069	0.750706799	1.352501797	4.05E-01
P02774	GC	GC vitamin D binding protein	1.583360376	0.606544	0.863651114	5.97E-01	0.950016226	0.49858242	1.357166037	3.10E-01
P05164	MPO	myeloperoxidase	1.687385498	0.7132478	0.977053997	8.91E-01	0.96256882	0.78099142	1.357272149	5.71E-01
P13489	RNH1	ribonuclease/angiogenin inhibitor 1	1.588186839	0.606544	-0.703524261	8.26E-01	0.952912103	0.49858242	1.361303005	3.10E-01
Q9H4A4	RNPEP	arginyl aminopeptidase	1.591784805	0.606544	-0.629987648	8.31E-01	0.955070883	0.49858242	1.364386976	3.10E-01
O75841	UPK1B	uroplakin 1B	-2.169198985	0.606544	-0.709073215	8.80E-01	-3.6420444	0.241031753	1.368104102	4.05E-01
P02790	HPX	hemopexin	-1.545892033	0.606544	0.917520293	8.24E-01	-0.882974071	0.657624868	1.372631613	4.05E-01
P01860	NA	NA	0.577082552	0.9214958	2.839644754	3.55E-01	-1.875638228	0.49858242	1.372878861	4.99E-01
Q9NQR4	NIT2	nitrilase family member 2	1.602871074	0.606544	-0.840138157	8.17E-01	0.961722644	0.49858242	1.373889492	3.10E-01
Q14314	FGL2	fibrinogen like 2	0.353592692	0.9189448	-0.660375356	8.31E-01	-0.388528229	0.838926923	1.375729172	3.10E-01
Q9H444	CHMP4B	charged multivesicular body protein 4B	-0.946583336	0.6949271	-0.365477357	8.99E-01	-1.049765636	0.49858242	1.376070904	3.10E-01
Q8WTX9	ZDHHC1	zinc finger DHHC-type containing 1	0.525856932	0.8841371	-1.426800043	5.97E-01	0.445800211	0.828728139	1.379239195	3.90E-01
P09960	LTA4H	leukotriene A4 hydrolase	1.611883239	0.606544	-0.703482655	8.26E-01	0.967129944	0.49858242	1.381614205	3.10E-01
Q9BV36	MLPH	melanophilin	1.612354821	0.606544	-0.447151479	8.90E-01	0.967412893	0.49858242	1.382018418	3.14E-01
P14550	AKR1A1	aldo-keto reductase family 1 member A1	-0.217934564	0.9540881	0.247307396	9.55E-01	-0.890410123	0.667229087	1.382332093	4.05E-01
P20151	KLK2	kallikrein related peptidase 2	0.230228195	0.9341569	0.879984444	5.97E-01	-0.525739859	0.796117674	1.382832697	3.10E-01
Q9NXU5	ARL15	ADP ribosylation factor like GTPase 15	0.286459506	0.9225917	-0.792623841	8.19E-01	0.970331783	0.49858242	1.386188261	3.10E-01
P49189	ALDH9A1	aldehyde dehydrogenase 9 family member A1	1.618602317	0.606544	-0.599470766	8.38E-01	0.97116139	0.49858242	1.387373414	3.10E-01
P10643	C7	complement C7	0.368094037	0.9225917	0.326414615	9.24E-01	-1.085118008	0.621660822	1.388500435	4.05E-01
P13987	CD59	CD59 molecule (CD59 blood group)	-1.157949815	0.606544	-0.513079551	7.62E-01	-1.019990817	0.49858242	1.389739153	2.46E-01
Q14002	CEACAM7	carcinoembryonic antigen related cell adhesion molecule 7	-1.081853375	0.606544	-0.645752966	8.31E-01	-1.947336074	0.49858242	1.390954339	3.10E-01
P08263	GSTA1	glutathione S-transferase alpha 1	0.985885469	0.7016777	-1.718542166	5.97E-01	1.149249523	0.515600196	1.391519809	4.05E-01
P14618	PKM	pyruvate kinase M1/2	-0.627972415	0.8524024	-0.249970252	9.04E-01	-0.387818406	0.768785785	1.392604651	2.84E-01
P02763	ORM1	orosomucoid 1	1.624779446	0.606544	0.886243334	5.97E-01	0.974867668	0.49858242	1.392668096	3.10E-01
O43451	MGAM	maltase-glucoamylase	-0.699078	0.8035497	-1.015238948	5.97E-01	-0.951750165	0.49858242	1.401697628	2.42E-01
O95630	STAMBP	STAM binding protein	-0.534150675	0.8539819	-0.077004698	9.81E-01	-0.437549915	0.825108673	1.404263659	3.33E-01
O00159	MYO1C	myosin IC	-2.467399506	0.606544	-1.337060016	5.97E-01	-1.758972486	0.49858242	1.407455096	4.05E-01
P06731	CEACAM5	carcinoembryonic antigen related cell adhesion molecule 5	0.221195676	0.9371571	-2.465387296	5.97E-01	-0.550032605	0.793543145	1.408792741	3.10E-01
Q9NR45	NANS	N-acetylneuraminate synthase	1.644174165	0.606544	-0.70583109	8.26E-01	0.986504499	0.49858242	1.409292142	3.10E-01
P35237	SERPINB6	serpin family B member 6	1.657862587	0.606544	-0.711130885	8.26E-01	0.994717552	0.49858242	1.421025075	3.10E-01
P63027	VAMP2	vesicle associated membrane protein 2	1.660710659	0.606544	-0.753203487	8.26E-01	0.996426395	0.49858242	1.423466279	3.10E-01
O75110	ATP9A	ATPase phospholipid transporting 9A (putative)	1.676717778	0.606544	-0.704496165	8.28E-01	1.006030667	0.49858242	1.437186667	3.10E-01
Q3LXA3	TKFC	triokinase and FMN cyclase	-0.25799521	0.9423969	0.104840114	9.81E-01	-0.686988948	0.744892198	1.437362888	4.05E-01
Q9NQX5	NPDC1	neural proliferation, differentiation and control 1	1.679556812	0.606544	-0.621895817	8.38E-01	1.007734087	0.49858242	1.439620125	3.10E-01
P05062	ALDOB	aldolase, fructose-bisphosphate B	-0.618519802	0.8556187	-0.496941506	8.38E-01	-0.39346639	0.796117674	1.443239004	3.10E-01
Q9NP79	VTA1	vesicle trafficking 1	-0.870854298	0.6699589	-1.205928968	5.97E-01	-0.76638345	0.49858242	1.447340252	1.78E-01
Q15185	PTGES3	prostaglandin E synthase 3	-0.164950128	0.9371571	-0.259723522	9.24E-01	-0.021836562	0.993572665	1.452510135	1.70E-01
Q8N3R9	MPP5	membrane palmitoylated protein 5	0.33045209	0.9214958	-0.869212506	8.17E-01	1.02004687	0.49858242	1.457209815	3.10E-01
P08473	MME	membrane metalloendopeptidase	0.069583401	0.9752257	-0.572037863	7.71E-01	-0.27018179	0.768785785	1.461486378	6.74E-02
P15121	AKR1B1	aldo-keto reductase family 1 member B	0.326017947	0.9225917	-0.867388315	8.17E-01	1.028005835	0.49858242	1.468579765	3.10E-01
P04003	C4BPA	complement component 4 binding protein alpha	-2.574405734	0.606544	2.439484496	5.97E-01	-2.505029767	0.49858242	1.469504023	4.22E-01
Q9HCH3	CPNE5	copine 5	0.193586203	0.9392316	-2.583725086	5.97E-01	-0.617718929	0.778956299	1.476414335	3.10E-01
P31150	GDI1	GDP dissociation inhibitor 1	1.722588629	0.606544	-0.834630485	8.19E-01	1.033553178	0.49858242	1.476504539	3.10E-01
Q9BYF1	ACE2	angiotensin I converting enzyme 2	-0.717503054	0.8556187	-1.253701857	6.96E-01	-1.152957608	0.557024507	1.476561212	4.05E-01
O60701	UGDH	UDP-glucose 6-dehydrogenase	1.727002761	0.606544	-0.746691815	8.26E-01	1.036201657	0.49858242	1.480288081	3.10E-01
P53990	IST1	IST1 factor associated with ESCRT-III	-1.20317835	0.606544	-0.948551112	5.97E-01	-1.181244208	0.49858242	1.481374935	2.87E-01
O43488	AKR7A2	aldo-keto reductase family 7 member A2	1.685584921	0.606544	-1.40544768	5.97E-01	1.771988823	0.496346254	1.48727147	3.54E-01
P11766	ADH5	alcohol dehydrogenase 5 (class III), chi polypeptide	0.684267447	0.8271667	-0.127321061	9.74E-01	0.644444211	0.705410918	1.488125629	3.26E-01
P51149	RAB7A	RAB7A, member RAS oncogene family	0.534964381	0.8968647	0.309257528	9.43E-01	0.819036631	0.725716007	1.494273317	4.05E-01
Q9Y3R5	DOP1B	DOP1 leucine zipper like protein B	1.744699285	0.606544	-0.650906277	8.38E-01	1.046819571	0.49858242	1.49545653	3.10E-01
Q14108	SCARB2	scavenger receptor class B member 2	-1.856901918	0.606544	0.920743654	7.62E-01	-1.134453348	0.537759113	1.501026965	3.90E-01
Q09666	AHNAK	AHNAK nucleoprotein	-1.059268915	0.6956246	-0.152360836	9.75E-01	-2.898102819	0.49858242	1.501879143	4.05E-01
P04899	GNAI2	G protein subunit alpha i2	-0.84148814	0.7117516	-0.604003941	6.21E-01	-0.752289159	0.49858242	1.50758956	1.70E-01
O95398	RAPGEF3	Rap guanine nucleotide exchange factor 3	0.413171181	0.9189448	-3.042829243	5.97E-01	0.464283627	0.838926923	1.512510111	4.05E-01
P08238	HSP90AB1	heat shock protein 90 alpha family class B member 1	1.904139928	0.606544	-0.295831096	9.55E-01	0.633534166	0.83794346	1.514588326	4.09E-01
P28838	LAP3	leucine aminopeptidase 3	1.799687493	0.606544	-1.377005362	6.16E-01	1.824589653	0.496346254	1.52838528	3.54E-01
Q9H7P6	MVB12B	multivesicular body subunit 12B	0.187845177	0.9467495	-0.718427236	8.31E-01	1.073357941	0.49858242	1.533368487	3.10E-01
Q9HC84	MUC5B	mucin 5B, oligomeric mucus/gel-forming	0.090024362	0.9790738	0.980725912	5.97E-01	1.078798503	0.49858242	1.541140718	3.10E-01
P60953	CDC42	cell division cycle 42	-0.771657808	0.7360888	-0.756705743	5.97E-01	-0.551399774	0.561248865	1.55356932	1.67E-01
O00299	CLIC1	chloride intracellular channel 1	-0.855873431	0.7016777	-0.248638964	9.04E-01	-0.892309261	0.49858242	1.563033398	1.70E-01
P51178	PLCD1	phospholipase C delta 1	-0.511056014	0.9189448	-2.344104642	5.97E-01	-1.203934298	0.600120045	1.572214572	4.05E-01
P24158	PRTN3	proteinase 3	0.89107676	0.6086317	1.008959533	5.97E-01	0.074478512	0.972684176	1.585507838	1.70E-01
P08134	RHOC	ras homolog family member C	0.391105068	0.9341569	0.397939836	9.43E-01	2.865870827	0.241031753	1.587460773	4.05E-01
Q9Y6R1	SLC4A4	solute carrier family 4 member 4	0.514811167	0.8968647	-1.137426244	7.94E-01	1.112347587	0.49858242	1.589067981	3.10E-01
P04075	ALDOA	aldolase, fructose-bisphosphate A	-0.399027864	0.9189448	-0.798474399	5.97E-01	-0.19469088	0.878195	1.59150628	1.70E-01
Q8WV92	MITD1	microtubule interacting and trafficking domain containing 1	-0.570774913	0.8556187	-1.237815375	5.97E-01	-1.230024842	0.49858242	1.593471405	2.46E-01
P01876	IGHA1	NA	-0.769616668	0.8469954	-0.3056606	9.04E-01	-1.413775862	0.49858242	1.597028644	3.10E-01
O43795	MYO1B	myosin IB	-0.697540282	0.8111007	0.021317805	9.97E-01	-0.700428149	0.753113555	1.59985462	3.26E-01
Q96PD5	PGLYRP2	peptidoglycan recognition protein 2	0.867584858	0.6325632	-0.426203611	9.04E-01	0.036560873	0.993520587	1.607912095	1.70E-01
O95497	VNN1	vanin 1	-0.175459195	0.9371571	-1.525096885	5.97E-01	0.05206281	0.985484317	1.615963265	1.70E-01
Q16775	HAGH	hydroxyacylglutathione hydrolase	1.08194612	0.606544	-0.997103017	7.43E-01	-0.356323144	0.86110477	1.627904089	2.66E-01
P55017	SLC12A3	solute carrier family 12 member 3	0.709247408	0.8968647	-0.541425248	9.21E-01	1.107448713	0.64811243	1.631524479	4.05E-01
O94760	DDAH1	dimethylarginine dimethylaminohydrolase 1	1.968925182	0.606544	-1.38040174	6.36E-01	1.899530548	0.496346254	1.632162904	3.39E-01
P17174	GOT1	glutamic-oxaloacetic transaminase 1	1.54634301	0.606544	-1.287176172	6.57E-01	1.842711385	0.496346254	1.640826324	3.16E-01
P05981	HPN	hepsin	-0.252120206	0.9225917	-1.657524556	5.97E-01	-0.122482338	0.955733699	1.641254177	1.70E-01
P11279	LAMP1	lysosomal associated membrane protein 1	-0.099810115	0.9826758	1.235077706	7.45E-01	-0.798507267	0.727532832	1.645420586	3.88E-01
O43490	PROM1	prominin 1	-1.1068805	0.6613763	-0.800275929	5.97E-01	-0.754460096	0.52437214	1.652894664	2.17E-01
P01779	NA	NA	-0.372366756	0.9277913	-0.847435271	8.38E-01	-1.216640957	0.573392474	1.653228845	4.05E-01
Q6UXI9	NPNT	nephronectin	0.959467382	0.606544	-0.337884288	9.20E-01	1.160190058	0.49858242	1.657414368	1.70E-01

P11142	HSPA8	heat shock protein family A (Hsp70) member 8	-0.782585744	0.807657	-0.536391865	8.17E-01	-0.772870879	0.501690022	1.65783326	2.13E-01
P62879	GNB2	G protein subunit beta 2	-0.894778243	0.7117516	-0.566144356	7.73E-01	-0.848618367	0.49858242	1.657980746	1.70E-01
P00338	LDHA	lactate dehydrogenase A	-0.209542709	0.9502692	-0.188255427	9.56E-01	-0.198737913	0.941526432	1.664635152	3.15E-01
P05937	CALB1	calbindin 1	-0.308815017	0.9189448	-0.190465043	9.53E-01	0.065887522	0.98183702	1.667833997	1.70E-01
Q14254	FLOT2	flotillin 2	-1.568762189	0.606544	-1.015494763	5.97E-01	-1.461309209	0.49858242	1.668203194	3.10E-01
P15311	EZR	ezrin	-1.293194595	0.606544	-0.736793444	5.97E-01	-1.05562134	0.49858242	1.669576488	2.42E-01
P84095	RHOG	ras homolog family member G	-0.029445872	0.9928211	-0.303177711	9.43E-01	0.021384138	0.993572665	1.669889912	3.10E-01
Q8WWT9	SLC13A3	solute carrier family 13 member 3	-0.05386011	0.9860434	-0.47284303	9.00E-01	0.10714154	0.957265005	1.693824231	1.70E-01
Q99835	SMO	smoothened, frizzled class receptor	-0.015659581	0.9949261	-1.741910675	5.97E-01	0.110332147	0.955733699	1.694006009	1.70E-01
P05154	SERPINA5	serpin family A member 5	-0.197007249	0.9371571	-1.61793857	5.97E-01	-1.161531959	0.49858242	1.694221662	1.70E-01
O75936	BBOX1	gamma-butyrobetaine hydroxylase 1	-0.480268231	0.9124383	-0.259532423	9.55E-01	0.541069053	0.797911982	1.709010446	3.10E-01
Q53GD3	SLC44A4	solute carrier family 44 member 4	-0.080374955	0.9790738	-0.44523926	9.04E-01	-0.022678399	0.993572665	1.709341071	1.70E-01
Q99536	VAT1	vesicle amine transport 1	-1.108396982	0.6960871	0.041147957	9.95E-01	-1.481373779	0.49858242	1.723924032	3.35E-01
P09211	GSTP1	glutathione S-transferase pi 1	-0.237984628	0.9371571	-0.922626125	5.97E-01	-0.903303069	0.49858242	1.726199794	1.93E-01
P31949	S100A11	S100 calcium binding protein A11	-0.179340741	0.9478026	-0.453982147	8.19E-01	-1.146841129	0.49858242	1.729996284	1.70E-01
P01859	IGHG2	NA	0.521215062	0.9225917	-1.798515148	5.97E-01	-0.044481698	0.993572665	1.73135911	4.05E-01
P50148	GNAQ	G protein subunit alpha q	-1.377254945	0.606544	-0.887358942	5.97E-01	-0.793263099	0.49858242	1.739123562	1.54E-01
P08758	ANXA5	annexin A5	-0.054670126	0.9886331	-1.031658338	5.97E-01	-0.811130568	0.502615442	1.742140746	2.04E-01
O00560	SDCBP	syndecan binding protein	-0.76610323	0.8184847	-0.942747353	5.97E-01	-0.791295781	0.504696135	1.752397252	1.90E-01
O60635	TSPAN1	tetraspanin 1	-0.93826629	0.6648902	-0.135058445	9.55E-01	-0.978963301	0.49858242	1.754780581	1.17E-01
P02649	APOE	apolipoprotein E	0.693903176	0.8531508	-0.267772228	9.21E-01	0.484395245	0.843844089	1.755901663	3.10E-01
Q86XT2	VPS37D	VPS37D subunit of ESCRT-I	-1.318883093	0.6546304	-1.879012329	5.97E-01	-1.510778328	0.49858242	1.758910054	4.05E-01
P20339	RAB5A	RAB5A, member RAS oncogene family	0.908610725	0.7859971	-0.757050991	8.47E-01	0.193398799	0.948759698	1.761774941	3.47E-01
P20337	RAB3B	RAB3B, member RAS oncogene family	2.05925575	0.606544	-0.859632075	8.29E-01	1.23555345	0.49858242	1.765076357	3.10E-01
Q969P0	IGSF8	immunoglobulin superfamily member 8	-0.241671389	0.9294862	-0.265123514	9.37E-01	-0.015977341	0.993572665	1.765406402	1.70E-01
P62937	PPIA	peptidylprolyl isomerase A	-0.855822482	0.7649939	-0.885716382	5.97E-01	-0.911754209	0.49858242	1.768198235	1.70E-01
P01111	NRAS	NRAS proto-oncogene, GTPase	1.096003028	0.606544	-1.703458645	5.97E-01	0.190997379	0.91764061	1.772653377	1.70E-01
Q01518	CAP1	cyclase associated actin cytoskeleton regulatory protein 1	0.385875725	0.9189448	-0.281577217	9.44E-01	-0.764053778	0.717089821	1.773623553	2.88E-01
P00441	SOD1	superoxide dismutase 1	0.201220076	0.9433259	0.030529864	9.97E-01	-0.657729337	0.587650716	1.774400458	1.70E-01
P61020	RAB5B	RAB5B, member RAS oncogene family	-0.106380966	0.9815538	-1.390417745	5.97E-01	-0.358215936	0.88674295	1.78070845	3.11E-01
P15144	ANPEP	alanyl aminopeptidase, membrane	-1.116421302	0.72067	-0.776717944	6.67E-01	-1.060615499	0.49858242	1.786667584	2.61E-01
P18206	VCL	vinculin	-0.006631458	0.9953954	-1.38641081	5.97E-01	-1.136613313	0.561248865	1.788458451	2.87E-01
P07339	CTSD	cathepsin D	-1.554080089	0.606544	1.865864835	5.97E-01	-1.623041014	0.49858242	1.807386123	3.86E-01
Q9UM54	MYO6	myosin VI	0.738849862	0.7435884	-0.266883086	9.38E-01	1.266009761	0.49858242	1.808585373	1.70E-01
Q9Y2S2	CRYL1	crystallin lambda 1	0.898840318	0.6655294	-0.364135008	9.20E-01	1.268827476	0.49858242	1.81261068	1.70E-01
P62158	NA	NA	-0.36706218	0.9189448	0.485710089	9.18E-01	-1.549209272	0.49858242	1.814581607	1.70E-01
Q93088	BHMT	betaine--homocysteine S-methyltransferase	-0.30703405	0.9371571	-0.361386556	9.46E-01	-0.770686662	0.768785785	1.817593703	3.73E-01
O15143	ARPC1B	actin related protein 2/3 complex subunit 1B	1.135698071	0.606544	0.528022584	8.65E-01	-0.448378148	0.842116505	1.824146296	2.46E-01
Q9UN37	VPS4A	vacuolar protein sorting 4 homolog A	-1.475041249	0.606544	-1.324098794	5.97E-01	-1.048656531	0.49858242	1.824648948	1.83E-01
O43707	ACTN4	actinin alpha 4	-1.377682068	0.6546304	-0.010095582	9.97E-01	-2.574229779	0.496531829	1.824832186	3.90E-01
P62873	GNB1	G protein subunit beta 1	-0.84342011	0.7360888	-0.805159947	5.97E-01	-0.880664407	0.49858242	1.835256793	1.27E-01
Q9Y3E7	CHMP3	charged multivesicular body protein 3	0.806721316	0.7120423	-0.288166318	9.37E-01	1.285044144	0.49858242	1.835777349	1.70E-01
P16444	DPEP1	dipeptidase 1	-0.448265779	0.9141064	-0.438361536	8.38E-01	0.005469456	0.996266337	1.839506308	1.53E-01
P30085	CMPK1	cytidine/uridine monophosphate kinase 1	1.007071286	0.6253347	-0.191674163	9.55E-01	1.288758813	0.49858242	1.841084018	1.70E-01
P21281	ATP6V1B2	ATPase H+ transporting V1 subunit B2	1.914009591	0.606544	-1.682610929	6.02E-01	2.134470875	0.496346254	1.84239478	3.36E-01
P29972	AQP1	aquaporin 1 (Colton blood group)	-0.095915619	0.9826758	-0.340573112	9.48E-01	-1.68569562	0.49858242	1.844087643	3.10E-01
Q8WUM4	PDCD6IP	programmed cell death 6 interacting protein	-1.20383386	0.6401388	-0.841333975	5.97E-01	-1.075355106	0.49858242	1.847767038	1.83E-01
O95336	PGLS	6-phosphogluconolactonase	0.861875458	0.6929232	-0.31843042	9.27E-01	1.295555829	0.49858242	1.850794041	1.70E-01
P04264	KRT1	keratin 1	-2.808016024	0.606544	2.844126047	5.97E-01	-2.56620107	0.49858242	1.863289725	4.05E-01
Q9H299	SH3BGRL3	SH3 domain binding glutamate rich protein like 3	0.025638188	0.9928211	0.425524914	9.11E-01	-1.625196122	0.49858242	1.872025857	2.15E-01
Q8TCC7	SLC22A8	solute carrier family 22 member 8	1.144943183	0.606544	-0.703223559	8.42E-01	0.187944955	0.92818951	1.872488719	1.76E-01
P63104	YWHAZ	tyrosine 3-monooxygenase/tryptophan 5-monooxygenase a	-0.233193354	0.9392316	-1.268997112	5.97E-01	-1.113831513	0.49858242	1.872917089	1.99E-01
P0CG05	NA	NA	0.163857792	0.9674782	-0.888585311	5.97E-01	-0.877934918	0.549720269	1.875350536	2.89E-01
P48509	CD151	CD151 molecule (Raph blood group)	1.319842597	0.606544	-2.419311369	5.97E-01	-0.193825778	0.938574723	1.884126167	2.04E-01
O60494	CUBN	cubilin	-1.174239344	0.606544	-0.154955867	9.44E-01	-0.70461928	0.575609814	1.885482446	1.40E-01
P07195	LDHB	lactate dehydrogenase B	-0.338797368	0.9254988	-0.835886447	5.97E-01	-1.033348497	0.49858242	1.88672974	1.92E-01
P30626	SRI	sorcin	0.11676325	0.9699265	-1.223529463	5.97E-01	-0.141611191	0.957371069	1.886794851	1.82E-01
P98172	EFNB1	ephrin B1	-0.048034146	0.9886331	-0.541415201	8.95E-01	0.108575247	0.963063137	1.886865194	1.70E-01
P52565	ARHGDIA	Rho GDP dissociation inhibitor alpha	1.131624398	0.606544	-0.349266142	9.21E-01	1.323234661	0.49858242	1.89033523	1.70E-01
Q04760	GLO1	glyoxalase I	1.012521243	0.6325632	-0.345908508	9.21E-01	1.323953583	0.49858242	1.891362262	1.70E-01
P58876	HIST1H2BD	histone cluster 1 H2B family member d	0.413782373	0.9225917	1.204534515	5.97E-01	1.324987967	0.49858242	1.892839953	3.10E-01
P28676	GCA	grancalcin	1.714664411	0.606544	2.10133148	3.55E-01	1.206606214	0.49858242	1.896202776	3.10E-01
P20711	DDC	dopa decarboxylase	-0.150943201	0.9540881	-0.419669144	9.18E-01	0.118377436	0.957265005	1.901169655	1.70E-01
P00390	GSR	glutathione-disulfide reductase	1.238734088	0.606544	-0.91776723	8.03E-01	0.875771881	0.611776119	1.904261378	2.18E-01
P68371	TUBB4B	tubulin beta 4B class IVb	-0.203583953	0.9502692	0.831585112	8.37E-01	-0.936359053	0.49858242	1.908346973	2.09E-01
P62070	RRAS2	RAS related 2	-1.120304682	0.606544	0.409607176	9.17E-01	-1.620259224	0.49858242	1.912038649	2.09E-01
P17931	LGALS3	galectin 3	-0.027760141	0.9928211	-1.083202502	5.97E-01	-0.432765944	0.838926923	1.913265927	1.62E-01
P62820	RAB1A	RAB1A, member RAS oncogene family	0.790445082	0.853127	-0.075952327	9.85E-01	2.195662879	0.496346254	1.917352556	3.25E-01
P54652	HSPA2	heat shock protein family A (Hsp70) member 2	0.386686985	0.9248332	-1.085007482	8.19E-01	1.342961601	0.49858242	1.918516572	3.10E-01
P04217	A1BG	alpha-1-B glycoprotein	0.889202091	0.7117516	-1.311658096	6.87E-01	-0.681675412	0.778160011	1.918727737	2.18E-01
Q13228	SELENBP1	selenium binding protein 1	1.390049168	0.606544	-1.292902967	7.14E-01	1.048012901	0.549443178	1.918980565	2.93E-01
P27487	DPP4	dipeptidyl peptidase 4	-1.347957267	0.606544	-1.266038459	5.97E-01	-1.124303162	0.49858242	1.923158954	1.70E-01
Q9Y6W3	CAPN7	calpain 7	-1.668882789	0.606544	-1.327346938	5.97E-01	-1.54318687	0.49858242	1.924910165	1.87E-01
Q00610	CLTC	clathrin heavy chain	0.280803091	0.9371571	0.681986963	9.04E-01	0.695349064	0.793741327	1.928497697	3.10E-01
P62140	PPP1CB	protein phosphatase 1 catalytic subunit beta	0.959580423	0.6655294	-0.354028181	9.21E-01	1.355268035	0.49858242	1.936097192	1.70E-01
P53396	ACLY	ATP citrate lyase	1.21698166	0.606544	-0.25344312	9.44E-01	0.228478769	0.909075327	1.939038256	1.70E-01
P07355	ANXA2	annexin A2	-0.606234062	0.8925184	-1.406155763	5.97E-01	-1.591760261	0.49858242	1.947215353	2.57E-01
P54920	NAPA	NSF attachment protein alpha	0.026554511	0.9928211	0.567677444	8.42E-01	0.882276057	0.600120045	1.948812316	2.01E-01
Q9NZV1	CRIM1	cysteine rich transmembrane BMP regulator 1	0.011749382	0.9953954	-2.097936506	5.97E-01	0.086695478	0.97645047	1.959377827	1.70E-01
Q12805	EFEMP1	EGF containing fibulin extracellular matrix protein 1	-0.308631077	0.9225917	-1.674369126	5.97E-01	-1.439183542	0.49858242	1.974753534	1.70E-01
P63092	GNAS	GNAS complex locus	-1.74443985	0.606544	-0.872648303	5.97E-01	-1.216943874	0.49858242	1.978612925	1.70E-01
P07996	THBS1	thrombospondin 1	-1.539049491	0.606544	-0.429543193	9.18E-01	-2.770289085	0.496346254	1.978777918	1.70E-01
P13473	LAMP2	lysosomal associated membrane protein 2	-1.22793693	0.606544	1.40466611	5.97E-01	-0.914754822	0.674845824	1.981482215	2.46E-01
O94832	MYO1D	myosin ID	0.136532768	0.9688177	-1.556179017	5.97E-01	-0.646107016	0.783174956	1.983629367	2.69E-01
Q8N6Q3	CD177	CD177 molecule	2.608632547	0.606544	-0.822911384	8.42E-01	2.916125531	0.069521741	1.988770315	3.10E-01
P08571	CD14	CD14 molecule	1.292106723	0.606544	-0.550705015	9.00E-01	0.280903873	0.881117832	1.990306051	1.70E-01
P27348	YWHAQ	tyrosine 3-monooxygenase/tryptophan 5-monooxygenase a	1.03601554	0.641308	-0.38112407	9.21E-01	1.393415646	0.49858242	1.99059378	1.70E-01
Q9H2G2	SLK	STE20 like kinase	-1.686195319	0.606544	-0.520701182	9.14E-01	-2.169342285	0.496346254	1.994482406	2.96E-01
Q99816	TSG101	tumor susceptibility 101	-0.151779919	0.9706775	0.149089706	9.75E-01	-0.346535009	0.90682488	1.997585898	3.10E-01
A0PJK1	SLC5A10	solute carrier family 5 member 10	-0.154009572	0.9553457	-0.448166767	9.17E-01	0.028638088	0.993572665	2.002561047	1.70E-01
Q7LBR1	CHMP1B	charged multivesicular body protein 1B	-0.702169292	0.8583623	-1.529747165	5.97E-01	-1.433651137	0.49858242	2.005590792	2.36E-01
P41181	AQP2	aquaporin 2	-0.119917053	0.9688177	0.20498343	9.55E-01	-1.286082806	0.49858242	2.009974143	1.70E-01
P29992	GNA11	G protein subunit alpha 11	-1.402189603	0.606544	-1.474460162	6.02E-01	-2.721263905	0.181224175	2.012964367	3.10E-01
Q9BW04	C1orf116	chromosome 1 open reading frame 116	-1.300400676	0.6325632	-0.22959699	9.55E-01	-2.42435747	0.454961007	2.014568737	3.03E-01
O00592	PODXL	podocalyxin like	-1.443080122	0.606544	-1.681737697	5.97E-01	-1.217537979	0.49858242	2.028033575	1.70E-01
P38606	ATP6V1A	ATPase H+ transporting V1 subunit A	-1.268113638	0.6956246	-0.505184181	9.20E-01	-1.370306568	0.49858242	2.029182539	3.10E-01
Q9C0H2	TTYH3	tweety family member 3	-1.349696057	0.606544	1.571726813	5.97E-01	-0.794603946	0.768785785	2.029228179	3.10E-01
Q8N392	ARHGAP18	Rho GTPase activating protein 18	0.780464737	0.7649939	-0.160632198	9.56E-01	1.423127639	0.49858242	2.033039485	1.70E-01
P22314	UBA1	ubiquitin like modifier activating enzyme 1	1.022869281	0.7699942	-0.549014628	9.17E-01	0.131636022	0.971404327	2.034920314	3.10E-01
P16278	GLB1	galactosidase beta 1	-0.403703342	0.9157205	1.307267513	5.97E-01	0.010215148	0.99564488	2.05427752	1.70E-01
P15941	MUC1	mucin 1, cell surface associated	-1.153781075	0.606544	-0.427383712	8.26E-01	-0.991482206	0.49858242	2.059088067	8.02E-02
P01116	KRAS	KRAS proto-oncogene, GTPase	-1.270901767	0.606544	-0.617082785	8.65E-01	-2.259441649	0.298358728	2.061135538	2.13E-01
Q14894	CRYM	crystallin mu	1.285572798	0.606544	-0.406820702	9.20E-01	0.2325043	0.914165617	2.064132717	1.70E-01
P59665	DEFA1	defensin alpha 1	-2.242852282	0.606544	-1.932104171	5.97E-01	-2.206793543	0.49858242	2.066576848	3.10E-01
O00526	UPK2	uroplakin 2	-1.614559988	0.606544	0.222733064	9.55E-01	-1.9479568	0.49858242	2.06906632	1.87E-01
Q5IJ48	CRB2	crumbs cell polarity complex component 2	-0.083363721	0.9826758	-2.227103788	5.97E-01	-0.020994807	0.993572665	2.07029525	1.70E-01
P23526	AHCY	adenosylhomocysteinase	1.50129939	0.606544	-1.287051392	7.20E-01	1.141142022	0.49858242	2.077304726	2.32E-01
Q9NZZ3	CHMP5	charged multivesicular body protein 5	-0.765488699	0.8469743	-1.46148947	5.97E-01	-1.784456236	0.49858242	2.081826197	2.11E-01
P12821	ACE	angiotensin I converting enzyme	-1.331978153	0.606544	-2.350080722	2.40E-01	-0.831549543	0.697967647	2.086704683	2.28E-01
P04406	GAPDH	glyceraldehyde-3-phosphate dehydrogenase	0.370247662	0.9254988	-1.16530473	5.97E-01	-0.361264437	0.837774529	2.087866954	1.70E-01
P10909	CLU	clusterin	-0.284248714	0.9371571	-0.747561689	6.64E-01	-1.153295881	0.49858242	2.090882081	1.70E-01
P62745	RHOB	ras homolog family member B	-0.860553105	0.7435884	1.194763562	6.17E-01	-0.935960985	0.646294691	2.095241252	1.70E-01
P04004	VTN	vitronectin	0.912262698	0.7114717	-0.302991201	9.17E-01	0.23967529	0.941538111	2.097851175	1.99E-01
P60174	TPI1	triosephosphate isomerase 1	0.035509362	0.9928211	-1.301385709	5.97E-01	-0.751694747	0.588139908	2.102718079	1.70E-01
Q9HCY8	S100A14	S100 calcium binding protein A14	-0.411454765	0.9160511	-1.928139919	5.97E-01	-1.622035584	0.49858242	2.10297221	1.70E-01
Q8NI35	PATJ	PATJ crumbs cell polarity complex component	-0.99262409	0.6689541	-1.507603581	5.97E-01	-0.643906279	0.768785785	2.105379966	1.70E-01
P08195	SLC3A2	solute carrier family 3 member 2	1.442824047	0.6103356	-0.348460398	9.53E-01	1.498570763	0.49858242	2.110025218	3.10E-01
P40925	MDH1	malate dehydrogenase 1	0.266842905	0.9487033	-0.10794029	9.81E-01	0.103950974	0.98183702	2.116302405	3.10E-01
Q9HBG4	ATP6V0A4	ATPase H+ transporting V0 subunit a4	1.150805963	0.6253347	-0.36267781	9.27E-01	1.481464944	0.49858242	2.116378491	1.70E-01
P08582	MELTF	melanotransferrin	1.214116117	0.606544	-2.468762711	5.97E-01	0.970511472	0.567502564	2.123901623	1.82E-01
Q9UL25	RAB21	RAB21, member RAS oncogene family	1.022185062	0.6749466	-0.312240996	9.38E-01	1.487358284	0.49858242	2.124797548	1.70E-01
Q13621	SLC12A1	solute carrier family 12 member 1	-0.394610808	0.9189448	0.24975614	9.55E-01	-1.102843277	0.49858242	2.126999842	1.42E-01
Q96FN5	KIF12	kinesin family member 12	-0.86343365	0.807657	-0.221497346	9.56E-01	-1.525896472	0.49858242	2.13031234	1.70E-01
P49327	FASN	fatty acid synthase	1.456461796	0.606544	-0.323796312	9.37E-01	0.378831732	0.843844089	2.132405371	1.80E-01
P61586	RHOA	ras homolog family member A	-0.255608545	0.9371571	-1.023471204	5.97E-01	-1.207491903	0.49858242	2.134294632	1.42E-01
P47756	CAPZB	capping actin protein of muscle Z-line subunit beta	-0.719883429	0.8471769	-0.700395487	8.31E-01	-2.498149824	0.182545889	2.136048902	1.96E-01
P01833	PIGR	polymeric immunoglobulin receptor	-0.660543441	0.8763035	-0.886352317	6.53E-01	-2.024319628	0.49858242	2.137734596	2.08E-01
Q9Y287	ITM2B	integral membrane protein 2B	-1.129378017	0.6860777	-0.453087826	9.18E-01	-2.008715549	0.49858242	2.146504638	2.04E-01
Q9UBI6	GNG12	G protein subunit gamma 12	0.007736585	0.9953954	0.357566611	9.29E-01	-1.486902428	0.49858242	2.158439754	1.33E-01

P13645	KRT10	keratin 10	-3.331566981	0.606544	2.185961585	5.97E-01	-2.898132588	0.49858242	2.163070755	3.15E-01
Q96DG6	CMBL	carboxymethylenebutenolidase homolog	0.432379457	0.9189448	-0.448559698	9.20E-01	-0.482324947	0.83794346	2.170139684	1.95E-01
P49913	CAMP	cathelicidin antimicrobial peptide	0.540820163	0.9189448	0.89948013	8.13E-01	-1.498026915	0.49858242	2.171744745	3.03E-01
O15400	STX7	syntaxin 7	-0.783725809	0.807657	0.382646415	9.20E-01	-1.710879838	0.49858242	2.17182913	1.70E-01
P16870	CPE	carboxypeptidase E	1.238742307	0.606544	-0.252104716	9.53E-01	0.11533205	0.967124429	2.183052931	1.70E-01
B9A064	IGLL5	immunoglobulin lambda like polypeptide 5	1.473269922	0.7305966	1.446292279	7.70E-01	0.130893645	0.983062223	2.185457365	4.05E-01
O00161	SNAP23	synaptosome associated protein 23	-1.441777229	0.606544	-0.970522374	8.17E-01	-1.983617792	0.49858242	2.18639532	2.42E-01
P51159	RAB27A	RAB27A, member RAS oncogene family	1.269581325	0.606544	-0.410536457	9.21E-01	0.142894705	0.955733699	2.193309155	1.70E-01
P61981	YWHAG	tyrosine 3-monooxygenase/tryptophan 5-monooxygenase ac	-1.221270235	0.7117516	-2.396532235	5.97E-01	-1.055536343	0.64811243	2.193982773	2.91E-01
O15484	CAPN5	calpain 5	-1.245348215	0.6382731	0.154809217	9.74E-01	-1.819324278	0.49858242	2.197361677	1.70E-01
Q99497	PARK7	Parkinsonism associated deglycase	1.427726186	0.606544	-1.058737008	7.94E-01	0.948016026	0.630257102	2.199753946	1.99E-01
P15313	ATP6V1B1	ATPase H+ transporting V1 subunit B1	0.954266254	0.8184847	-0.985904007	8.33E-01	1.677205098	0.49858242	2.201014045	3.10E-01
O75348	ATP6V1G1	ATPase H+ transporting V1 subunit G1	1.017024027	0.6956246	-0.262842313	9.52E-01	1.545346018	0.49858242	2.207637168	1.70E-01
P42685	FRK	fyn related Src family tyrosine kinase	-0.186931259	0.9524426	-1.804080203	5.97E-01	0.375559473	0.850414292	2.209220216	1.74E-01
P00450	CP	ceruloplasmin	-0.374568214	0.9371571	0.40398594	9.51E-01	-1.150909843	0.655818184	2.21000253	3.27E-01
P54793	ARSF	arylsulfatase F	-0.481645607	0.8968647	-0.113937149	9.75E-01	-0.037037297	0.993572665	2.215864095	1.70E-01
P07737	PFN1	profilin 1	0.106850596	0.9769169	-0.963490469	5.97E-01	-0.764942887	0.549443178	2.215924141	8.71E-02
O43633	CHMP2A	charged multivesicular body protein 2A	-0.539218973	0.9018462	-1.281329143	5.97E-01	-1.446721136	0.49858242	2.218164279	1.63E-01
Q06830	PRDX1	peroxiredoxin 1	-0.782285767	0.8429929	-1.454582204	5.97E-01	-1.323866005	0.49858242	2.220268388	1.70E-01
P50895	BCAM	basal cell adhesion molecule (Lutheran blood group)	1.348398137	0.606544	-1.369329335	7.10E-01	1.085942773	0.532736131	2.220857977	2.00E-01
P33176	KIF5B	kinesin family member 5B	1.427749118	0.606544	-0.499264921	9.17E-01	1.556483609	0.49858242	2.223548013	1.70E-01
Q96A22	C11orf52	chromosome 11 open reading frame 52	-1.972960371	0.606544	0.430771346	9.21E-01	-2.548860065	0.105342907	2.229381455	1.70E-01
O60437	PPL	periplakin	-2.214809278	0.606544	-0.713615562	8.82E-01	-3.900626022	0.002880861	2.237266465	3.08E-01
P06733	ENO1	enolase 1	-0.128675747	0.9736479	-1.148102521	5.97E-01	-0.861043793	0.531943725	2.245424926	1.68E-01
P00558	PGK1	phosphoglycerate kinase 1	0.14064404	0.9679774	-0.867372213	6.57E-01	-0.758419501	0.569722295	2.248892855	1.10E-01
P01133	EGF	epidermal growth factor	-0.959446568	0.8086845	-1.528014871	6.64E-01	-2.246945618	0.49858242	2.251602509	2.44E-01
Q9NP85	NPHS2	NPHS2 stomatin family member, podocin	0.009837812	0.9953954	-1.578809468	5.97E-01	-1.417172209	0.49858242	2.25529545	2.69E-01
Q9UK41	VPS28	VPS28 subunit of ESCRT-I	-1.664710173	0.606544	-2.133172219	5.97E-01	-2.050308807	0.49858242	2.260323546	2.73E-01
Q6P1N0	CC2D1A	coiled-coil and C2 domain containing 1A	0.068909813	0.9860434	-1.167765771	7.42E-01	-0.010695959	0.99564488	2.265371331	1.70E-01
Q9UQB8	BAIAP2	BAI1 associated protein 2	-1.603389352	0.606544	-1.203082362	5.97E-01	-1.437980831	0.49858242	2.266924819	9.13E-02
P09467	FBP1	fructose-bisphosphatase 1	2.744008878	0.606544	-1.427018356	7.07E-01	2.454136891	0.241031753	2.270673292	2.16E-01
Q53TN4	CYBRD1	cytochrome b reductase 1	-1.164077012	0.632343	-0.022464387	9.97E-01	-0.862697604	0.706275784	2.271264361	1.76E-01
Q9BZQ8	NIBAN1	niban apoptosis regulator 1	1.395967182	0.606544	-0.341226202	9.37E-01	0.233563868	0.923885534	2.275144087	1.70E-01
P00747	PLG	plasminogen	-0.146766097	0.9674782	-0.670124187	8.03E-01	-0.690730492	0.796117674	2.276288558	1.50E-01
P07108	DBI	diazepam binding inhibitor, acyl-CoA binding protein	0.526057721	0.8607789	0.01386085	9.97E-01	0.510946438	0.768785785	2.279309017	7.88E-02
Q695T7	SLC6A19	solute carrier family 6 member 19	-0.986879381	0.7229718	1.232551032	6.80E-01	-2.102381219	0.49858242	2.287920734	1.75E-01
P52209	PGD	phosphogluconate dehydrogenase	0.474942763	0.8925184	0.180236078	9.55E-01	0.545716922	0.750413141	2.294907721	7.88E-02
P0CG06	NA	NA	0.14467975	0.9826758	-1.642754113	6.98E-01	-0.416054479	0.917193947	2.297136756	4.05E-01
O00194	RAB27B	RAB27B, member RAS oncogene family	-0.399424659	0.9266018	-0.234549139	9.55E-01	-0.439752577	0.860609107	2.305835574	2.15E-01
A5D8V6	VPS37C	VPS37C subunit of ESCRT-I	-1.719703449	0.606544	0.596195903	8.75E-01	-2.523388511	0.069521741	2.313630828	1.38E-01
P25311	AZGP1	alpha-2-glycoprotein 1, zinc-binding	1.470716029	0.606544	-0.45264389	9.20E-01	0.291638172	0.901781911	2.315598463	1.70E-01
Q9UGM3	DMBT1	deleted in malignant brain tumors 1	1.41687615	0.606544	0.534800693	9.04E-01	-0.441521851	0.865024195	2.325493286	1.96E-01
P07900	HSP90AA1	heat shock protein 90 alpha family class A member 1	0.411432527	0.9266018	-0.420768496	9.25E-01	0.28166152	0.938574723	2.345594375	2.39E-01
Q92485	SMPDL3B	sphingomyelin phosphodiesterase acid like 3B	-0.950491984	0.7460046	1.272080162	6.62E-01	-2.139520641	0.49858242	2.351086679	1.70E-01
Q8IYJ3	SYTL1	synaptotagmin like 1	0.522531316	0.9189448	0.256652502	9.55E-01	-0.205224923	0.945693298	2.355300005	1.95E-01
P80723	BASP1	brain abundant membrane attached signal protein 1	-0.062042185	0.9885399	0.331249243	9.44E-01	-1.978391753	0.496531829	2.361345722	1.32E-01
Q12913	PTPRJ	protein tyrosine phosphatase receptor type J	-0.639087588	0.8494246	0.137512513	9.75E-01	-1.573906893	0.49858242	2.370271994	8.02E-02
Q7L576	CYFIP1	cytoplasmic FMR1 interacting protein 1	-0.86951935	0.7699942	-0.195017593	9.56E-01	-0.806072365	0.723188519	2.370496093	1.70E-01
P21926	CD9	CD9 molecule	1.320217915	0.606544	-0.483789442	9.18E-01	0.836079845	0.744892198	2.377252602	1.96E-01
Q14974	KPNB1	karyopherin subunit beta 1	-0.384767729	0.9189448	-0.647717339	8.41E-01	-1.20900303	0.49858242	2.380819143	8.02E-02
Q15365	PCBP1	poly(rC) binding protein 1	-0.323799962	0.9336227	-0.739192728	8.27E-01	-0.773956392	0.692625656	2.388310875	1.53E-01
Q12907	LMAN2	lectin, mannose binding 2	0.055221161	0.9886331	-0.139573023	9.74E-01	-1.052187052	0.633269481	2.390801466	1.70E-01
Q9H0E2	TOLLIP	toll interacting protein	-0.848592264	0.7935006	1.267261432	6.52E-01	-0.703207941	0.768785785	2.39833207	1.70E-01
Q8IZP0	ABI1	abl interactor 1	-0.473723224	0.9086495	0.821893632	8.17E-01	-1.230424392	0.49858242	2.405156293	1.10E-01
P10301	RRAS	RAS related	0.591134735	0.8539819	-0.038994012	9.93E-01	0.544603996	0.768785785	2.411427838	7.88E-02
Q8NFU3	TSTD1	thiosulfate sulfurtransferase like domain containing 1	0.572686409	0.8583623	0.133459321	9.65E-01	0.430927676	0.835127935	2.430786681	7.88E-02
O95716	RAB3D	RAB3D, member RAS oncogene family	1.487960103	0.606544	-0.482391523	9.20E-01	1.703749513	0.49858242	2.433927876	1.70E-01
P60981	DSTN	destrin, actin depolymerizing factor	0.402888781	0.9266018	-0.101949685	9.81E-01	-0.859805912	0.745922129	2.458829653	2.04E-01
P30044	PRDX5	peroxiredoxin 5	0.76295213	0.8089368	0.898828922	7.83E-01	0.047178682	0.993572665	2.461856908	1.04E-01
P98164	LRP2	LDL receptor related protein 2	-1.100791563	0.660667	0.26967727	9.20E-01	-1.365141241	0.49858242	2.462158712	1.10E-01
P49773	HINT1	histidine triad nucleotide binding protein 1	0.59377755	0.9048944	-0.300353604	9.53E-01	-0.465945767	0.85038314	2.468616225	1.70E-01
P05090	APOD	apolipoprotein D	-0.673519157	0.8539819	-1.10342368	5.97E-01	-1.547379607	0.49858242	2.469769581	8.89E-02
P09543	CNP	2',3'-cyclic nucleotide 3' phosphodiesterase	-0.829290529	0.8359534	-0.676430388	8.61E-01	-1.857228681	0.49858242	2.480238667	1.70E-01
P23528	CFL1	cofilin 1	-0.956488413	0.7649939	-1.016039944	5.97E-01	-1.766338699	0.49858242	2.480241465	1.02E-01
P08133	ANXA6	annexin A6	0.644849653	0.8627587	-1.061854526	5.97E-01	0.607404984	0.796818344	2.489617032	1.19E-01
P62805	HIST1H4K	histone cluster 1 H4 family member k	0.886500991	0.8968647	1.676141096	7.62E-01	1.427055389	0.641378778	2.494926437	3.63E-01
P14174	MIF	macrophage migration inhibitory factor	-0.630892228	0.8923854	-0.014561205	9.97E-01	-1.21232085	0.49858242	2.496196513	9.31E-02
P13639	EEF2	eukaryotic translation elongation factor 2	1.58246374	0.606544	-0.048173566	9.93E-01	0.576608755	0.828228867	2.496677324	1.70E-01
Q6ZQN7	SLCO4C1	solute carrier organic anion transporter family member 4C1	-0.559035089	0.8601421	-1.549718896	5.97E-01	-0.471495628	0.828976468	2.501833361	8.02E-02
Q9UBR2	CTSZ	cathepsin Z	0.478863956	0.9048944	-1.778043152	5.97E-01	0.060437163	0.992516481	2.510485119	8.26E-02
Q92930	RAB8B	RAB8B, member RAS oncogene family	-1.049344789	0.7228295	-1.335873419	5.97E-01	-1.501854834	0.49858242	2.532039599	8.31E-02
O43895	XPNPEP2	X-prolyl aminopeptidase 2	0.407488643	0.9189448	-1.505116504	5.97E-01	0.073102895	0.986239379	2.537055329	8.40E-02
P62258	YWHAE	tyrosine 3-monooxygenase/tryptophan 5-monooxygenase ac	-0.458871097	0.9225917	-1.220232764	7.49E-01	-1.07157382	0.618149366	2.539522682	1.70E-01
P50502	ST13	ST13 Hsp70 interacting protein	0.518254705	0.8944554	-0.012786704	9.97E-01	0.568540265	0.768785785	2.540781217	7.88E-02
Q9BY43	CHMP4A	charged multivesicular body protein 4A	-1.381974379	0.606544	0.311910539	9.37E-01	-1.560770252	0.49858242	2.542559515	7.48E-02
P61026	RAB10	RAB10, member RAS oncogene family	-0.970684266	0.7649939	0.289204181	9.53E-01	-1.396775665	0.49858242	2.561163011	8.31E-02
P13861	PRKAR2A	protein kinase cAMP-dependent type II regulatory subunit al	-0.857631865	0.8421065	1.518418288	6.36E-01	-1.203449672	0.52135768	2.564618783	1.70E-01
Q04609	FOLH1	folate hydrolase 1	1.369964101	0.6325632	-0.333694721	9.44E-01	0.043241744	0.993572665	2.564856222	1.70E-01
Q96J02	ITCH	itchy E3 ubiquitin protein ligase	0.414670668	0.9189448	0.21333907	9.55E-01	0.55575846	0.78099142	2.578323357	7.88E-02
Q9UQN3	CHMP2B	charged multivesicular body protein 2B	-0.999228935	0.7649939	-1.653617182	5.97E-01	-1.600722331	0.49858242	2.579101355	9.31E-02
P34896	SHMT1	serine hydroxymethyltransferase 1	-0.15254774	0.9651208	0.472108138	9.18E-01	-0.745669025	0.708677957	2.589546656	8.02E-02
Q9H4M9	EHD1	EH domain containing 1	-0.159655446	0.9603317	-1.161970089	5.97E-01	-0.872473466	0.49858242	2.609803687	4.42E-02
P06744	GPI	glucose-6-phosphate isomerase	0.797917343	0.807657	0.675065788	8.47E-01	0.229616616	0.932111286	2.610348556	9.01E-02
Q96EY5	MVB12A	multivesicular body subunit 12A	-1.051816623	0.7795884	-0.92015661	8.26E-01	-2.092604541	0.49858242	2.610472249	1.70E-01
O75347	TBCA	tubulin folding cofactor A	0.64503123	0.8539819	0.09987358	9.76E-01	-0.765426043	0.725716007	2.61082102	7.88E-02
Q9Y5K6	CD2AP	CD2 associated protein	-0.44795449	0.9189448	-0.241332535	9.55E-01	-1.564033921	0.49858242	2.640938696	8.02E-02
O75083	WDR1	WD repeat domain 1	-0.816816407	0.8089368	-0.81768219	6.21E-01	-1.189402712	0.49858242	2.64444527	5.83E-02
P30041	PRDX6	peroxiredoxin 6	2.107894389	0.606544	-0.597194931	9.20E-01	0.816840857	0.78099142	2.649993569	2.18E-01
Q7L7X3	TAOK1	TAO kinase 1	-0.76960354	0.7699942	0.100968109	9.76E-01	-0.678191371	0.767371774	2.651142454	7.96E-02
P01042	KNG1	kininogen 1	-0.069571996	0.9885399	-0.245494472	9.56E-01	-2.062020752	0.49858242	2.652123209	1.39E-01
Q5VW32	BROX	BRO1 domain and CAAX motif containing	-0.912586987	0.8245096	-1.825804032	5.97E-01	-1.563539405	0.49858242	2.660711333	1.14E-01
Q9Y6R7	FCGBP	Fc fragment of IgG binding protein	0.375018854	0.9341569	-0.094170816	9.81E-01	-1.016278311	0.734141735	2.665581241	2.08E-01
O00322	UPK1A	uroplakin 1A	-2.301830956	0.606544	0.413883238	9.37E-01	-3.326618826	0.041725689	2.667894698	1.70E-01
P47755	CAPZA2	capping actin protein of muscle Z-line subunit alpha 2	-0.272747269	0.9371571	0.689793064	8.42E-01	-0.8910989	0.657136965	2.682939371	8.02E-02
Q16827	PTPRO	protein tyrosine phosphatase receptor type O	0.901580198	0.7699942	-2.290431842	5.97E-01	0.217571978	0.93909278	2.689044048	8.99E-02
P07947	YES1	YES proto-oncogene 1, Src family tyrosine kinase	0.3749082	0.9311092	-0.223343853	9.56E-01	0.681440962	0.796476479	2.691741424	1.70E-01
Q07075	ENPEP	glutamyl aminopeptidase	-0.686426528	0.8583623	0.190356088	9.57E-01	-1.426787869	0.49858242	2.691950136	8.89E-02
Q9UI12	ATP6V1H	ATPase H+ transporting V1 subunit H	0.660535915	0.8556187	-0.215996175	9.55E-01	0.739681329	0.690002674	2.695198381	8.02E-02
P31639	SLC5A2	solute carrier family 5 member 2	-0.828157446	0.7435884	0.154405016	9.62E-01	-1.061968041	0.627475017	2.709048668	7.88E-02
P22748	CA4	carbonic anhydrase 4	-0.309712834	0.9312478	-1.117723989	6.74E-01	-1.62661376	0.49858242	2.710898266	7.48E-02
P08107	NA	NA	0.570491039	0.9124383	-1.634998251	5.97E-01	0.213258029	0.948759698	2.717301323	1.32E-01
Q15599	SLC9A3R2	SLC9A3 regulator 2	0.958632901	0.7493815	-0.823132703	8.31E-01	0.162253419	0.955733699	2.731241673	8.40E-02
P00966	ASS1	argininosuccinate synthase 1	-0.318581822	0.9341569	-0.238230188	9.55E-01	-1.675545194	0.49858242	2.73332392	7.88E-02
P62834	RAP1A	RAP1A, member of RAS oncogene family	-1.342719821	0.6382731	0.797586311	8.37E-01	-0.384040226	0.902183441	2.749329212	1.70E-01
P12931	SRC	SRC proto-oncogene, non-receptor tyrosine kinase	-0.76941539	0.8583623	-1.217963677	7.53E-01	-2.906071264	0.126725502	2.751581111	1.56E-01
Q99102	MUC4	mucin 4, cell surface associated	3.045594497	0.606544	-1.450669206	7.42E-01	1.086466884	0.644588499	2.754170418	1.70E-01
Q9NQ84	GPRC5C	G protein-coupled receptor class C group 5 member C	-1.56045667	0.6106023	-0.163926035	9.75E-01	-2.08251757	0.49858242	2.75946163	1.56E-01
P68104	EEF1A1	eukaryotic translation elongation factor 1 alpha 1	-0.096876771	0.9828123	-0.233148494	9.55E-01	-1.568227677	0.49858242	2.759573766	8.40E-02
P68363	TUBA1B	tubulin alpha 1b	0.221510738	0.9614725	0.583980952	9.18E-01	-0.341247386	0.923322861	2.760079627	2.22E-01
P12429	ANXA3	annexin A3	1.326772446	0.6857792	0.544498548	9.20E-01	0.667469371	0.807619793	2.766092465	1.70E-01
O75340	PDCD6	programmed cell death 6	-0.05579753	0.9893918	-0.299767705	9.44E-01	-0.7177307	0.768963521	2.771285931	9.01E-02
P31946	YWHAB	tyrosine 3-monooxygenase/tryptophan 5-monooxygenase ac	-0.463398523	0.9225917	-0.108798593	9.81E-01	-1.87820174	0.49858242	2.776651216	1.05E-01
Q96KP4	CNDP2	carnosine dipeptidase 2	-0.128017286	0.9808828	-1.067528361	8.13E-01	-0.995081475	0.651775557	2.778924269	1.53E-01
Q8IY95	TMEM192	transmembrane protein 192	0.67938454	0.8539819	-0.497753594	9.12E-01	1.451924991	0.49858242	2.783436132	8.02E-02
Q9UNF0	PACSIN2	protein kinase C and casein kinase substrate in neurons 2	-0.302485344	0.9326901	-1.065889808	6.94E-01	-1.511842657	0.49858242	2.783856119	5.55E-02
P63000	RAC1	Rac family small GTPase 1	-1.01487229	0.7727259	-1.543376752	5.97E-01	-1.632996484	0.49858242	2.787477877	8.02E-02
Q03154	ACY1	aminoacylase 1	1.006646127	0.7649939	-1.111309594	8.17E-01	0.474806115	0.843844089	2.800583318	1.14E-01
P08754	GNAI3	G protein subunit alpha i3	-1.114918838	0.7632825	-0.304610201	9.55E-01	-1.092562903	0.695724656	2.804273308	1.70E-01
P01877	IGHA2	NA	1.639347064	0.7117516	-1.50213999	7.82E-01	-2.852894604	0.49858242	2.805096415	3.18E-01
O75874	IDH1	isocitrate dehydrogenase (NADP(+)) 1, cytosolic	-0.305916017	0.9371571	-0.24907852	9.55E-01	-1.808375246	0.49858242	2.808989888	1.04E-01
O00187	MASP2	mannan binding lectin serine peptidase 2	0.471366154	0.9189448	0.149496967	9.75E-01	-0.454309492	0.878195	2.809734605	1.25E-01
Q9H223	EHD4	EH domain containing 4	-1.207547157	0.6703732	-1.419725008	5.97E-01	-1.658908704	0.49858242	2.812981616	7.71E-02
Q10588	BST1	bone marrow stromal cell antigen 1	1.223916338	0.606544	-0.978454244	7.64E-01	-0.253967256	0.909075327	2.813923039	7.56E-03
O00468	AGRN	agrin	0.087926382	0.9826758	-1.800230128	5.97E-01	-1.482712942	0.510244004	2.81727399	8.02E-02
P01591	JCHAIN	joining chain of multimeric IgA and IgM	-0.430350451	0.9225917	0.077101962	9.82E-01	-0.63906765	0.803999483	2.833084131	8.02E-02
P08183	ABCB1	ATP binding cassette subfamily B member 1	-1.053818113	0.7649939	0.132237673	9.76E-01	-1.367113989	0.49858242	2.841415151	8.71E-02
Q9H9H4	VPS37B	VPS37B subunit of ESCRT-I	-0.803274386	0.8394879	-1.061331652	7.85E-01	-1.364608049	0.49858242	2.84718166	9.31E-02
P05109	S100A8	S100 calcium binding protein A8	1.826686256	0.606544	-1.771088871	5.97E-01	0.236396924	0.949908781	2.847194066	1.70E-01
P54289	CACNA2D1	calcium voltage-gated channel auxiliary subunit alpha2delta	-0.462912223	0.9189448	-0.874314587	8.31E-01	-1.12497977	0.589644232	2.852961335	8.71E-02

P35579	MYH9	myosin heavy chain 9	0.689544057	0.8539819	-0.693572483	7.38E-01	-0.065943178	0.990898281	2.853361886	4.77E-02
Q96FZ7	CHMP6	charged multivesicular body protein 6	-0.305567126	0.9371571	-1.711968201	5.97E-01	-1.931474785	0.49858242	2.864090742	8.45E-02
P68431	HIST1H3H	histone cluster 1 H3 family member h	1.502340944	0.6901105	0.279031036	9.56E-01	1.716051968	0.49858242	2.871217418	2.26E-01
P07437	TUBB	tubulin beta class I	1.817493521	0.606544	1.378524058	7.85E-01	0.534553456	0.867184582	2.876761743	1.96E-01
Q7Z4W1	DCXR	dicarbonyl and L-xylulose reductase	0.938620442	0.7795884	-0.011517842	9.97E-01	0.760580807	0.707797878	2.885924474	8.02E-02
P10768	ESD	esterase D	0.669939653	0.8607789	-0.016845374	9.97E-01	0.692755051	0.745922129	2.891469898	7.88E-02
P04080	CSTB	cystatin B	0.155060241	0.9576591	-0.942036036	7.84E-01	-0.423986315	0.843844089	2.904540826	7.56E-03
Q8N5I2	ARRDC1	arrestin domain containing 1	-0.364174472	0.9225917	-0.831895467	8.08E-01	-0.461191056	0.847054128	2.920264824	4.78E-02
Q15833	STXBP2	syntaxin binding protein 2	-0.754320017	0.8394879	0.16752167	9.62E-01	-1.947749824	0.49858242	2.937643416	4.03E-02
P35241	RDX	radixin	-0.04089785	0.9928211	0.363064519	9.51E-01	-2.171597683	0.49858242	2.942875152	1.14E-01
O43175	PHGDH	phosphoglycerate dehydrogenase	0.897741238	0.807657	0.085307616	9.81E-01	0.608777562	0.796117674	2.949425006	8.02E-02
O14773	TPP1	tripeptidyl peptidase 1	-0.46888572	0.9189448	1.534080104	6.36E-01	-1.764950341	0.49858242	2.955336824	3.64E-02
P30086	PEBP1	phosphatidylethanolamine binding protein 1	-0.302144134	0.9371571	-0.465203529	9.24E-01	-1.755550607	0.49858242	2.974511017	8.02E-02
P35080	PFN2	profilin 2	0.763777077	0.8429929	-1.942338771	5.97E-01	0.060094286	0.992706054	2.981528553	7.88E-02
P18669	PGAM1	phosphoglycerate mutase 1	0.953019533	0.7795884	0.841670524	8.26E-01	0.373459682	0.881117832	3.011967292	7.96E-02
Q6UX06	OLFM4	olfactomedin 4	-0.762911915	0.8583623	0.928115409	8.19E-01	-1.911229308	0.49858242	3.043128513	6.74E-02
P61006	RAB8A	RAB8A, member RAS oncogene family	0.831376244	0.852021	0.587406123	9.17E-01	0.256805846	0.940995344	3.050542447	1.04E-01
Q15286	RAB35	RAB35, member RAS oncogene family	-0.591429813	0.9042183	-0.360166615	9.39E-01	-2.026306504	0.49858242	3.052396307	4.77E-02
P06396	GSN	gelsolin	0.845987862	0.8089368	-1.78234632	5.97E-01	-0.770036789	0.768785785	3.059297045	6.89E-02
Q9UKS6	PACSIN3	protein kinase C and casein kinase substrate in neurons 3	0.688686584	0.8583623	-2.225619104	5.97E-01	0.055866015	0.993572665	3.069927262	6.95E-02
P21796	VDAC1	voltage dependent anion channel 1	0.604439088	0.8968647	-0.574091734	9.04E-01	1.390936795	0.49858242	3.074898832	7.88E-02
Q92597	NDRG1	N-myc downstream regulated 1	-0.556115942	0.9189448	1.275948108	7.92E-01	-0.584076264	0.838926923	3.081093985	1.53E-01
Q8N271	PROM2	prominin 2	-0.50309935	0.9189448	-0.588484575	9.04E-01	-0.645850929	0.796476479	3.084670129	4.34E-02
P50395	GDI2	GDP dissociation inhibitor 2	0.425015433	0.9225917	-0.903966701	8.26E-01	-1.972529191	0.49858242	3.084921633	6.74E-02
P61204	ARF3	ADP ribosylation factor 3	0.328858209	0.9225917	-1.042202162	7.07E-01	-1.14798901	0.49858242	3.110965137	7.36E-04
P06702	S100A9	S100 calcium binding protein A9	0.740081118	0.8968647	-1.306455295	7.85E-01	-0.286446646	0.942820718	3.137495401	1.67E-01
P04216	THY1	Thy-1 cell surface antigen	-0.465630777	0.9189448	-2.05138747	5.97E-01	-0.749214389	0.744892198	3.154643777	9.19E-03
Q9UBV8	PEF1	penta-EF-hand domain containing 1	-0.863951993	0.7649939	-0.105195587	9.76E-01	-1.820236035	0.49858242	3.155459618	8.64E-03
P02788	LTF	lactotransferrin	0.317186682	0.9540881	0.138888729	9.81E-01	-1.904786757	0.49858242	3.162710379	2.18E-01
Q8NFJ5	GPRC5A	G protein-coupled receptor class C group 5 member A	-0.231730205	0.9371571	0.583725404	8.54E-01	-0.457900012	0.846210281	3.182127031	7.61E-03
Q12929	EPS8	epidermal growth factor receptor pathway substrate 8	-1.57655911	0.606544	-0.223331202	9.61E-01	-1.866485719	0.49858242	3.205689918	7.40E-02
P11234	RALB	RAS like proto-oncogene B	-0.452085242	0.9189448	-1.687311474	5.97E-01	-0.512673807	0.837774529	3.213022115	1.11E-02
P14384	CPM	carboxypeptidase M	-0.464328081	0.8925184	-0.548451719	8.65E-01	-1.107611724	0.49858242	3.213859622	1.18E-06
Q96RF0	SNX18	sorting nexin 18	-0.021909072	0.9949121	0.496370127	9.04E-01	-0.053157803	0.992706054	3.241519186	9.19E-03
Q00796	SORD	sorbitol dehydrogenase	0.627723477	0.9189448	0.356408969	9.55E-01	0.042032869	0.993572665	3.247958343	1.67E-01
Q9BRK3	MXRA8	matrix remodeling associated 8	-0.119773257	0.9734313	0.594346019	8.54E-01	-0.489702647	0.842123846	3.2507557	7.56E-03
Q13277	STX3	syntaxin 3	-1.053128915	0.6699589	-0.080399007	9.81E-01	-0.85418939	0.701843676	3.276818395	6.31E-03
P42330	AKR1C3	aldo-keto reductase family 1 member C3	-0.92596089	0.7377702	-0.577192129	9.04E-01	-2.004080046	0.49858242	3.287463908	6.31E-03
Q9Y6E0	STK24	serine/threonine kinase 24	0.832308019	0.7649939	0.005906476	9.97E-01	0.29547619	0.911006949	3.296502539	6.31E-03
Q8IWA5	SLC44A2	solute carrier family 44 member 2	-0.214404262	0.9476282	-1.293641391	7.14E-01	-0.674448574	0.795086021	3.306258762	7.61E-03
P08174	CD55	CD55 molecule (Cromer blood group)	-1.04060608	0.6821004	-0.511071979	9.17E-01	-2.268056684	0.49858242	3.311183997	6.31E-03
P07948	LYN	LYN proto-oncogene, Src family tyrosine kinase	0.906890235	0.7849608	-0.044063438	9.95E-01	-0.556124349	0.838926923	3.312438806	3.46E-02
P51153	RAB13	RAB13, member RAS oncogene family	-1.407468531	0.6382731	0.189303131	9.67E-01	-1.932768995	0.49858242	3.397455794	5.70E-02
P07288	KLK3	kallikrein related peptidase 3	1.10593054	0.7795884	0.042110872	9.95E-01	0.64297616	0.817499764	3.39845216	8.02E-02
Q92817	EVPL	envoplakin	-1.228599121	0.6981395	-0.648847404	9.04E-01	-4.178199462	0.001691701	3.418807832	7.88E-02
Q9HD42	CHMP1A	charged multivesicular body protein 1A	-0.103529643	0.9828123	-1.070625808	8.17E-01	-1.024594214	0.666116504	3.424915151	3.71E-02
Q8TE68	EPS8L1	EPS8 like 1	-0.272482855	0.9371571	-0.973850956	8.17E-01	-1.163879065	0.549233884	3.453753773	4.13E-03
Q9BUL8	PDCD10	programmed cell death 10	-1.052226368	0.7117516	-0.227389557	9.55E-01	-2.076675728	0.49858242	3.510793706	6.80E-03
O75131	CPNE3	copine 3	-1.32086343	0.6719857	-0.48238831	9.20E-01	-1.077374818	0.648268555	3.519118176	1.75E-02
O96009	NAPSA	napsin A aspartic peptidase	0.643651951	0.8757534	0.103965301	9.81E-01	-0.600210971	0.817499764	3.519161847	6.74E-03
Q9H2K8	TAOK3	TAO kinase 3	0.275345471	0.9371571	-1.811963373	5.97E-01	-0.513241844	0.838926923	3.537583678	7.61E-03
Q9NRX4	PHPT1	phosphohistidine phosphatase 1	1.390967461	0.606544	0.530792304	9.04E-01	1.098880704	0.567472638	3.555183927	7.56E-03
Q9H4G4	GLIPR2	GLI pathogenesis related 2	1.522119833	0.606544	1.905638435	5.97E-01	1.124325625	0.666116504	3.568686053	4.78E-02
P04083	ANXA1	annexin A1	0.869999314	0.8340657	-2.095065724	5.97E-01	-1.089281295	0.696332125	3.737734985	1.70E-02
Q9H6S3	EPS8L2	EPS8 like 2	-1.166590833	0.72067	-0.279518045	9.55E-01	-2.3882929	0.49858242	3.771438019	7.61E-03
P12277	CKB	creatine kinase B	1.049858879	0.8340657	0.073329055	9.89E-01	0.883049091	0.767371774	3.840854082	7.88E-02
Q9UHR4	BAIAP2L1	BAI1 associated protein 2 like 1	-1.820418962	0.606544	0.02568449	9.97E-01	-3.406373438	0.069521741	3.853405556	1.88E-02
O15162	PLSCR1	phospholipid scramblase 1	-0.028050281	0.9928211	0.079963966	9.81E-01	-0.296715015	0.904452946	3.872777375	1.21E-13
P16152	CBR1	carbonyl reductase 1	-0.763794681	0.8812913	-0.007961018	9.97E-01	-1.975552959	0.49858242	4.03883054	7.61E-03
P15309	ACPP	acid phosphatase, prostate	1.120457449	0.8394879	0.122954538	9.81E-01	0.737569423	0.835127935	4.168572518	7.88E-02
Q16651	PRSS8	serine protease 8	-0.503752081	0.9189448	-0.465421969	9.20E-01	-1.424088089	0.503103844	4.271527203	1.18E-06
P15328	FOLR1	folate receptor alpha	0.573798782	0.9123637	-1.237591401	8.10E-01	-0.136290239	0.972684176	4.373879406	5.61E-06
P17927	CR1	complement C3b/C4b receptor 1 (Knops blood group)	1.675854733	0.606544	-1.952145785	5.97E-01	0.589163315	0.846688909	4.654277339	5.03E-04
P68032	ACTC1	actin alpha cardiac muscle 1	1.214381541	0.8556187	1.692875894	8.03E-01	-0.157589002	0.983062223	4.823491673	1.02E-01
O15393	TMPRSS2	transmembrane serine protease 2	0.216270458	0.9565129	-0.247321157	9.55E-01	-1.090664839	0.687784296	5.332022976	0.00E+00

Table S3. Proteins contained in each cluster

Cluster 1

Uniprot	HGNC	Wikigene
P08246	ELANE	elastase, neutrophil expressed
Q9NRD8	DUOX2	dual oxidase 2
P12814	ACTN1	actinin alpha 1
P14780	MMP9	matrix metallopeptidase 9
P16278	GLB1	galactosidase beta 1
P84095	RHOG	ras homolog family member G
P00450	CP	ceruloplasmin
P01591	JCHAIN	joining chain of multimeric IgA and IgM
P02788	LTF	lactotransferrin
P05164	MPO	myeloperoxidase
P62805	HIST4H4	histone cluster 4 H4
P31639	SLC5A2	solute carrier family 5 member 2
P68431	HIST1H3A	histone cluster 1 H3 family member a
Q96KK5	HIST1H2AH	histone cluster 1 H2A family member h
P34896	SHMT1	serine hydroxymethyltransferase 1
Q9Y6R7	FCGBP	Fc fragment of IgG binding protein
Q8NFJ5	GPRC5A	G protein-coupled receptor class C group 5 member A
Q96RF0	SNX18	sorting nexin 18
P02792	FTL	ferritin light chain
P02794	FTH1	ferritin heavy chain 1
A5D8V6	VPS37C	VPS37C, ESCRT-I subunit
O00159	MYO1C	myosin IC
O00194	RAB27B	RAB27B, member RAS oncogene family
O00322	UPK1A	uroplakin 1A
O15400	STX7	syntaxin 7
O15484	CAPN5	calpain 5
O43707	ACTN4	actinin alpha 4
O75874	IDH1	isocitrate dehydrogenase (NADP(+)) 1, cytosolic
P03973	SLPI	secretory leukocyte peptidase inhibitor
P01116	KRAS	KRAS proto-oncogene, GTPase
P08174	CD55	CD55 molecule (Cromer blood group)
P08754	GNAI3	G protein subunit alpha i3
P09543	CNP	2',3'-cyclic nucleotide 3' phosphodiesterase
P12931	SRC	SRC proto-oncogene, non-receptor tyrosine kinase
P13473	LAMP2	lysosomal associated membrane protein 2
P13861	PRKAR2A	protein kinase cAMP-dependent type II regulatory subunit alpha
P14174	MIF	macrophage migration inhibitory factor
P14384	CPM	carboxypeptidase M
P14550	AKR1A1	aldo-keto reductase family 1 member A1
P16152	CBR1	carbonyl reductase 1
P23528	CFL1	cofilin 1
P30086	PEBP1	phosphatidylethanolamine binding protein 1
P31946	YWHAB	tyrosine 3-monooxygenase/tryptophan 5-monooxygenase activation protein beta
P31947	SFN	stratifin
P38606	ATP6V1A	ATPase H+ transporting V1 subunit A
P40121	CAPG	capping actin protein, gelsolin like
P41181	AQP2	aquaporin 2
P47755	CAPZA2	capping actin protein of muscle Z-line alpha subunit 2
P47756	CAPZB	capping actin protein of muscle Z-line beta subunit
P51153	RAB13	RAB13, member RAS oncogene family
P59665	DEFA1	defensin alpha 1
P62258	YWHAE	tyrosine 3-monooxygenase/tryptophan 5-monooxygenase activation protein epsilon
P62745	RHOB	ras homolog family member B
P68104	EEF1A1	eukaryotic translation elongation factor 1 alpha 1
P80723	BASP1	brain abundant membrane attached signal protein 1
Q07075	ENPEP	glutamyl aminopeptidase
P61769	B2M	beta-2-microglobulin
Q12929	EPS8	epidermal growth factor receptor pathway substrate 8
Q13277	STX3	syntaxin 3
Q14974	KPNB1	karyopherin subunit beta 1

Uniprot	HGNC	Wikigene
Q15286	RAB35	RAB35, member RAS oncogene family
O15143	ARPC1B	actin related protein 2/3 complex subunit 1B
P08571	CD14	CD14 molecule
Q15365	PCBP1	poly(rC) binding protein 1
P18669	PGAM1	phosphoglycerate mutase 1
Q15833	STXBP2	syntaxin binding protein 2
P24158	PRTN3	proteinase 3
Q16651	PRSS8	protease, serine 8
P50552	VASP	vasodilator stimulated phosphoprotein
Q7L576	CYFIP1	cytoplasmic FMR1 interacting protein 1
P68032	ACTC1	actin, alpha, cardiac muscle 1
Q8IZP0	ABI1	abl interactor 1
Q92485	SMPDL3B	sphingomyelin phosphodiesterase acid like 3B
Q13510	ASAH1	N-acylsphingosine amidohydrolase 1
Q92597	NDRG1	N-myc downstream regulated 1
Q13113	PDZK1IP1	PDZK1 interacting protein 1
Q9H299	SH3BGRL3	SH3 domain binding glutamate rich protein like 3
Q92817	EVPL	envoplakin
O15162	PLSCR1	phospholipid scramblase 1
Q96A22	C11orf52	chromosome 11 open reading frame 52
O15393	TMPRSS2	transmembrane protease, serine 2
Q96EY5	MVB12A	multivesicular body subunit 12A
Q96FN5	KIF12	kinesin family member 12
P04080	CSTB	cystatin B
Q96KP4	CNDP2	carnosine dipeptidase 2
P11234	RALB	RAS like proto-oncogene B
Q9BUL8	PDCD10	programmed cell death 10
Q9BW04	C1orf116	chromosome 1 open reading frame 116
P17927	CR1	complement C3b/C4b receptor 1 (Knops blood group)
Q9BY43	CHMP4A	charged multivesicular body protein 4A
Q9C0H2	TTYH3	tweety family member 3
Q9H0E2	TOLLIP	toll interacting protein
Q9H223	EHD4	EH domain containing 4
Q9H2G2	SLK	STE20 like kinase
Q8N5I2	ARRDC1	arrestin domain containing 1
Q9H6S3	EPS8L2	EPS8 like 2
Q9H9H4	VPS37B	VPS37B, ESCRT-I subunit
Q9NZH0	GPRC5B	G protein-coupled receptor class C group 5 member B
Q9H4G4	GLIPR2	GLI pathogenesis related 2
Q9NZM1	MYOF	myoferlin
P62834	RAP1A	RAP1A, member of RAS oncogene family
Q9NZZ3	CHMP5	charged multivesicular body protein 5
Q9UBI6	GNG12	G protein subunit gamma 12
Q53TN4	CYBRD1	cytochrome b reductase 1
Q9UBV8	PEF1	penta-EF-hand domain containing 1
Q9UHR4	BAIAP2L1	BAI1 associated protein 2 like 1
Q9UK41	VPS28	VPS28, ESCRT-I subunit
Q9Y5K6	CD2AP	CD2 associated protein
P05109	S100A8	S100 calcium binding protein A8
P22732	SLC2A5	solute carrier family 2 member 5
P02679	FGG	fibrinogen gamma chain
P04003	C4BPA	complement component 4 binding protein alpha
P00751	CFB	complement factor B
P61626	LYZ	lysozyme
Q93077	HIST1H2AC	histone cluster 1 H2A family member c
P02763	ORM1	orosomucoid 1
P21333	FLNA	filamin A
P43490	NAMPT	nicotinamide phosphoribosyltransferase
P98160	HSPG2	heparan sulfate proteoglycan 2
Q14314	FGL2	fibrinogen like 2
P00747	PLG	plasminogen
P06702	S100A9	S100 calcium binding protein A9
P20073	ANXA7	annexin A7

Uniprot	HGNC	Wikigene
P0C0L4	C4A	complement C4A (Rodgers blood group)
P35080	PFN2	profilin 2
Q6UX06	OLFM4	olfactomedin 4
P61204	ARF3	ADP ribosylation factor 3
Q9H2K8	TAOK3	TAO kinase 3
Q9Y6E0	STK24	serine/threonine kinase 24
P11279	LAMP1	lysosomal associated membrane protein 1
P29972	AQP1	aquaporin 1 (Colton blood group)
P50395	GDI2	GDP dissociation inhibitor 2
P51149	RAB7A	RAB7A, member RAS oncogene family
P61006	RAB8A	RAB8A, member RAS oncogene family
B9A064	IGLL5	immunoglobulin lambda like polypeptide 5
P01031	C5	complement C5
P01834	NA	NA
P01860	NA	NA
P02746	C1QB	complement C1q B chain
P0CG06	NA	NA
O00526	UPK2	uroplakin 2
O00592	PODXL	podocalyxin like
O75351	VPS4B	vacuolar protein sorting 4 homolog B
O75955	FLOT1	flotillin 1
P04220	NA	NA
P04792	HSPB1	heat shock protein family B (small) member 1
P05023	ATP1A1	ATPase Na+/K+ transporting subunit alpha 1
P08183	ABCB1	ATP binding cassette subfamily B member 1
P08473	MME	membrane metalloendopeptidase
P08519	LPA	lipoprotein(a)
P09871	C1S	complement C1s
P07947	YES1	YES proto-oncogene 1, Src family tyrosine kinase
P12821	ACE	angiotensin I converting enzyme
P22748	CA4	carbonic anhydrase 4
P26022	PTX3	pentraxin 3
P26038	MSN	moesin
P27487	DPP4	dipeptidyl peptidase 4
P29992	GNA11	G protein subunit alpha 11
P50148	GNAQ	G protein subunit alpha q
P51148	RAB5C	RAB5C, member RAS oncogene family
P61981	YWHAG	tyrosine 3-monooxygenase/tryptophan 5-monooxygenase activation protein gamma
P62491	RAB11A	RAB11A, member RAS oncogene family
P62873	GNB1	G protein subunit beta 1
P63092	GNAS	GNAS complex locus
Q06830	PRDX1	peroxiredoxin 1
Q14254	FLOT2	flotillin 2
Q3LXA3	TKFC	triokinase and FMN cyclase
P49773	HINT1	histidine triad nucleotide binding protein 1
Q4KMQ2	ANO6	anoctamin 6
Q5VW32	BROX	BRO1 domain and CAAX motif containing
Q86XT2	VPS37D	VPS37D, ESCRT-I subunit
Q8WUM4	PDCD6IP	programmed cell death 6 interacting protein
Q92930	RAB8B	RAB8B, member RAS oncogene family
Q9H444	CHMP4B	charged multivesicular body protein 4B
Q9NP79	VTA1	vesicle trafficking 1
Q9NP85	NPHS2	NPHS2, podocin
Q15599	SLC9A3R2	SLC9A3 regulator 2
Q9NQ84	GPRC5C	G protein-coupled receptor class C group 5 member C
Q9UN37	VPS4A	vacuolar protein sorting 4 homolog A
Q9UNF0	PACSIN2	protein kinase C and casein kinase substrate in neurons 2
Q9UQB8	BAIAP2	BAI1 associated protein 2
Q9UQN3	CHMP2B	charged multivesicular body protein 2B
Q9Y6W3	CAPN7	calpain 7
O00161	SNAP23	synaptosome associated protein 23
O75841	UPK1B	uroplakin 1B
O94832	MYO1D	myosin ID

Uniprot	HGNC	Wikigene
P07948	LYN	LYN proto-oncogene, Src family tyrosine kinase
P18206	VCL	vinculin
P07225	PROS1	protein S
P51178	PLCD1	phospholipase C delta 1
P54289	CACNA2D1	calcium voltage-gated channel auxiliary subunit alpha2delta 1
P01877	IGHA2	
P63096	GNAI1	G protein subunit alpha i1
Q12907	LMAN2	lectin, mannose binding 2
Q86YQ8	CPNE8	copine 8
Q8IWA5	SLC44A2	solute carrier family 44 member 2
Q99816	TSG101	tumor susceptibility 101
O00187	MASP2	mannan binding lectin serine peptidase 2
O14773	TPP1	tripeptidyl peptidase 1
P15328	FOLR1	folate receptor 1
O75131	CPNE3	copine 3
P08637	FCGR3A	Fc fragment of IgG receptor IIIa
P62942	FKBP1A	FK506 binding protein 1A
P48509	CD151	CD151 molecule (Raph blood group)
P40925	MDH1	malate dehydrogenase 1
P55017	SLC12A3	solute carrier family 12 member 3
P61020	RAB5B	RAB5B, member RAS oncogene family
Q99102	MUC4	mucin 4, cell surface associated
Q9HD42	CHMP1A	charged multivesicular body protein 1A
Q14108	SCARB2	scavenger receptor class B member 2
Q96FZ7	CHMP6	charged multivesicular body protein 6
Q9UGM3	DMBT1	deleted in malignant brain tumors 1
P00740	F9	coagulation factor IX
P01011	SERPINA3	serpin family A member 3
P01714	IGLV3-19	
P01861	IGHG4	
P11169	SLC2A3	solute carrier family 2 member 3
Q8IY95	TMEM192	transmembrane protein 192
P12259	F5	coagulation factor V
P13498	CYBA	cytochrome b-245 alpha chain
P14543	NID1	nidogen 1
P22894	MMP8	matrix metallopeptidase 8
O00299	CLIC1	chloride intracellular channel 1
O00560	SDCBP	syndecan binding protein
P31146	CORO1A	coronin 1A
O14745	SLC9A3R1	SLC9A3 regulator 1
P31997	CEACAM8	carcinoembryonic antigen related cell adhesion molecule 8
O43451	MGAM	maltase-glucoamylase
P35858	IGFALS	insulin like growth factor binding protein acid labile subunit
O43490	PROM1	prominin 1
P52790	HK3	hexokinase 3
O43633	CHMP2A	charged multivesicular body protein 2A
P54108	CRISP3	cysteine rich secretory protein 3
O43895	XPNPEP2	X-prolyl aminopeptidase 2
P55083	MFAP4	microfibril associated protein 4
O60494	CUBN	cubilin
O60635	TSPAN1	tetraspanin 1
O75083	WDR1	WD repeat domain 1
Q03591	CFHR1	complement factor H related 1
Q06033	ITIH3	inter-alpha-trypsin inhibitor heavy chain 3
O96009	NAPSA	napsin A aspartic peptidase
Q15113	PCOLCE	procollagen C-endopeptidase enhancer
P00338	LDHA	lactate dehydrogenase A
Q7Z5L0	VMO1	vitelline membrane outer layer 1 homolog
P00558	PGK1	phosphoglycerate kinase 1
Q86UX7	FERMT3	fermitin family member 3
P00966	ASS1	argininosuccinate synthase 1
Q9HDC9	APMAP	adipocyte plasma membrane associated protein
P01009	SERPINA1	serpin family A member 1

Uniprot	HGNC	Wikigene
P01871	IGHM	
P01876	IGHA1	
P02768	ALB	albumin
P04075	ALDOA	aldolase, fructose-bisphosphate A
P04216	THY1	Thy-1 cell surface antigen
P04406	GAPDH	glyceraldehyde-3-phosphate dehydrogenase
P04899	GNAI2	G protein subunit alpha i2
P05062	ALDOB	aldolase, fructose-bisphosphate B
P05090	APOD	apolipoprotein D
P06733	ENO1	enolase 1
P07195	LDHB	lactate dehydrogenase B
P07355	ANXA2	annexin A2
P07737	PFN1	profilin 1
P00918	CA2	carbonic anhydrase 2
P07900	HSP90AA1	heat shock protein 90 alpha family class A member 1
P08107	NA	NA
P08133	ANXA6	annexin A6
P08758	ANXA5	annexin A5
P09211	GSTP1	glutathione S-transferase pi 1
P09525	ANXA4	annexin A4
P0CG48	UBC	ubiquitin C
P10909	CLU	clusterin
P11142	HSPA8	heat shock protein family A (Hsp70) member 8
P13987	CD59	CD59 molecule (CD59 blood group)
P20339	RAB5A	RAB5A, member RAS oncogene family
P14618	PKM	pyruvate kinase, muscle
P15144	ANPEP	alanyl aminopeptidase, membrane
P15311	EZR	ezrin
P15941	MUC1	mucin 1, cell surface associated
P16444	DPEP1	dipeptidase 1
P19440	GGT1	gamma-glutamyltransferase 1
P27105	STOM	stomatin
P29401	TKT	transketolase
P30626	SRI	sorcin
P31949	S100A11	S100 calcium binding protein A11
P35241	RDX	radixin
P50995	ANXA11	annexin A11
P53990	IST1	IST1, ESCRT-III associated factor
P60174	TPI1	triosephosphate isomerase 1
P60660	MYL6	myosin light chain 6
Q16827	PTPRO	protein tyrosine phosphatase, receptor type O
P60709	ACTB	actin beta
P60953	CDC42	cell division cycle 42
Q8NI35	PATJ	PATJ, crumbs cell polarity complex component
P61586	RHOA	ras homolog family member A
P62158	NA	NA
P62937	PPIA	peptidylprolyl isomerase A
P63000	RAC1	Rac family small GTPase 1
Q96DG6	CMBL	carboxymethylenebutenolidase homolog
P63104	YWHAZ	tyrosine 3-monooxygenase/tryptophan 5-monooxygenase activation protein zeta
P98164	LRP2	LDL receptor related protein 2
Q08380	LGALS3BP	galectin 3 binding protein
Q12913	PTPRJ	protein tyrosine phosphatase, receptor type J
Q14344	GNA13	G protein subunit alpha 13
Q9UKS6	PACSIN3	protein kinase C and casein kinase substrate in neurons 3
Q5T2W1	PDZK1	PDZ domain containing 1
Q7LBR1	CHMP1B	charged multivesicular body protein 1B
Q8WV92	MITD1	microtubule interacting and trafficking domain containing 1
Q93088	BHMT	betaine--homocysteine S-methyltransferase
Q9H4M9	EHD1	EH domain containing 1
P02671	FGA	fibrinogen alpha chain
P01779	NA	NA
P04217	A1BG	alpha-1-B glycoprotein

Uniprot	HGNC	Wikigene
Q10588	BST1	bone marrow stromal cell antigen 1
Q96PD5	PGLYRP2	peptidoglycan recognition protein 2
O60437	PPL	periplakin
O75340	PDCD6	programmed cell death 6
P00441	SOD1	superoxide dismutase 1
P13688	CEACAM1	carcinoembryonic antigen related cell adhesion molecule 1
P01133	EGF	epidermal growth factor
P02760	AMBP	alpha-1-microglobulin/bikunin precursor
P68371	TUBB4B	tubulin beta 4B class IVb
Q13621	SLC12A1	solute carrier family 12 member 1
Q8N271	PROM2	prominin 2
Q8TE68	EPS8L1	EPS8 like 1
Q9Y287	ITM2B	integral membrane protein 2B
P08263	GSTA1	glutathione S-transferase alpha 1
P01042	KNG1	kininogen 1
P01833	PIGR	polymeric immunoglobulin receptor
P02649	APOE	apolipoprotein E
P46940	IQGAP1	IQ motif containing GTPase activating protein 1
P0CG05	NA	NA
Q9UHI7	SLC23A1	solute carrier family 23 member 1
P35579	MYH9	myosin heavy chain 9
P61026	RAB10	RAB10, member RAS oncogene family
P07911	UMOD	uromodulin
P62879	GNB2	G protein subunit beta 2
Q695T7	SLC6A19	solute carrier family 6 member 19
P60981	DSTN	destrin, actin depolymerizing factor
O00602	FCN1	
O75636	FCN3	ficolin 3
O95498	VNN2	vanin 2
P00739	HPR	haptoglobin-related protein
Q01518	CAP1	cyclase associated actin cytoskeleton regulatory protein 1
P01617	NA	NA
P02748	C9	complement C9
P04839	CYBB	cytochrome b-245 beta chain
P05107	ITGB2	integrin subunit beta 2
P07357	C8A	complement C8 alpha chain
P08311	CTSG	cathepsin G
Q96CF2	CHMP4C	charged multivesicular body protein 4C
P08575	PTPRC	protein tyrosine phosphatase, receptor type C
P10643	C7	complement C7
P11215	ITGAM	integrin subunit alpha M
P13671	C6	complement C6
P15153	RAC2	Rac family small GTPase 2
P28676	GCA	grancalcin
P36873	PPP1CC	protein phosphatase 1 catalytic subunit gamma
P36955	SERPINF1	serpin family F member 1
P49913	NA	NA
P80511	S100A12	S100 calcium binding protein A12
P80748	IGLV3-21	
Q14624	ITIH4	inter-alpha-trypsin inhibitor heavy chain family member 4
P07339	CTSD	cathepsin D
Q15485	FCN2	ficolin 2
Q14247	CTTN	cortactin
Q99536	VAT1	vesicle amine transport 1
P19971	TYMP	thymidine phosphorylase
O75264	SMIM24	small integral membrane protein 24
P07858	CTSB	cathepsin B
Q8WW52	FAM151A	family with sequence similarity 151 member A
O43866	CD5L	CD5 molecule like
P01024	C3	complement C3
P01623	NA	NA
P01857	IGHG1	
P02652	APOA2	apolipoprotein A2

Uniprot	HGNC	Wikigene
P02751	FN1	fibronectin 1
P02790	HPX	hemopexin
P04004	VTN	vitronectin
P04275	VWF	von Willebrand factor
P06727	APOA4	apolipoprotein A4
P19823	ITIH2	inter-alpha-trypsin inhibitor heavy chain 2
Q00610	CLTC	clathrin heavy chain
P62070	RRAS2	RAS related 2
P01859	IGHG2	
Q09666	AHNAK	AHNAK nucleoprotein
P04083	ANXA1	annexin A1
Q9BYF1	ACE2	angiotensin I converting enzyme 2
P06396	GSN	gelsolin
Q9Y696	CLIC4	chloride intracellular channel 4
P08195	SLC3A2	solute carrier family 3 member 2
P17931	LGALS3	galectin 3
P21926	CD9	CD9 molecule
P04114	APOB	apolipoprotein B
P12429	ANXA3	annexin A3

Cluster 2

Uniprot	HGNC	Wikigene
P32119	PRDX2	peroxiredoxin 2
Q96NY7	CLIC6	chloride intracellular channel 6
A0PJK1	SLC5A10	solute carrier family 5 member 10
O43175	PHGDH	phosphoglycerate dehydrogenase
O60361	NA	NA
O60658	PDE8A	phosphodiesterase 8A
O75368	SH3BGRL	SH3 domain binding glutamate rich protein like
O95630	STAMBP	STAM binding protein
P05413	FABP3	fatty acid binding protein 3
P07108	DBI	diazepam binding inhibitor, acyl-CoA binding protein
P07148	FABP1	fatty acid binding protein 1
P07288	KLK3	kallikrein related peptidase 3
Q93050	ATP6V0A1	ATPase H+ transporting V0 subunit a1
P09936	UCHL1	ubiquitin C-terminal hydrolase L1
P10301	RRAS	RAS related
Q99571	P2RX4	purinergic receptor P2X 4
P10768	ESD	esterase D
Q9H3R2	MUC13	mucin 13, cell surface associated
P11586	MTHFD1	methylenetetrahydrofolate dehydrogenase, cyclohydrolase and formyltetrahydrofolate synthetase 1
P12277	CKB	creatine kinase B
P15121	AKR1B1	aldo-keto reductase family 1 member B
O14908	GIPC1	GIPC PDZ domain containing family member 1
P15309	ACPP	acid phosphatase, prostate
P21266	GSTM3	glutathione S-transferase mu 3
P30039	PBLD	phenazine biosynthesis like protein domain containing
Q9NUP9	LIN7C	lin-7 homolog C, crumbs cell polarity complex component
P31689	DNAJA1	DnaJ heat shock protein family (Hsp40) member A1
P36405	ARL3	ADP ribosylation factor like GTPase 3
P36543	ATP6V1E1	ATPase H+ transporting V1 subunit E1
P50502	ST13	ST13, Hsp70 interacting protein
P52209	PGD	phosphogluconate dehydrogenase
P54652	HSPA2	heat shock protein family A (Hsp70) member 2
Q00796	SORD	sorbitol dehydrogenase
Q03154	ACY1	aminoacylase 1
Q15185	PTGES3	prostaglandin E synthase 3
Q3SXY8	ARL13B	ADP ribosylation factor like GTPase 13B
Q4U2R8	SLC22A6	solute carrier family 22 member 6
Q53GD3	SLC44A4	solute carrier family 44 member 4
Q6ZQN7	SLCO4C1	solute carrier organic anion transporter family member 4C1
Q6ZS17	RIPOR1	RHO family interacting cell polarization regulator 1
Q7L5L3	GDPD3	glycerophosphodiester phosphodiesterase domain containing 3

Uniprot	HGNC	Wikigene
Q7L7X3	TAOK1	TAO kinase 1
Q7L9L4	MOB1B	MOB kinase activator 1B
Q7Z2W4	ZC3HAV1	zinc finger CCCH-type containing, antiviral 1
Q7Z4W1	DCXR	dicarbonyl and L-xylulose reductase
Q8N3R9	MPP5	membrane palmitoylated protein 5
Q8N4C8	MINK1	misshapen like kinase 1
Q8NFU3	TSTD1	thiosulfate sulfurtransferase like domain containing 1
Q8TAD7	C12orf75	chromosome 12 open reading frame 75
Q96J02	ITCH	itchy E3 ubiquitin protein ligase
Q9BZV1	UBXN6	UBX domain protein 6
Q9H190	SDCBP2	syndecan binding protein 2
Q9H7P6	MVB12B	multivesicular body subunit 12B
Q9NXU5	ARL15	ADP ribosylation factor like GTPase 15
Q9UBP0	SPAST	spastin
Q9UI12	ATP6V1H	ATPase H+ transporting V1 subunit H
Q9Y617	PSAT1	phosphoserine aminotransferase 1
Q9Y6R1	SLC4A4	solute carrier family 4 member 4
Q16775	HAGH	hydroxyacylglutathione hydrolase
O60884	DNAJA2	DnaJ heat shock protein family (Hsp40) member A2
O95497	VNN1	vanin 1
P05981	HPN	hepsin
P42685	FRK	fyn related Src family tyrosine kinase
Q5IJ48	CRB2	crumbs 2, cell polarity complex component
Q6P1N0	CC2D1A	coiled-coil and C2 domain containing 1A
Q99835	SMO	smoothened, frizzled class receptor
Q9NRX4	PHPT1	phosphohistidine phosphatase 1
Q9NZV1	CRIM1	cysteine rich transmembrane BMP regulator 1
A6NEC2	NA	NA
O60701	UGDH	UDP-glucose 6-dehydrogenase
O75110	ATP9A	ATPase phospholipid transporting 9A (putative)
O75348	ATP6V1G1	ATPase H+ transporting V1 subunit G1
O94760	DDAH1	dimethylarginine dimethylaminohydrolase 1
O95336	PGLS	6-phosphogluconolactonase
O95394	PGM3	phosphoglucomutase 3
O95716	RAB3D	RAB3D, member RAS oncogene family
P01111	NRAS	NRAS proto-oncogene, GTPase
P07384	CAPN1	calpain 1
P08118	MSMB	microseminoprotein beta
P09417	QDPR	quinoid dihydropteridine reductase
P09960	LTA4H	leukotriene A4 hydrolase
P11766	ADH5	alcohol dehydrogenase 5 (class III), chi polypeptide
P13489	RNH1	ribonuclease/angiogenin inhibitor 1
P13796	LCP1	lymphocyte cytosolic protein 1
P16870	CPE	carboxypeptidase E
P17342	NPR3	natriuretic peptide receptor 3
P20337	RAB3B	RAB3B, member RAS oncogene family
P25311	AZGP1	alpha-2-glycoprotein 1, zinc-binding
P27348	YWHAQ	tyrosine 3-monooxygenase/tryptophan 5-monooxygenase activation protein theta
P28838	LAP3	leucine aminopeptidase 3
P28907	CD38	CD38 molecule
P30046	DDT	D-dopachrome tautomerase
P30085	CMPK1	cytidine/uridine monophosphate kinase 1
P30153	PPP2R1A	protein phosphatase 2 scaffold subunit Aalpha
P31150	GDI1	GDP dissociation inhibitor 1
P31939	ATIC	5-aminoimidazole-4-carboxamide ribonucleotide formyltransferase/IMP cyclohydrolase
P33176	KIF5B	kinesin family member 5B
P35237	SERPINB6	serpin family B member 6
P36969	GPX4	glutathione peroxidase 4
P48637	GSS	glutathione synthetase
P49189	ALDH9A1	aldehyde dehydrogenase 9 family member A1
P49327	FASN	fatty acid synthase
P50990	CCT8	chaperonin containing TCP1 subunit 8
P51159	RAB27A	RAB27A, member RAS oncogene family

Uniprot	HGNC	Wikigene
P52565	ARHGDIA	Rho GDP dissociation inhibitor alpha
P52788	SMS	spermine synthase
P53396	ACLY	ATP citrate lyase
P62140	PPP1CB	protein phosphatase 1 catalytic subunit beta
P63027	VAMP2	vesicle associated membrane protein 2
P68402	PAFAH1B2	platelet activating factor acetylhydrolase 1b catalytic subunit 2
P78417	GSTO1	glutathione S-transferase omega 1
P80297	MT1X	metallothionein 1X
Q04609	FOLH1	folate hydrolase 1
Q04760	GLO1	glyoxalase I
Q12765	SCRN1	secernin 1
Q14240	EIF4A2	eukaryotic translation initiation factor 4A2
Q14894	CRYM	crystallin mu
Q687X5	STEAP4	STEAP4 metalloreductase
Q6IWH7	ANO7	anoctamin 7
Q6UXI9	NPNT	nephronectin
Q7Z404	TMC4	transmembrane channel like 4
Q8IYJ3	SYTL1	synaptotagmin like 1
Q8N9U0	TC2N	tandem C2 domains, nuclear
Q96C24	SYTL4	synaptotagmin like 4
Q96IU4	ABHD14B	abhydrolase domain containing 14B
Q9BV36	MLPH	melanophilin
Q9BWD1	ACAT2	acetyl-CoA acetyltransferase 2
Q9BZQ8	FAM129A	family with sequence similarity 129 member A
Q9H0U4	RAB1B	RAB1B, member RAS oncogene family
Q9H4A4	RNPEP	arginyl aminopeptidase
Q9HBG4	ATP6V0A4	ATPase H+ transporting V0 subunit a4
Q9NQR4	NIT2	nitrilase family member 2
Q9NQX5	NPDC1	neural proliferation, differentiation and control 1
Q9NR45	NANS	N-acetylneuraminate synthase
Q9UBQ7	GRHPR	glyoxylate and hydroxypyruvate reductase
Q9UL25	RAB21	RAB21, member RAS oncogene family
Q9Y2S2	CRYL1	crystallin lambda 1
Q9Y3E7	CHMP3	charged multivesicular body protein 3
Q9Y3R5	DOPEY2	dopey family member 2
P21796	VDAC1	voltage dependent anion channel 1
Q8IX04	UEVLD	UEV and lactate/malate dehyrogenase domains
Q9BRK3	MXRA8	matrix remodeling associated 8
O43488	AKR7A2	aldo-keto reductase family 7 member A2
O75347	TBCA	tubulin folding cofactor A
P00390	GSR	glutathione-disulfide reductase
P06744	GPI	glucose-6-phosphate isomerase
P07437	TUBB	tubulin beta class I
P08238	HSP90AB1	heat shock protein 90 alpha family class B member 1
P13639	EEF2	eukaryotic translation elongation factor 2
P17174	GOT1	glutamic-oxaloacetic transaminase 1
P20151	KLK2	kallikrein related peptidase 2
P21281	ATP6V1B2	ATPase H+ transporting V1 subunit B2
P23526	AHCY	adenosylhomocysteinase
Q13228	SELENBP1	selenium binding protein 1
Q8N392	ARHGAP18	Rho GTPase activating protein 18
O00401	WASL	Wiskott-Aldrich syndrome like
Q99497	PARK7	Parkinsonism associated deglycase
O43795	MYO1B	myosin IB
P20711	DDC	dopa decarboxylase
Q9UM54	MYO6	myosin VI
P43353	ALDH3B1	aldehyde dehydrogenase 3 family member B1
P54793	ARSF	arylsulfatase F
P61421	ATP6V0D1	ATPase H+ transporting V0 subunit d1
P98172	EFNB1	ephrin B1
Q05655	PRKCD	protein kinase C delta
Q8WWT9	SLC13A3	solute carrier family 13 member 3
Q969P0	IGSF8	immunoglobulin superfamily member 8

Uniprot	HGNC	Wikigene
Q9NV96	TMEM30A	transmembrane protein 30A
P21964	COMT	catechol-O-methyltransferase
P22314	UBA1	ubiquitin like modifier activating enzyme 1
P30044	PRDX5	peroxiredoxin 5
P54920	NAPA	NSF attachment protein alpha
P61019	RAB2A	RAB2A, member RAS oncogene family
P62820	RAB1A	RAB1A, member RAS oncogene family
P68363	TUBA1B	tubulin alpha 1b
Q9Y490	TLN1	talin 1
O75936	BBOX1	gamma-butyrobetaine hydroxylase 1
P61106	RAB14	RAB14, member RAS oncogene family
P05186	ALPL	alkaline phosphatase, liver/bone/kidney
Q9Y6W5	WASF2	WAS protein family member 2
P00352	ALDH1A1	aldehyde dehydrogenase 1 family member A1
P08582	MELTF	melanotransferrin
P09467	FBP1	fructose-bisphosphatase 1
P10599	TXN	thioredoxin
P15313	ATP6V1B1	ATPase H+ transporting V1 subunit B1
P21695	GPD1	glycerol-3-phosphate dehydrogenase 1
P30041	PRDX6	peroxiredoxin 6
P30043	BLVRB	biliverdin reductase B
P50053	KHK	ketohexokinase
P50895	BCAM	basal cell adhesion molecule (Lutheran blood group)
O95398	RAPGEF3	Rap guanine nucleotide exchange factor 3
P55072	VCP	valosin containing protein
Q14914	PTGR1	prostaglandin reductase 1
Q9BUT1	BDH2	3-hydroxybutyrate dehydrogenase 2
Q9UGT4	SUSD2	sushi domain containing 2
Q10589	BST2	bone marrow stromal cell antigen 2
Q15847	ADIRF	adipogenesis regulatory factor
P61158	ACTR3	ARP3 actin related protein 3 homolog
Q9UBR2	CTSZ	cathepsin Z
Q8N6Q3	CD177	CD177 molecule
O43653	PSCA	prostate stem cell antigen
P08134	RHOC	ras homolog family member C
Q8WTX9	ZDHHC1	zinc finger DHHC-type containing 1
Q9H0W9	C11orf54	chromosome 11 open reading frame 54

Cluster 3

Uniprot	HGNC	Wikigene
P02730	SLC4A1	solute carrier family 4 member 1 (Diego blood group)
O75631	UPK3A	uroplakin 3A
O14818	PSMA7	proteasome subunit alpha 7
P01602	IGKV1-5	
P08514	ITGA2B	integrin subunit alpha 2b
P25786	PSMA1	proteasome subunit alpha 1
P25787	PSMA2	proteasome subunit alpha 2
P28070	PSMB4	proteasome subunit beta 4
P28074	PSMB5	proteasome subunit beta 5
P43652	AFM	afamin
P69891	HBG1	hemoglobin subunit gamma 1
Q13885	TUBB2A	tubulin beta 2A class IIa
Q3YBM2	TMEM176B	transmembrane protein 176B
Q8IVW8	SPNS2	sphingolipid transporter 2
Q8WWI5	SLC44A1	solute carrier family 44 member 1
Q99436	PSMB7	proteasome subunit beta 7
Q9NUN5	LMBRD1	LMBR1 domain containing 1
P05106	ITGB3	integrin subunit beta 3
P00568	AK1	adenylate kinase 1
P00915	CA1	carbonic anhydrase 1
P01889	HLA-B	major histocompatibility complex, class I, B
P02549	SPTA1	spectrin alpha, erythrocytic 1
P02724	GYPA	glycophorin A (MNS blood group)

Uniprot	HGNC	Wikigene
P04632	CAPNS1	calpain small subunit 1
P06681	C2	complement C2
P07738	BPGM	bisphosphoglycerate mutase
P11171	EPB41	erythrocyte membrane protein band 4.1
P11277	SPTB	spectrin beta, erythrocytic
P13716	ALAD	aminolevulinate dehydratase
P13798	APEH	acylaminoacyl-peptide hydrolase
P15259	PGAM2	phosphoglycerate mutase 2
P16157	ANK1	ankyrin 1
P16452	EPB42	erythrocyte membrane protein band 4.2
P26447	S100A4	S100 calcium binding protein A4
P37840	SNCA	synuclein alpha
Q00013	MPP1	membrane palmitoylated protein 1
Q14019	COTL1	coactosin like F-actin binding protein 1
Q15102	PAFAH1B3	platelet activating factor acetylhydrolase 1b catalytic subunit 3
Q92530	PSMF1	proteasome inhibitor subunit 1
P02042	HBD	hemoglobin subunit delta
P04040	CAT	catalase
B0FP48	UPK3BL1	uroplakin 3B like 1
O00151	PDLIM1	PDZ and LIM domain 1
O14936	CASK	calcium/calmodulin dependent serine protein kinase
O15195	VILL	villin like
P11166	SLC2A1	solute carrier family 2 member 1
O15231	ZNF185	zinc finger protein 185 with LIM domain
O43520	ATP8B1	ATPase phospholipid transporting 8B1
O60493	SNX3	sorting nexin 3
O60716	CTNND1	catenin delta 1
O75355	ENTPD3	ectonucleoside triphosphate diphosphohydrolase 3
P62826	RAN	RAN, member RAS oncogene family
O75369	FLNB	filamin B
O95833	CLIC3	chloride intracellular channel 3
P00533	EGFR	epidermal growth factor receptor
P02749	APOH	apolipoprotein H
P04626	ERBB2	erb-b2 receptor tyrosine kinase 2
P14923	JUP	junction plakoglobin
P16144	ITGB4	integrin subunit beta 4
P17301	ITGA2	integrin subunit alpha 2
P18283	GPX2	glutathione peroxidase 2
P18463	HLA-B	major histocompatibility complex, class I, B
P18564	ITGB6	integrin subunit beta 6
P20020	ATP2B1	ATPase plasma membrane Ca2+ transporting 1
P21589	NT5E	5'-nucleotidase ecto
P23229	ITGA6	integrin subunit alpha 6
P29692	EEF1D	eukaryotic translation elongation factor 1 delta
P35573	AGL	amylo-alpha-1, 6-glucosidase, 4-alpha-glucanotransferase
P50897	PPT1	palmitoyl-protein thioesterase 1
P52895	AKR1C2	aldo-keto reductase family 1 member C2
P62136	PPP1CA	protein phosphatase 1 catalytic subunit alpha
P78310	CXADR	CXADR, Ig-like cell adhesion molecule
Q04828	AKR1C1	aldo-keto reductase family 1 member C1
Q12846	STX4	syntaxin 4
Q14126	DSG2	desmoglein 2
Q86X29	LSR	lipolysis stimulated lipoprotein receptor
Q92599	SEPT8	septin 8
Q92734	TFG	TRK-fused gene
Q96QK1	VPS35	VPS35, retromer complex component
Q99829	CPNE1	copine 1
Q9H4G0	EPB41L1	erythrocyte membrane protein band 4.1 like 1
Q9HCH5	SYTL2	synaptotagmin like 2
Q9P2B2	PTGFRN	prostaglandin F2 receptor inhibitor
Q9UHD8	SEPT9	septin 9
Q9UHN6	TMEM2	transmembrane protein 2
Q9Y5Y6	ST14	suppression of tumorigenicity 14

Uniprot	HGNC	Wikigene
Q9Y5Z4	HEBP2	heme binding protein 2
Q9Y624	F11R	F11 receptor
P35221	CTNNA1	catenin alpha 1
P35542	SAA4	serum amyloid A4, constitutive
P08727	KRT19	keratin 19
P09758	TACSTD2	tumor associated calcium signal transducer 2
P02743	APCS	amyloid P component, serum
P08729	KRT7	keratin 7
P25788	PSMA3	proteasome subunit alpha 3
P28066	PSMA5	proteasome subunit alpha 5
P35222	CTNNB1	catenin beta 1
P60900	PSMA6	proteasome subunit alpha 6
P28072	PSMB6	proteasome subunit beta 6
P16070	CD44	CD44 molecule (Indian blood group)

Table S4. Top 50 proteins in bladder and ureter exosomes, together with the top 5 associated pathways.

Upregulated in Bladder

Uniprot Accession	HGNC Symbol	Wikigene description	Log-fold change bladder vs ureter	p-value
O15393	TMPRSS2	transmembrane serine protease 2	5.332022976	0.00E+00
O15162	PLSCR1	phospholipid scramblase 1	3.872777375	1.21E-13
Q16651	PRSS8	serine protease 8	4.271527203	1.18E-06
P14384	CPM	carboxypeptidase M	3.213859622	1.18E-06
P15328	FOLR1	folate receptor alpha	4.373879406	5.61E-06
P17927	CR1	complement C3b/C4b receptor 1 (Knops blood group)	4.654277339	5.03E-04
P61204	ARF3	ADP ribosylation factor 3	3.110965137	7.36E-04
Q8TE68	EPS8L1	EPS8 like 1	3.453753773	4.13E-03
P08174	CD55	CD55 molecule (Cromer blood group)	3.311183997	6.31E-03
Q9Y6E0	STK24	serine/threonine kinase 24	3.296502539	6.31E-03
P42330	AKR1C3	aldo-keto reductase family 1 member C3	3.287463908	6.31E-03
Q13277	STX3	syntaxin 3	3.276818395	6.31E-03
O96009	NAPSA	napsin A aspartic peptidase	3.519161847	6.74E-03
Q9BUL8	PDCD10	programmed cell death 10	3.510793706	6.80E-03
Q9NRX4	PHPT1	phosphohistidine phosphatase 1	3.555183927	7.56E-03
Q9BRK3	MXRA8	matrix remodeling associated 8	3.2507557	7.56E-03
P16152	CBR1	carbonyl reductase 1	4.03883054	7.61E-03
Q9H6S3	EPS8L2	EPS8 like 2	3.771438019	7.61E-03
Q9H2K8	TAOK3	TAO kinase 3	3.537583678	7.61E-03
Q8IWA5	SLC44A2	solute carrier family 44 member 2	3.306258762	7.61E-03
Q8NFJ5	GPRC5A	G protein-coupled receptor class C group 5 member A	3.182127031	7.61E-03
Q9UBV8	PEF1	penta-EF-hand domain containing 1	3.155459618	8.64E-03
Q96RF0	SNX18	sorting nexin 18	3.241519186	9.19E-03
P04216	THY1	Thy-1 cell surface antigen	3.154643777	9.19E-03
P11234	RALB	RAS like proto-oncogene B	3.213022115	1.11E-02
P04083	ANXA1	annexin A1	3.737734985	1.70E-02
O75131	CPNE3	copine 3	3.519118176	1.75E-02
Q9UHR4	BAIAP2L1	BAI1 associated protein 2 like 1	3.853405556	1.88E-02
P07948	LYN	LYN proto-oncogene, Src family tyrosine kinase	3.312438806	3.46E-02
Q9HD42	CHMP1A	charged multivesicular body protein 1A	3.424915151	3.71E-02
Q8N271	PROM2	prominin 2	3.084670129	4.34E-02
Q15286	RAB35	RAB35, member RAS oncogene family	3.052396307	4.77E-02
Q9H4G4	GLIPR2	GLI pathogenesis related 2	3.568686053	4.78E-02
P51153	RAB13	RAB13, member RAS oncogene family	3.397455794	5.70E-02
P50395	GDI2	GDP dissociation inhibitor 2	3.084921633	6.74E-02
Q6UX06	OLFM4	olfactomedin 4	3.043128513	6.74E-02
P06396	GSN	gelsolin	3.059297045	6.89E-02
Q9UKS6	PACSIN3	protein kinase C and casein kinase substrate in neurons 3	3.069927262	6.95E-02
Q12929	EPS8	epidermal growth factor receptor pathway substrate 8	3.205689918	7.40E-02
P15309	ACPP	acid phosphatase, prostate	4.168572518	7.88E-02
P12277	CKB	creatine kinase B	3.840854082	7.88E-02
Q92817	EVPL	envoplakin	3.418807832	7.88E-02
P21796	VDAC1	voltage dependent anion channel 1	3.074898832	7.88E-02
P07288	KLK3	kallikrein related peptidase 3	3.39845216	8.02E-02
P68032	ACTC1	actin alpha cardiac muscle 1	4.823491673	1.02E-01
P61006	RAB8A	RAB8A, member RAS oncogene family	3.050542447	1.04E-01
Q92597	NDRG1	N-myc downstream regulated 1	3.081093985	1.53E-01
Q00796	SORD	sorbitol dehydrogenase	3.247958343	1.67E-01
P06702	S100A9	S100 calcium binding protein A9	3.137495401	1.67E-01
P02788	LTF	lactotransferrin	3.162710379	2.18E-01

Upregulated in ureter

Uniprot Accession	HGNC Symbol	Wikigene description	Log-fold change bladder vs ureter	p-value
P02730	SLC4A1	solute carrier family 4 member 1 (Diego blood group)	-5.091758941	5.61E-06
P02042	HBD	hemoglobin subunit delta	-3.839714599	1.70E-01
P69905	HBA2	hemoglobin subunit alpha 2	-3.512491284	7.88E-02
P09758	TACSTD2	tumor associated calcium signal transducer 2	-3.40024048	7.19E-02
P68871	HBB	hemoglobin subunit beta	-2.902783114	1.10E-01
P04040	CAT	catalase	-2.5737639	1.70E-01
P08729	KRT7	keratin 7	-2.550785708	8.99E-02
P35221	CTNNA1	catenin alpha 1	-2.53296626	1.10E-01
P35222	CTNNB1	catenin beta 1	-2.404904638	1.02E-01
P00915	CA1	carbonic anhydrase 1	-2.224960479	1.95E-01
P11166	SLC2A1	solute carrier family 2 member 1	-2.135757355	1.87E-01
P16070	CD44	CD44 molecule (Indian blood group)	-2.069083625	1.70E-01
P08603	CFH	complement factor H	-1.824850373	4.05E-01
O60716	CTNND1	catenin delta 1	-1.781190522	2.09E-01
P08727	KRT19	keratin 19	-1.559256613	4.05E-01
P00734	F2	coagulation factor II, thrombin	-1.510458907	4.05E-01
P16157	ANK1	ankyrin 1	-1.48461703	3.33E-01
P16452	EPB42	erythrocyte membrane protein band 4.2	-1.455897648	3.15E-01
P50897	PPT1	palmitoyl-protein thioesterase 1	-1.415230125	3.10E-01
P16403	HIST1H1C	histone cluster 1 H1 family member c	-1.317511992	3.10E-01
O60814	HIST1H2BK	histone cluster 1 H2B family member k	-1.30679313	4.66E-01
P28066	PSMA5	proteasome subunit alpha 5	-1.302863974	3.10E-01
P14923	JUP	junction plakoglobin	-1.267947208	3.15E-01
P11277	SPTB	spectrin beta, erythrocytic	-1.247226599	3.15E-01
Q9Y624	F11R	F11 receptor	-1.232418837	3.15E-01
P30101	PDIA3	protein disulfide isomerase family A member 3	-1.226120833	3.15E-01
P02647	APOA1	apolipoprotein A1	-1.218075234	4.61E-01
P35542	SAA4	serum amyloid A4, constitutive	-1.211062736	4.05E-01
Q9P2B2	PTGFRN	prostaglandin F2 receptor inhibitor	-1.172653198	3.10E-01
Q04828	AKR1C1	aldo-keto reductase family 1 member C1	-1.159684738	3.10E-01
Q14764	MVP	major vault protein	-1.155442981	5.05E-01
P25786	PSMA1	proteasome subunit alpha 1	-1.104711631	3.10E-01
P08514	ITGA2B	integrin subunit alpha 2b	-1.085291123	3.15E-01
P02749	APOH	apolipoprotein H	-1.084973678	3.10E-01
Q9HCH5	SYTL2	synaptotagmin like 2	-1.082902032	3.15E-01
P07738	BPGM	bisphosphoglycerate mutase	-1.076737785	3.16E-01
P01765	IGHV3-23	Immunoglobulin heavy variable 3-23	-1.060246106	4.47E-01
P25788	PSMA3	proteasome subunit alpha 3	-1.042710607	3.10E-01
P13716	ALAD	aminolevulinate dehydratase	-1.040186869	3.10E-01
Q5QNW6	HIST2H2BF	histone cluster 2 H2B family member f	-1.036039189	6.18E-01
P05106	ITGB3	integrin subunit beta 3	-1.009754865	4.57E-01
P01889	HLA-B	major histocompatibility complex, class I, B	-1.006559147	3.11E-01
Q14651	PLS1	plastin 1	-0.948592148	4.40E-01
P69891	HBG1	hemoglobin subunit gamma 1	-0.914925984	4.05E-01
P02743	APCS	amyloid P component, serum	-0.89321571	5.12E-01
P62826	RAN	RAN, member RAS oncogene family	-0.892609716	4.28E-01
Q9BQE3	TUBA1C	tubulin alpha 1c	-0.877853021	4.53E-01
P05546	SERPIND1	serpin family D member 1	-0.782907935	6.80E-01
P00738	HP	haptoglobin	-0.770914875	6.17E-01
P16401	HIST1H1B	histone cluster 1 H1 family member b	-0.763137593	5.81E-01

Upregulated in Bladder (Annotation (pathway/process)

	XD-score	Fisher q-value	Gene set size	'athway siz	)verlap siz	Overlapping proteins
hsa00740:Riboflavin me	1.101230192	0.282775924	47	16	2	PHPT1 ACPP
hsa00051:Fructose and	0.521684737	0.599738447	47	33	2	PHPT1 SORD
hsa00590:Arachidonic a	0.322384038	0.963939136	47	52	2	AKR1C3 CBR1
hsa04610:Complement	0.237099757	1	47	69	2	CR1 CD55

hsa04130:SNARE intera	0.233373049	1	47	35	1	STX3

Upregulated in ureter (Annotation (pathway/process)

	XD-score	Fisher q-value	Gene set size	'athway siz	)verlap siz	Overlapping proteins
hsa03050:Proteasome	0.572861105	0.14462055	46	43	3	PSMA1 PSMA3 PSMA5
hsa05144:Malaria	0.507454128	0.14462055	46	48	3	HBA2 HBB HBD
hsa05412:Arrhythmoger	0.438104813	0.122781126	46	73	4	CTNNA1 CTNNB1 ITGA2B JUP
hsa00910:Nitrogen meta	0.373525557	1	46	21	1	CA1
hsa04610:Complement	0.336258476	0.228163584	46	69	3	F2 SERPIND1 CFH

Table S5. Proteins showing significant differential expression of exosomal proteins between bladder and ureter.

Uniprot Accession	HGNC Symbol	Wikigene description	Bladder_logfc	Bladder_p.value
O15393	TMPRSS2*	transmembrane serine protease 2	5.332022976	0.00E+00
O15162	PLSCR1	phospholipid scramblase 1	3.872777375	1.21E-13
P14384	CPM	carboxypeptidase M	3.213859622	1.18E-06
Q16651	PRSS8	serine protease 8	4.271527203	1.18E-06
P15328	FOLR1*	folate receptor alpha	4.373879406	5.61E-06
P02730	SLC4A1	solute carrier family 4 member 1 (Diego blood group)	-5.091758941	5.61E-06
P17927	CR1	complement C3b/C4b receptor 1 (Knops blood group)	4.654277339	5.03E-04
P61204	ARF3	ADP ribosylation factor 3	3.110965137	7.36E-04
Q8TE68	EPS8L1	EPS8 like 1	3.453753773	4.13E-03
P42330	AKR1C3*	aldo-keto reductase family 1 member C3	3.287463908	6.31E-03
P08174	CD55	CD55 molecule (Cromer blood group)	3.311183997	6.31E-03
Q13277	STX3	syntaxin 3	3.276818395	6.31E-03
Q9Y6E0	STK24*	serine/threonine kinase 24	3.296502539	6.31E-03
O96009	NAPSA	napsin A aspartic peptidase	3.519161847	6.74E-03
Q9BUL8	PDCD10	programmed cell death 10	3.510793706	6.80E-03
Q10588	BST1	bone marrow stromal cell antigen 1	2.813923039	7.56E-03
P04080	CSTB*	cystatin B	2.904540826	7.56E-03
Q9BRK3	MXRA8	matrix remodeling associated 8	3.2507557	7.56E-03
Q9NRX4	PHPT1*	phosphohistidine phosphatase 1	3.555183927	7.56E-03
Q9H2K8	TAOK3	TAO kinase 3	3.537583678	7.61E-03
Q8IWA5	SLC44A2	solute carrier family 44 member 2	3.306258762	7.61E-03
Q8NFJ5	GPRC5A*	G protein-coupled receptor class C group 5 member A	3.182127031	7.61E-03
Q9H6S3	EPS8L2	EPS8 like 2	3.771438019	7.61E-03
P16152	CBR1	carbonyl reductase 1	4.03883054	7.61E-03
Q9UBV8	PEF1	penta-EF-hand domain containing 1	3.155459618	8.64E-03
P04216	THY1	Thy-1 cell surface antigen	3.154643777	9.19E-03
Q96RF0	SNX18	sorting nexin 18	3.241519186	9.19E-03
P11234	RALB*	RAS like proto-oncogene B	3.213022115	1.11E-02
P04083	ANXA1*	annexin A1	3.737734985	1.70E-02
O75131	CPNE3*	copine 3	3.519118176	1.75E-02
Q9UHR4	BAIAP2L1*	BAI1 associated protein 2 like 1	3.853405556	1.88E-02
P07948	LYN*	LYN proto-oncogene, Src family tyrosine kinase	3.312438806	3.46E-02
O14773	TPP1*	tripeptidyl peptidase 1	2.955336824	3.64E-02
Q9HD42	CHMP1A	charged multivesicular body protein 1A	3.424915151	3.71E-02
Q15833	STXBP2	syntaxin binding protein 2	2.937643416	4.03E-02
Q8N271	PROM2	prominin 2	3.084670129	4.34E-02
Q9H4M9	EHD1*	EH domain containing 1	2.609803687	4.42E-02
P35579	MYH9	myosin heavy chain 9	2.853361886	4.77E-02
Q15286	RAB35*	RAB35, member RAS oncogene family	3.052396307	4.77E-02
Q9H4G4	GLIPR2	GLI pathogenesis related 2	3.568686053	4.78E-02
Q8N5I2	ARRDC1	arrestin domain containing 1	2.920264824	4.78E-02

*=Cancer-associated proteins
